# Burden of Non-communicable Diseases Attributable to High and Low Ambient Temperatures, 1990–2031: A Forecasting Analysis for GBD 2021

**DOI:** 10.1007/s11596-025-00148-7

**Published:** 2025-12-09

**Authors:** Li Pu, Huan-huan Wang, Xiang-wei Cheng, Li-bo Luo, Xiao-qing Zhang, Xia Hu, He-qi Peng, Lu Ding, Bao-zhu Xiao, Wen Zhang, Xiao-li Wang, Pei-hong Wang

**Affiliations:** 1 Department of Obstetrics and Gynecology, Union Hospital, Tongji Medical College, Huazhong University of Science and Technology, Wuhan, 430022 China; 2 School of Nursing, Tongji Medical College, Huazhong University of Science and Technology, Wuhan, 430030 China; 3 Department of Urology, The Fifth Affiliated Hospital, Sun Yat-sen University, Zhuhai, 519000 China; 4 Wuchang University of Technology, Wuhan, 430223 China; 5 Wuhan No.1 Hospital, Wuhan, 430022 China

**Keywords:** Non-communicable diseases, High temperature, Low temperature, Global Burden of Disease, Climate change, Forecasting, GBD 2021

## Abstract

**Objective:**

Non-communicable diseases (NCDs), characterized by long duration, gradual progression, and high morbidity, have emerged as a fundamental threat to global public health. Furthermore, dramatic climate change may exacerbate existing trends that worsen the burden of NCDs. Therefore, this study aimed to systematically investigate the patterns and trends of NCDs attributed to nonoptimal temperatures from 1990 to 2021.

**Methods:**

We utilized data from the Global Burden of Disease Study (GBD) 2021 to assess the temporal trends in age-standardized rates (ASR) of deaths and disability-adjusted life-years (DALYs) related to nonoptimal temperature-associated NCDs across 204 countries and territories from 1990 to 2021. Decomposition analysis was applied to quantify the contribution of key factors to this burden. The autoregressive integrated moving average (ARIMA) model was employed to predict trends over the next decade.

**Results:**

Globally in 2021, NCDs attributable to high temperature (Hi-Tem) accounted for an estimated 302,464.7 deaths (95% uncertainty interval [UI]: 171,170.6, 472,625.3) and 6,947,660.6 DALYs (95% UI: 4,013,964.7, 10,611,801.7). The ASR of Hi-Tem-related NCDs deaths and DALYs increased by 35% and 34% between 1990 and 2021. Additionally, the global burden exhibited a significant declining trend in NCDs burden caused by low temperature (Lo-Tem), with 1,477,729.8 (95% UI: 1,316,829.3, 1,631,404.8) deaths and 27,797,533.3 (95% UI: 25,270,393.5, 30,766,299.9) DALYs in 2021. China and India had the highest number of deaths and DALYs for NCDs related to Hi-Tem and Lo-Tem. In 2021, the three leading causes of the NCDs burden attributable to nonoptimal temperature were ischemic heart disease, stroke, and chronic obstructive pulmonary disease. Men and older adults were consistently vulnerable to temperature, showing the greater burden of NCDs attributable to nonoptimal temperature, and aging would exacerbate this trend. The ARIMA model projected an increasing trend in Hi-Tem-related NCDs over the coming decade, while those related to Lo-Tem would show a downward trend.

**Conclusion:**

The burden of NCDs associated with Hi-Tem has conspicuously increased in recent years compared to that associated with Lo-Tem, with significant diversity across age, sex, and socio-demographic index (SDI) levels. Therefore, public health strategies should prioritize tailored interventions for heterogeneous risk profiles across vulnerable populations, integrated with climate-resilient surveillance systems and real-time adaptive response mechanisms to mitigate projected climate-mediated exacerbations of NCD burden.

**Supplementary Information:**

The online version contains supplementary material available at 10.1007/s11596-025-00148-7.

## Introduction

Non-communicable diseases (NCDs), characterized by long duration, gradual progression, and high morbidity, have emerged as the predominant threat to global public health [1]. Based on the 2021 Global Burden of Disease (GBD) study, NCDs accounted for 43 million premature deaths and 1727 million disability-adjusted life years (DALYs), accounting for the highest global burden of deaths and DALYs [2]. The interplay of genetic, physiological, behavioral, and environmental factors contributes to the development of NCDs, suggesting that these conditions can be largely prevented [3]. According to the World Economic Forum prediction, the global economic loss from NCDs will rise to US$47 trillion, equivalent to approximately 5% of the global gross domestic product (GDP) in 2010, by 2030 [4]. Thus, understanding the risk factors of NCDs is essential for informing policymaking and formulating interventions to mitigate their impact by addressing environmental factors.

Since the late 1970s, the annual global temperature has averaged a rise of around 0.2 °C per decade [5]. The Paris Agreement called on every country to strive to cap the global temperature rise at 1.5 °C compared to pre-industrial levels [6]. Unfortunately, the Global Climate Highlights 2024 report indicated that 2024 saw unprecedented global high temperatures (Hi-Tem), with a temperature 1.60 °C higher than the pre-industrial level, marking the first time the global average temperature has exceeded 1.5 °C above the pre-industrial level [5]. These realities have led to the emergence of numerous unavoidable heatwaves, breaking national or regional temperature records. A growing body of evidence indicates an association between Hi-Tem and adverse effects on public health, particularly concerning mortality and morbidity from NCDs [7–9]. A meta-analysis reported that heat waves were significantly positively associated with cardiovascular mortality (risk estimates: 1.15, 95% confidence interval [CI]: 1.09, 1.21) [10]. Konstantinoudis et al. found that every 1 °C rise in temperature above 23.2 °C was associated with a 1.42% increase in hospitalization due to chronic obstructive pulmonary disease, through a nationwide case-crossover study from England [11]. A nationwide case-crossover study in Brazil reported that for every 5 °C increase in daily mean temperature, the hospitalization risk for diabetes increased by 6% [12]. Low temperature (Lo-Tem), while less extensively studied than Hi-Tem, still has a non-negligible effect on specific diseases, especially NCDs [13, 14].

Several global studies have characterized the burden of NCDs attributable to non-optimal temperatures between 1990 and 2019 [13, 15, 16]. For instance, research indicated that non-optimal temperatures contributed to 1,194,196 (95% UI: 963,816–1,425,090) cardiovascular disease deaths in 2019 [16], with NCDs collectively accounting for 47% of the total mortality and DALY burden from Hi-Tem [15]. However, relatively few studies have systematically evaluated the trends across the entire range of NCDs [13], as most existing literature is fragmented, focusing either on specific temperature extremes (Hi-Tem or Lo-Tem) or on individual NCD subtypes. Notably, this research gap urgently requires attention, particularly as the coronavirus disease 2019 (COVID-19) pandemic has been shown to amplify population-level susceptibility to temperature-driven NCD risks through prolonged immune dysregulation. Thus, understanding recent changes in the burden of NCDs attributable to nonoptimal temperature is critical for formulating targeted public health and climate policies.

In this study, we explored the relationship between nonoptimal temperatures (Hi-Tem and Lo-Tem) and the burden of NCDs at global, regional, and national levels during 1990–2021, stratified by the socio-demographic index (SDI), sex, and age. We extracted data from the GBD 2021 study. Our objective was to quantify NCD-related deaths and DALYs attributable to nonoptimal temperatures and project trends over the next decade. Overall, these findings will provide valuable data to support policymakers in refining healthcare strategies and rationally allocating medical and health resources, thereby advancing future efforts to address NCDs amid climate change.

## Materials and Methods

### Data Collection

The GBD 2021 estimated the global burden across 204 countries and territories from 1990 to 2021, encompassing 371 diseases and injuries alongside 88 risk factors [2, 17]. We extracted estimates of comprehensive epidemiological indicators attributable to Hi-Tem and Lo-Tem, including deaths, DALYs, and their corresponding age-standardized rates (ASR), from the GBD 2021 at global, regional, and national levels during 1990–2021, stratified by SDI, age, and sex. The 95% uncertainty interval (UI) was reported as the 2.5th and 97.5th values across ordered 1000 draws in GBD 2021 [2]. The SDI, a summary indicator, reflects the comprehensive average of the rankings for per capita income, average educational attainment, and total fertility rate [2]. To account for background social and economic conditions that affect health outcomes in each location, SDI was calculated using absolute scales to obtain a value between 0 and 1, after which it was multiplied by 100 for reporting [2].

### Definition

GBD derived temperature estimates from the European Centre for Medium-Range Weather Forecasts Reanalysis v5 (ERA5), which could provide surface temperatures with a 0.25° × 0.25° spatial resolution per hour and uncertain estimates for these temperatures with a spatial resolution of 0.5° × 0.5° every three hours [13, 17, 18]. The theoretical minimum risk exposure level (TMREL) was defined as the daily temperature that led to the lowest mortality rate for each specific location and year [13]. Given the disparity in mean annual temperature zones and the spatial and temporal causes of death, TMREL was estimated for each specific location and year [17, 19]. Hi-Tem or Lo-Tem refers to ambient temperature above or below the TMREL.

In GBD 2021, the classification of NCDs attributable to Hi-Tem and Lo-Tem was divided into four levels. NCDs themselves constituted the first level. Level 2 included cardiovascular diseases, chronic respiratory diseases, diabetes, and kidney diseases. Level 3 delved into more detailed causes under Level 2, such as cardiomyopathy and myocarditis, ischemic heart disease, stroke, and hypertensive heart disease within cardiovascular diseases. Level 4 delved into even finer details of Level 3 causes, such as type 1 diabetes mellitus and type 2 diabetes mellitus within diabetes mellitus. GBD 2021 defined NCDs attributable to Hi-Tem and Lo-Tem using the International Classification of Diseases codes (details in Table S1).

### Statistical Analysis

We utilized age-standardized mortality rates (ASMR) and age-standardized DALY rates (ASRDALYs) to estimate the burden of NCDs attributable to Hi-Tem and Lo-Tem, as well as their secular trends from 1990 to 2021. Beta coefficients (β), which indicate the average annual change in ASMR or ASRDALYs due to Hi-Tem and Lo-Tem, were calculated by fitting linear regression models to analyze trends in the burden of NCDs [20].

We leveraged locally estimated scatterplot smoothing (loess) via the “geom_smooth” function in the “ggplot2” package to fit the correlation between the burden of NCDs and SDI across 21 regions and 204 countries and territories. Additionally, Spearman correlation analysis was used to obtain the r indices and *P* values for the relationship between ASR and SDI. Decomposition analysis was employed to capture changes in the burden of NCDs associated with Hi-Tem or Lo-Tem, quantifying the effects of population growth, aging, and epidemiological changes [21].

Furthermore, to forecast the burden of NCDs attributable to Hi-Tem and Lo-Tem globally over the next 10 years, we constructed an autoregressive integrated moving average (ARIMA) model. The ARIMA model is widely used to predict future trends based on observed values. It combines autoregressive (AR), integration (I), and moving average (MA) components, thereby providing a robust framework for time series forecasting. The ARIMA model parameters (p, d, q) were automatically optimized using the “auto.arima” function by minimizing the corrected Akaike Information Criterion (AIC), with no significant residual autocorrelation confirmed by the Ljung-Box test (*P* > 0.05) [22]. All statistical analyses were performed using R software (version 4.3.2). A two-sided *P* < 0.05 was considered statistically significant.

## Results

### Overall Impact of Hi-Tem and Lo-Tem

Globally in 2021, NCDs attributable to Hi-Tem accounted for an estimated 302,464.7 deaths (95% UI: 171,170.6, 472,625.3) with an ASMR of 3.6 per 100,000 population (95% UI: 2.0, 5.6), and an estimated 6,947,660.6 DALYs (95% UI: 4,013,964.7, 10,611,801.7) with an ASRDALYs of 81.3 per 100,000 population (95% UI: 47.0, 124.3). The global ASMR and ASRDALYs of NCDs attributable to Hi-Tem exhibited a significant increase from 1990 to 2021 (ASMR: β = 0.044, 95% UI: 0.031, 0.056; ASRDALYs: β = 0.942, 95% UI: 0.665, 1.219), corresponding to increases of 35% (15%, 79%) and 34% (14%, 75%), respectively. However, the ASMR and ASRDALYs for NCDs attributable to Hi-Tem decreased by 17% (12%, 22%) from 2019 to 2021 (Table S2).

**Fig. 1:**
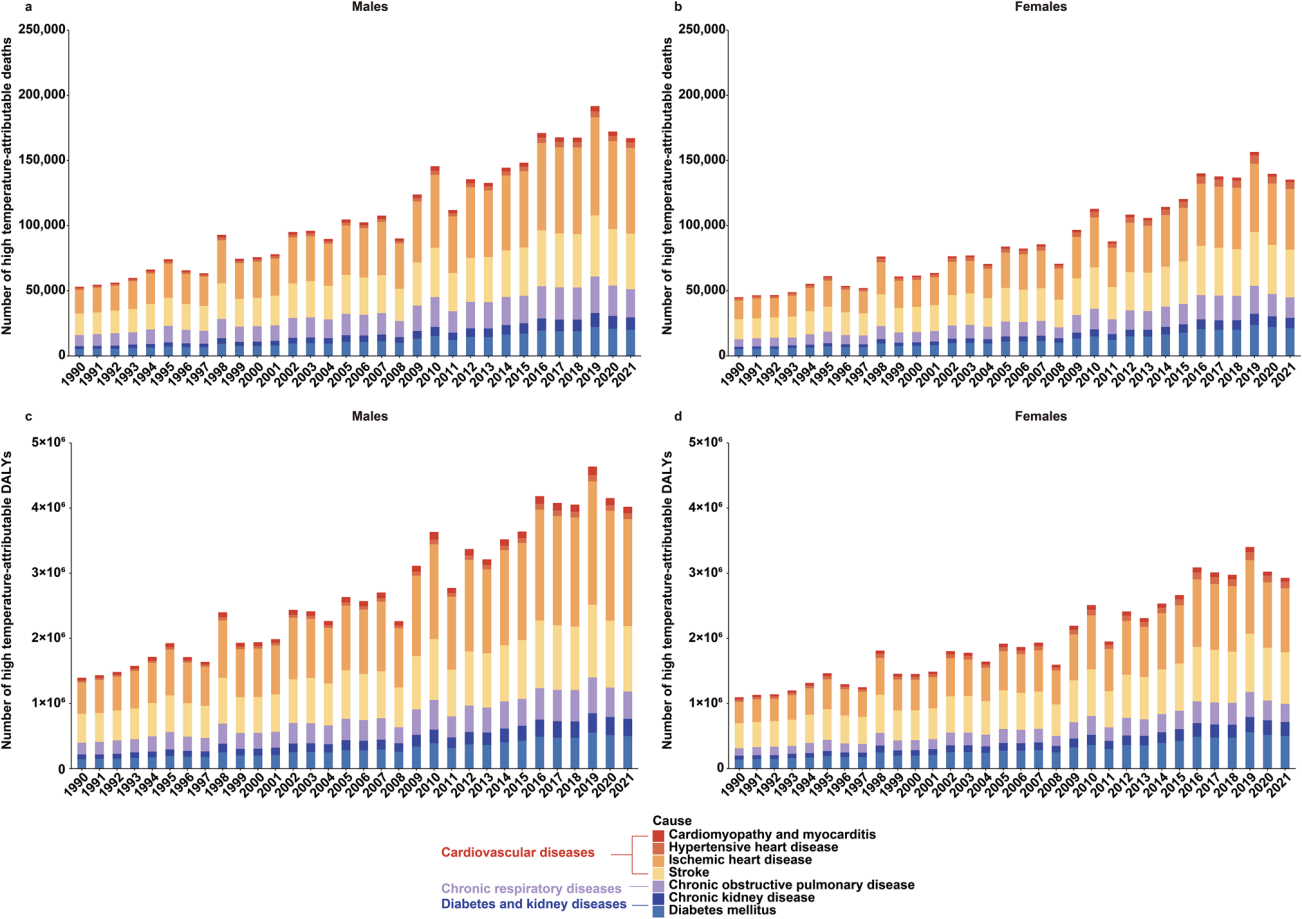
Numbers of all-age deaths and disability-adjusted life-years (DALYs) in non-communicable diseases (NCDs) attributable to high temperature by sex from 1990 to 2021 globally. **a** Numbers of all-age deaths in males; **b** Numbers of all-age deaths in females; **c** Numbers of all-age DALYs in males; **d** Numbers of all-age DALYs in females

There was a distinct discrepancy in global deaths and DALYs linked to Hi-Tem between males and females. Specifically, global deaths due to Hi-Tem among males rose from 53,102.1 (95% UI: 22,873.3, 90,575.5) in 1990 to 167,128.9 (95% UI: 92,923.3, 264,023.7) in 2021, a nearly 3.15-fold increase. For females, deaths increased from 45,073.2 (95% UI: 20,242.8, 78,035.5) in 1990 to 135,335.8 (95% UI: 76,889.9, 211,042.1) in 2021, a nearly three-fold increase. Global DALYs related to Hi-Tem among males rose from 1,395,509.6 (95% UI: 632,096.0, 2,336,917.7) in 1990 to 4,019,946.0 (95% UI: 2,282,700.4, 6,225,095.6) in 2021, a nearly 2.88-fold increase. For females, DALYs increased from 1,095,295.8 (95% UI: 514,351.7, 1,802,828.7) in 1990 to 2,927,714.7 (95% UI: 1,713,517.0, 4,439,118.4) in 2021, a nearly 2.67-fold increase (Fig. 1).

Contrary to Hi-Tem, global deaths and DALYs of NCDs attributable to Lo-Tem in 2021 were 1,477,729.8 (95% UI: 1,316,829.3, 1,631,404.8) and 27,797,533.3 (95% UI: 25,270,393.5, 30,766,299.9), respectively, with a prominent decreasing trend from 1990 to 2021 (ASMR: β = − 0.491, 95% UI: − 0.522, − 0.460; ASRDALYs: β = − 9.699, 95% UI: − 10.299, − 9.099) (Table S3). The burden of Lo-Tem-related NCDs also varied between males and females. Although the ASMR and ASRDALYs of Lo-Tem-related NCDs showed decreasing trends, the number of global deaths attributable to Lo-Tem among males rose from 518,784.1 (95% UI: 476,543.9, 568,022.8) in 1990 to 786,675.9 (95% UI: 705,841.6, 879,465.3) in 2021, a nearly 1.52-fold increase. For females, deaths increased from 517,445.0 (95% UI: 461,994.2, 563,816.6) in 1990 to 691,053.9 (95% UI: 589,240.8, 769,557.2) in 2021, a nearly 1.33-fold increase (Fig. S1). The trend in the global number of DALYs exhibited a similar pattern.

Across the 21 GBD regions, North Africa and the Middle East exhibited the highest ASMR and ASRDALYs of Hi-Tem-related NCDs: 13.1 per 100,000 population (95% UI: 6.8, 21.3) and 275.7 per 100,000 population (95% UI: 145.7, 444.6) (Table S2). Meanwhile, Central Asia reported the highest ASMR and ASRDALYs of Lo-Tem-related NCDs: 39.4 per 100,000 population (95% UI: 35.4, 44.0) and 753.0 per 100,000 population (95% UI: 676.5, 845.1) (Table S3).

Additionally, North Africa and the Middle East had the highest ASMR and ASRDALYs of Hi-Tem-related NCDs among both males and females (Table S4, S5). Conversely, Central Asia exhibited the highest ASMR and ASRDALYs of Lo-Tem-related NCDs among both males and females (Table S6, S7).

**Fig. 2:**
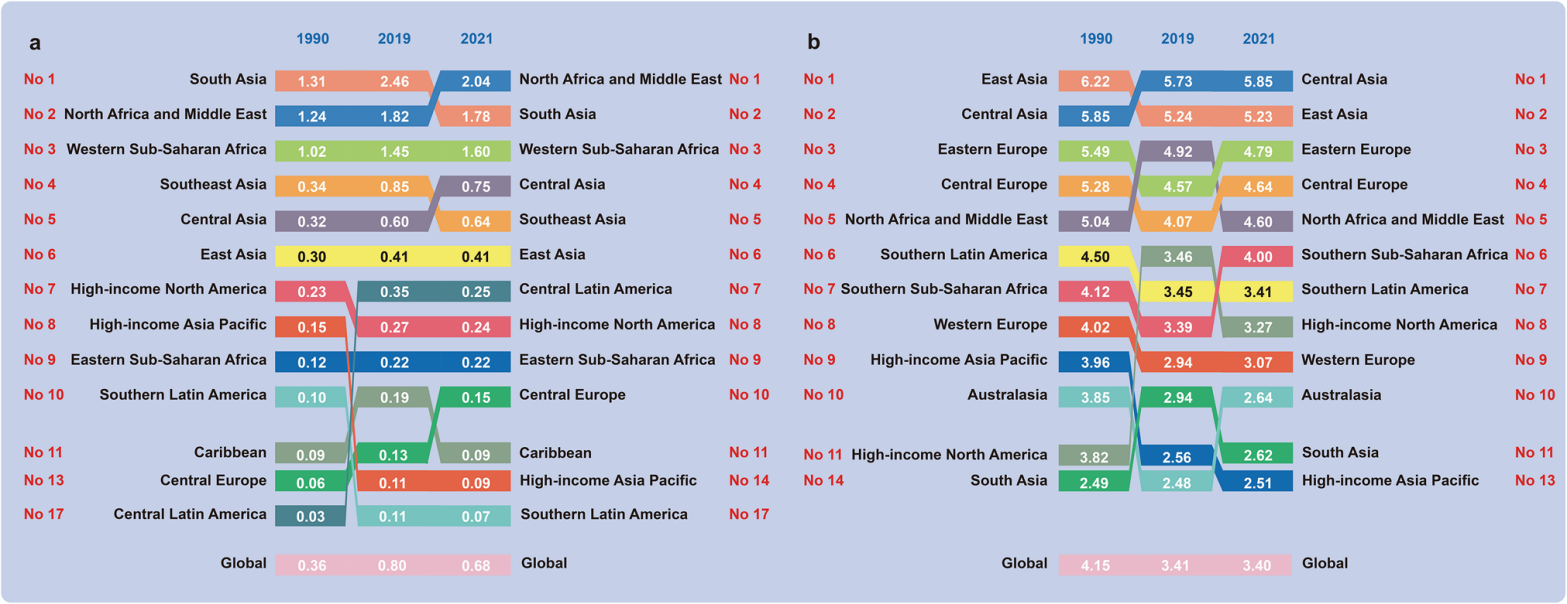
Top 10 regions with the highest proportion of non-communicable disease deaths attributable to high temperature and low temperature in 1990, 2019, and 2021 globally. **a** High temperature; **b** Low temperature

In 2021, North Africa and the Middle East had the highest proportion of NCD deaths attributable to Hi-Tem (2.04%, 95% UI: 1.07%, 3.24%), followed by South Asia (1.78%, 95% UI: 1.06%, 2.54%) and Western Sub-Saharan Africa (1.60%, 95% UI: 1.14%, 2.05%). Among the top 10 regions, Central Latin America showed the largest increase in the proportion of Hi-Tem-attributable NCD deaths from 1990 to 2021. Furthermore, Central Asia had the highest proportion of NCD deaths attributable to Lo-Tem in 2021 (5.85%, 95% UI: 5.40%, 6.43%) (Fig. 2).

### Impact of Hi-Tem and Lo-Tem on Different GBD Level Causes

In 2021, among Level 3 causes, ischemic heart disease and stroke were the two leading contributors to Hi-Tem- and Lo-Tem-related NCD deaths and DALYs (Fig. S3, S4). The ASMR values were as follows: 1.34 per 100,000 population (95% UI: 0.20, 3.07) for Hi-Tem-related ischemic heart disease; 0.94 per 100,000 population (95% UI: 0.12, 2.25) for Hi-Tem-induced stroke; 6.14 per 100,000 population (95% UI: 5.23, 7.53) for Lo-Tem-associated ischemic heart disease; and 5.01 per 100,000 population (95% UI: 4.29, 5.85) for Lo-Tem-attributable stroke (Table S8).

In 2021, across Level 3 and 4 causes, diabetes mellitus surpassed intracerebral hemorrhage to become the third leading cause of Hi-Tem-related deaths, with an ASMR of 0.49 per 100,000 population (95% UI: 0.16, 0.88). Additionally, chronic obstructive pulmonary disease was a major burden in Lo-Tem-related deaths and DALYs, with an ASMR of 3.69 per 100,000 population (95% UI: 2.97, 4.48) and an ASRDALYs of 59.22 per 100,000 population (95% UI: 47.58, 72.37).

From 1990 to 2021, chronic kidney disease showed the largest relative increase in Hi-Tem-related death rates (100%, 95% UI: − 197% to 428%) and DALY rates (94%, 95% UI: − 244% to 399%). Conversely, subarachnoid hemorrhage exhibited the largest relative decline in Lo-Tem-related death rates (− 64%, 95% UI: − 72% to − 50%) and DALY rates (-67%, 95% UI: − 73% to − 56%) over the same period (Fig. S2, S3).

### Disparities in Hi-Tem-Related and Lo-Tem-Related NCD Burden Across Global Age Groups

**Fig. 3:**
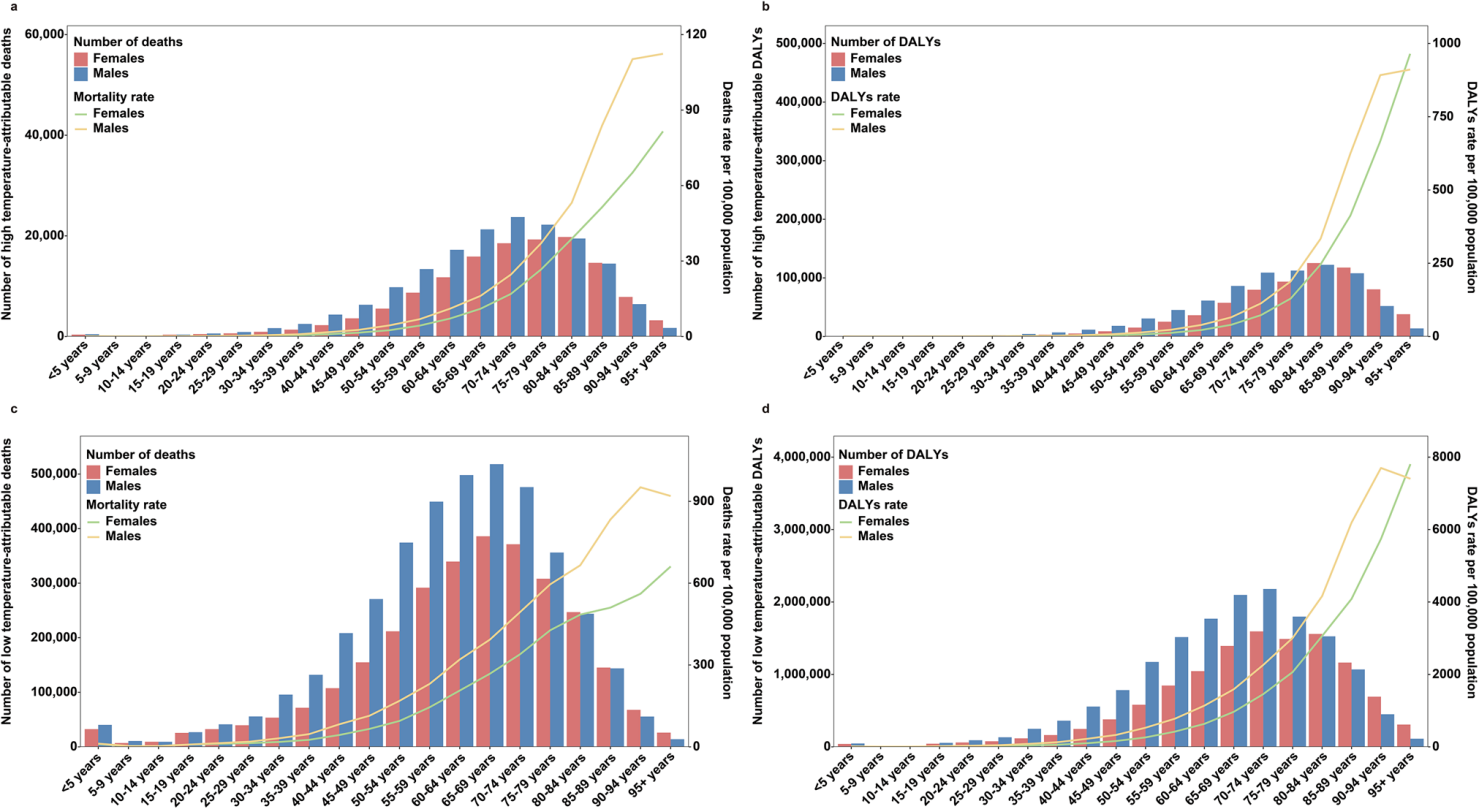
Numbers and rates of all-age deaths and DALYs in NCDs attributable to high temperature and low temperature from 1990 to 2021 in different age and sex groups globally.** a** Numbers and rates of high temperature-related NCDs deaths; **b** Numbers and rates of high temperature-related NCDs DALYs; **c** Numbers and rates of low temperature-related NCDs deaths by age groups; **d** Numbers and rates of low temperature-related NCDs DALYs

There were disparities in the Hi-Tem-related and Lo-Tem-related NCD burden across age groups. Notably, Hi-Tem-related NCD deaths peaked predominantly in males aged 70–74 years and females aged 80–84 years, whereas the highest burden of Lo-Tem-related NCD deaths occurred in both sexes in the 65–69-year age group. Furthermore, the 80–84-year age group exhibited the highest burden of DALYs attributable to Hi-Tem-related NCDs. Females had a higher number of Hi-Tem- and Lo-Tem-related NCD DALYs when aged 80 years and above. Additionally, the rates of Hi-Tem- and Lo-Tem-related deaths and DALYs showed an increasing trend with age for both males and females (Fig. 3).

In 2021, among Level 2 causes, cardiovascular diseases were the leading cause of Hi-Tem- and Lo-Tem-related deaths and DALYs across all age groups. For each GBD Level 3 cause, the highest proportion of Hi-Tem-related and Lo-Tem-related deaths and DALYs attributed to cardiomyopathy and myocarditis was observed in the age group < 5 years (Fig. S4, S5).

### Temporal Trends of NCD Burden Caused by Hi-Tem and Lo-Tem at Global and SDI Levels Over the Past 30 Years

**Fig. 4:**
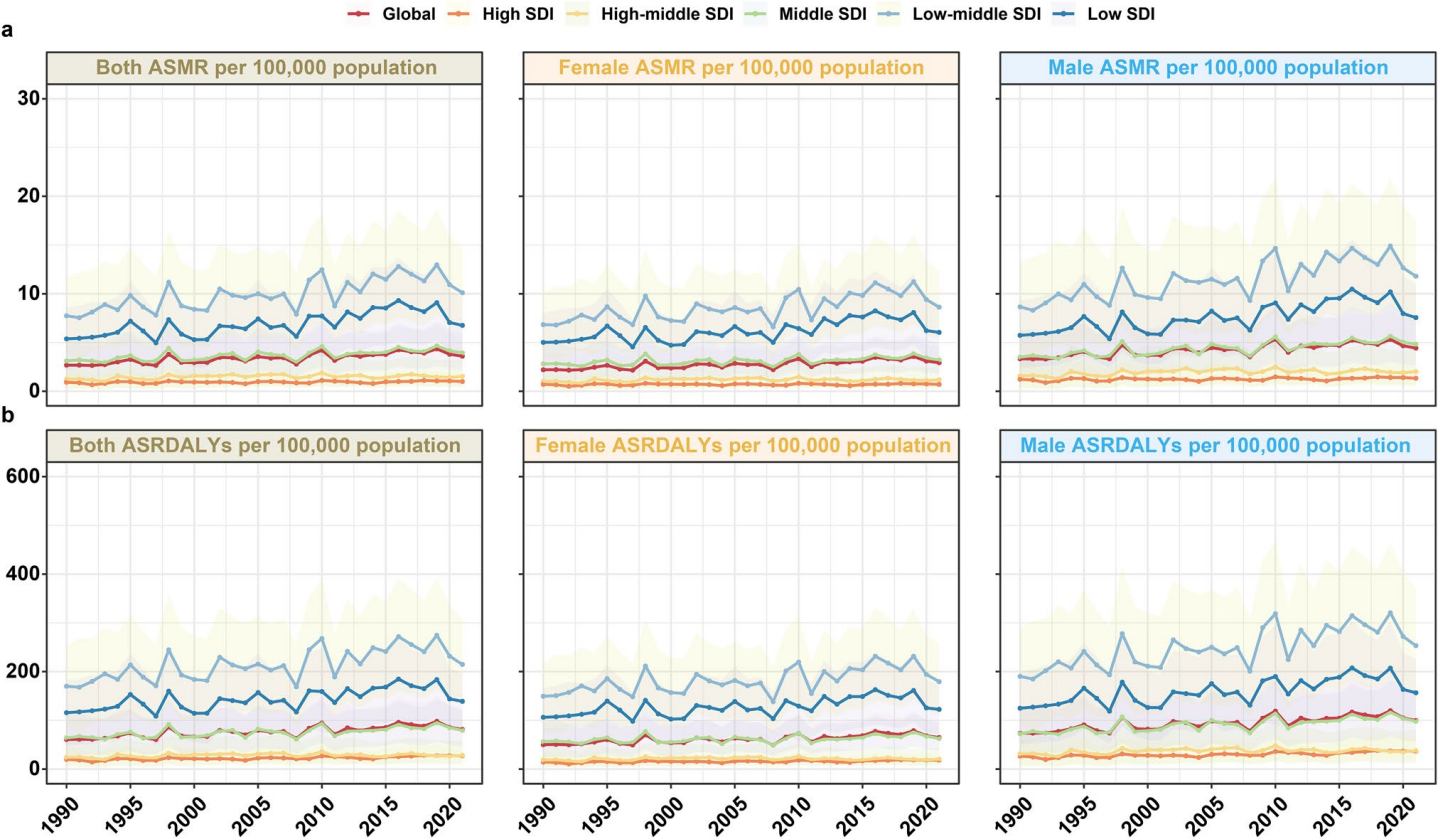
Temporal trends of NCDs burden due to high temperature in sex, global, and socio-demographic index level from 1990 to 2021. **a** The age-standardized mortality rate of NCDs attributable to high temperature; **b** The age-standardized rate of disability-adjusted life-years due to NCDs associated with high temperature. ASMR, age-standardized mortality rates; ASRDALYs, age-standardized rates of DALYs; DALYs, disability-adjusted life-years; SDI, socio-demographic index

From 1990 to 2021, the global burden of NCDs attributable to Hi-Tem showed a significant overall increase (*P* < 0.05), albeit with interannual fluctuations. This upward trend was consistent across all SDI strata, except in the high-middle SDI stratum where ASRDALYs exhibited non-significant changes (β = 0.132, 95% UI: − 0.003 to 0.267). Across SDI regions, the burden of Hi-Tem-related NCDs was concentrated primarily in low-middle SDI regions, a pattern consistent among both males and females (Fig. 4).

In contrast, the global and SDI-level burden of NCDs attributable to Lo-Tem showed a significant declining trend (*P* < 0.001). The burden of Lo-Tem-related NCDs was disproportionately concentrated in high-middle SDI regions. Notably, these regions experienced the most pronounced declines between 1990 and 2021, with ASMR decreasing by 1.74 times and ASRDALYs declining by 1.85 times over the period (Fig. S6).

### National Trends of NCD Burden Caused by Hi-Tem and Lo-Tem

In 2021, India had the highest number of Hi-Tem-related NCD deaths and DALYs, estimated at 104,887.8 (95% UI: 63,793.1, 150,538.6) and 2,477,660.1 (95% UI: 1,530,638.3, 3,521,542.2), respectively, followed by China and Pakistan (Table S9). Meanwhile, Iraq recorded the highest ASMR (56.9 per 100,000 population, 95% UI: 29.3, 89.8) and ASRDALYs (1088.1 per 100,000 population, 95% UI: 562.6, 1757.1) (Table S9).

Considerable disparities were also observed in the Lo-Tem-related NCD burden. Specifically, China had the largest number of Lo-Tem-related NCD deaths (549,968.4, 95% UI: 463,887.6, 649,295.2) and DALYs (9,641,543.0, 95% UI: 7,981,159.0, 11,429,410.1), followed by India and the United States (Table S10). Lesotho had the highest ASMR (54.6 per 100,000 population, 95% UI: 43.8, 67.0) and ASRDALYs (1146.8 per 100,000 population, 95% UI: 903.8, 1437.4) (Table S10).

### Correlation between the Burden of Hi-Tem-Related and Lo-Tem-Related NCDs and SDI

Across the 21 regions, the overall ASMR and ASRDALYs of Hi-Tem-related NCDs decreased with increasing SDI (*P* < 0.001). In contrast to Hi-Tem, the overall ASMR and ASRDALYs of Lo-Tem-attributable NCDs first increased and then decreased when SDI exceeded approximately 0.7 (Fig. S7).

At the national level, the pattern of overall ASMR and ASRDALYs of Hi-Tem-related NCDs was similar to that observed across the 21 regions, with both decreasing as SDI rose (Fig. S8a, S8b). However, no significant correlation was observed between the burden of Lo-Tem-related NCDs and SDI (Fig. S8c, S8d).

### Decomposition Analysis of Hi-Tem-Related and Lo-Tem-Related NCDs at Global and SDI Levels

Decomposition analysis indicated that age structure, population growth, and epidemiological changes contributed 38.8%, 34.8%, and 26.4%, respectively, to the global increase in Hi-Tem-related NCD deaths in 2021. Population aging emerged as the predominant driver of the Hi-Tem-related NCD burden, with its influence strengthening as SDI increased.

**Fig. 5:**
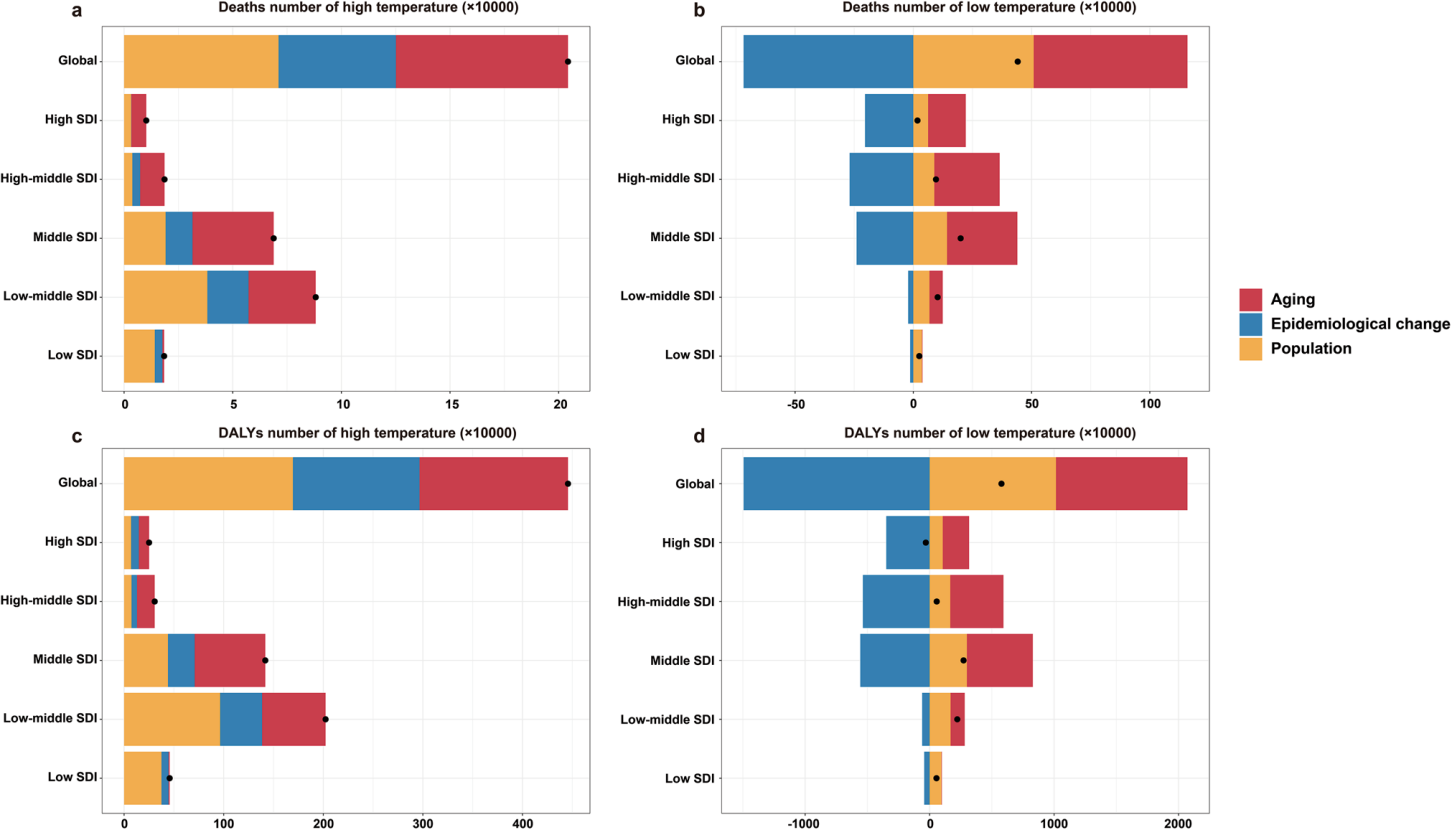
Decomposition analysis of high- and low-temperature-related NCDs across global and socio-demographic index levels.** a** High temperature-related NCDs deaths; **b** Low temperature-related NCDs deaths; **c** High temperature-related NCDs DALYs;** d** Low temperature-related NCDs DALYs. SDI, socio-demographic index

Notable differences were observed in the decomposition results of Lo-Tem-attributable NCD deaths and DALYs compared to those of Hi-Tem. While aging and population growth positively contributed to the increase in Lo-Tem-related NCD deaths and DALYs, epidemiological changes mitigated the overall burden of Lo-Tem-caused NCDs (Fig. 5 and Table S11).

### Predicted Trends Based on the ARIMA Models

The burden of Hi-Tem-attributable NCDs is projected to increase significantly over the next decade, while the corresponding burden linked to Lo-Tem is anticipated to decline. Specifically, by 2031, the Hi-Tem-related NCD burden for both sexes will reach an ASMR of 4.3 and an ASRDALYs of 97.63 per 100,000 population.

Furthermore, by 2031, the Hi-Tem-associated ASMR for cardiovascular diseases, chronic respiratory diseases, and diabetes and kidney diseases is projected to rise to 2.91, 0.54, and 0.89 per 100,000 population, respectively. The corresponding ASRDALYs are expected to reach 66.00, 9.91, and 21.82 per 100,000 population, underscoring consistent burden patterns across these NCD categories (Fig. S9a, S9b).

In contrast, the Lo-Tem-related NCD burden is projected to decline significantly by 2031, with ASMR and ASRDALYs decreasing by 14.00 and 249.49 per 100,000 population, respectively. All major NCD categories, including cardiovascular diseases, chronic respiratory diseases, and diabetes and kidney diseases, exhibit this consistent declining trajectory (Fig. S9c, S9d).

## Discussion

This study provides a detailed and comprehensive estimation of the burden of NCDs caused by Hi-Tem and Lo-Tem from 1990 to 2021. These valuable insights indicate that the Lo-Tem-related NCD burden was higher than that of Hi-Tem, while the number of deaths and DALYs associated with both Hi-Tem and Lo-Tem has increased drastically. Additionally, males and older populations bore a greater burden of Hi-Tem- and Lo-Tem-attributable NCDs. Significant disparities existed in the Hi-Tem-related and Lo-Tem-related NCD burden across regions, alongside distinct trends for specific diseases. Furthermore, based on the ARIMA model results, the Hi-Tem-related NCD burden is projected to rise over the next decade. This study explores the changing burden of NCDs attributable to Hi-Tem and Lo-Tem on a global scale, providing a valuable basis for the equitable allocation of limited health resources and the development of NCD prevention and control strategies amid climate change.

Climate change poses tremendous challenges to global public health, as the risk of exposure to high temperatures increases significantly with sharp rises in ambient temperature [23]. Growing epidemiological evidence has revealed a U-shaped relationship between ambient temperature and NCDs, indicating that Lo-Tem also exerts a considerable influence on the NCD burden [24, 25]. Cardiovascular diseases, particularly ischemic heart disease and stroke, were the leading causes of NCDs attributable to both Hi-Tem and Lo-Tem. Additionally, Hi-Tem-related diabetes and Lo-Tem-attributable chronic obstructive pulmonary disease also contributed substantially to the overall NCD burden. The mechanisms by which Hi-Tem triggers NCD events may involve dyslipidemia [26], dehydration, increased blood viscosity [27], and reduced blood flow [12]. Conversely, temperature decline can lead to peripheral vasoconstriction, elevated arterial pressure [28], increased inflammation levels [29, 30], vasoconstriction of the respiratory tract mucosa, and suppressed immune responses [31].

Our study observed that the burden of Lo-Tem-related NCDs was higher than that of Hi-Tem-related NCDs, which aligns with previous findings [13, 24, 25]. Guo et al. used a case-crossover design in Tianjin, China, and found that Lo-Tem had a stronger impact on NCD mortality than Hi-Tem among populations living at similar latitudes [24]. A study covering 384 locations across 12 countries also reported that most of the mortality burden was attributable to Lo-Tem [25]. The observed discrepancy between Lo-Tem and Hi-Tem may stem from the delayed effects of cold, resulting in longer durations of physiological responses induced by Lo-Tem compared to Hi-Tem [32, 33]. Although the burden of Hi-Tem-related NCDs is lower than that of Lo-Tem-related NCDs, the ASMR and ASRDALYs associated with Hi-Tem-related NCDs show an upward trend. Notably, 2024 saw record-breaking temperatures across all continents except Australasia and Antarctica [5]. Furthermore, projections from the ARIMA model suggest a sustained upward trajectory for Hi-Tem-related NCD burdens over the next decade. Therefore, it is imperative to adopt flexible policies and strategies to address the threat posed by climate change.

In general, men experienced a higher burden of Hi-Tem-related and Lo-Tem-related NCDs compared to women, which is consistent with previous studies [34, 35]. This sex difference may be attributed to the fact that men have more unhealthy lifestyles (such as smoking and alcohol consumption) than females, and these behaviors are pivotal contributors to the development of NCDs [36]. Additionally, sex roles, a multifaceted construct encompassing cultural norms and psychosocial attributes, play a role in determining exposure to outdoor temperatures [37]. Men are more likely to engage in outdoor physical labor in nonoptimal environments, consequently increasing their associated risk [38, 39].

Our study revealed that older adults have higher mortality and DALY rates than younger adults. Decomposition analysis further confirmed that population aging was the main driver of the burden of NCDs attributable to Hi-Tem and Lo-Tem, especially in high and high-middle SDI regions. According to the World Health Organization (WHO), between 2015 and 2050, the world’s population aged 60 years and above is projected to nearly double, increasing from 12 to 22% of the global population [40]. This rapid demographic aging poses substantial public health challenges, particularly amid climate change and the growing frequency of temperature extremes.

Older adults are consistently identified as one of the most vulnerable subgroups to nonoptimal temperatures [41]. Physiologically, they exhibit diminished thermoregulatory capacity, including reduced vasoconstrictor responsiveness, blunted shivering thermogenesis [42], and decreased sweating [43], which compromises their ability to maintain core temperature stability. These physiological declines are often exacerbated by a high burden of chronic cardiopulmonary and metabolic conditions [44]. Moreover, psychosocial factors such as social isolation and loneliness may increase their susceptibility to nonoptimal temperatures [45, 46].

We also observed that Hi-Tem-related NCDs were primarily concentrated in low- and low-middle SDI regions, while the correlations were not significant in NCDs attributable to Lo-Tem. Although air conditioning can effectively mitigate heat stress, its widespread adoption in low-income regions is constrained by chronic energy shortages and limited purchasing power [47]. In addition, compared with high SDI countries, low SDI countries still have inadequate coverage of health services and fragile healthcare systems [48], which has contributed to an increase in the incidence and mortality of Hi-Tem-related illnesses. Less access to air conditioning, inequalities in healthcare quality, and lower healthcare awareness collectively amplify the vulnerability of low-SDI populations to Hi-Tem-related NCDs [26].

Geographical variations also played a vital role in the burden of NCDs related to temperature extremes. Regions such as North Africa, the Middle East, and South Asia, largely characterized by tropical climates, face consistently high heat exposure. Over the past two decades, Africa has experienced repeated record-breaking extreme climate events, warming at a rate exceeding the global average [49]. North Africa, in particular, with its hot and arid conditions, presents substantial challenges to human settlements and health [50]. Furthermore, in economically disadvantaged areas, a considerable proportion of the population is engaged in outdoor and agricultural labor, increasing their vulnerability to heat-related health risks [51]. Conversely, many upper-middle SDI countries located at higher latitudes are disproportionately exposed to Lo-Tem due to their geographic and climatic conditions [52]. These disparities highlight the urgent need to address the compounded health threats faced by vulnerable populations resulting from the interaction between socioeconomic inequalities and climate change.

Our findings on the burden of NCDs caused by non-optimal temperatures have important implications for public health strategies worldwide. In light of projected global extreme temperature scenarios, a concerted effort among policymakers, healthcare providers, and researchers is essential to mitigate the associated health threats. For instance, implementing urban greening strategies, such as green walls, green roofs, and expanded vegetation spaces, represents a key approach to mitigating the urban heat island effect. Moreover, the effectiveness of greenery is greatly amplified when combined with other measures to achieve synergistic benefits. Coupling greening with building-level interventions (e.g., cool materials, improved ventilation) can drastically reduce cooling energy demand. Further synergies are achieved by integrating greenery with stormwater management and energy systems, creating multifunctional urban spaces that deliver compounded health and environmental benefits [53]. Additionally, to build climate resilience, health systems in low-income regions must transition from passive response to proactive preparedness. A continuum of care for vulnerable populations during extreme weather events can be achieved through systems that combine climate-resilient infrastructure, a proficient community health workforce, and digital platforms enabling early warning and remote monitoring [54].

Compared with previous studies, we comprehensively evaluated the burden of NCDs attributable to Hi-Tem and Lo-Tem across global, regional, and national temporal trends. We also harnessed decomposition analysis to explore the underlying demographic disparities in the burden of temperature-related NCDs, but several limitations remain. Primarily, given the differences in medical diagnostic levels, low- and middle-income countries might be prone to underestimating the true real-world burden. Secondly, due to data limitations, we cannot assess the impact of confounding factors (e.g., hypertension and high cholesterol levels) on our results. Thirdly, projections of future NCDs attributable to non-optimal temperatures were primarily based on current trends. We did not consider potential future changes in public health policies, interventions, or advancements in medical technology. The projection results warrant caution in interpretation but are amenable to future updates with new data. Ultimately, given inherent limitations, we only explored the burden of NCDs attributable to temperature and failed to simultaneously consider other meteorological elements (e.g., air pollutants, humidity, and air pressure).

## Conclusion

Aging and climate change pose dual challenges to NCD prevention. The burden of NCDs associated with Hi-Tem has conspicuously increased in recent years compared to that associated with Lo-Tem, with significant disparities across age, sex, and SDI levels. Projection models further indicate that this rising trend will escalate over the next decade, amplifying existing health inequalities. It is imperative to strengthen international collaboration mechanisms to mitigate persisting inequalities, further exacerbated by these trends, and reasonably allocate more health resources to vulnerable groups.

## Supplementary Information

Below is the link to the electronic supplementary material.

Supplementary file1 (PDF 4910 kb)

## Data Availability

Data used for the analyses are available at http://www.healthdata.org/ and http://ghdx.healthdata.org/gbd-results-tool.
