# Supplementary material for: Burden of Non-communicable Diseases Attributable to High and Low Ambient Temperatures, 1990–2031: A Forecasting Analysis for GBD 2021
Source: Curr Med Sci. 2025 Dec 9;46(1):244–55. doi: 10.1007/s11596-025-00148-7 (PMC12948918; doi:10.1007/s11596-025-00148-7)
Supplement: Supplementary file 1 — Supplementary file1 (PDF 4910 kb) [file 11596_2025_148_MOESM1_ESM.pdf]

Online Supplementary Material

Table S1. List of International Classification of Diseases (ICD) codes mapped to non-communicable diseases (NCDs) attributable to high temperature and low temperature in GBD 2021. 3
Table S2. Deaths and DALYs attributable to non-communicable diseases associated with high temperature in 2021 and relative change from 1990 to 2021 and 2019 to 2021 7
Table S3. Deaths and DALYs attributable to non-communicable diseases associated with low temperature in 2021 and relative change from 1990 to 2021 and 2019 to 2021 10
Table S4. Non-communicable disease deaths attributable to high temperature by sex in 2021 at the region and SDI levels and relative change from 1990 to 2021 and 2019 to 2021 13
Table S5. Non-communicable diseases DALYs attributable to high temperature by sex at the region and SDI levels in 2021 and relative change from 1990 to 2021 and 2019 to 2021 16
Table S6. Non-communicable disease deaths attributable to low temperature by sex at the region and SDI levels in 2021 and relative change from 1990 to 2021 and 2019 to 2021 19
Table S7. Non-communicable diseases DALYs attributable to low temperature by sex at the region and SDI levels in 2021 and relative change from 1990 to 2021 and 2019 to 2021 22
Table S8. ASMR and ASRDALYs of cause-specific non-communicable diseases attributable to Hi-Tem and Lo-Tem in 2021, and the temporal trends from 1990 to 2021. 25
Table S9. Deaths and DALYs attributable to non-communicable diseases associated with high temperature in 204 countries and territories in 2021 and relative change from 1990 to 2021 and 2019 to 2021. 27
Table S10. Deaths and DALYs attributable to non-communicable diseases associated with low temperature in 204 countries and territories in 2021 and relative change from 1990 to 2021 and 2019 to 2021. 41
Fig. S1. Numbers of all-age deaths and disability-adjusted life-years (DALYs) in non-communicable diseases attributable to low temperature by sex from 1990 to 2021 globally 56
Fig. S2. Leading level 3 and level 4 causes of global non-communicable diseases attributable to high temperature rankings by age-standardized mortality rate (ASMR) and age-standardized rate of disability-adjusted life years (ASRDALYs) per 100,000 population, 1990 and 2021 57
Fig. S3. Leading level 3 and level 4 causes of global non-communicable diseases attributable to low temperature rankings by age-standardized

mortality rate (ASMR) and age-standardized rate of disability-adjusted life years (ASRDALYs) per 100,000 population, 1990 and 2021 58
Fig. S4. Numbers and proportion of all-age deaths and disability-adjusted life-years (DALYs) in non-communicable diseases attributable to high temperature in 2021 globally 59
Fig. S5. Numbers and proportion of all-age deaths and disability-adjusted life-years (DALYs) in non-communicable diseases attributable to low temperature in 2021 globally 60
Fig. S6. Temporal trends of non-communicable diseases (NCDs) burden due to low temperature in sex, global, and socio-demographic index level from 1990 to 2021 61
Fig. S7. Age-standardized mortality rates (ASMR) and age-standardized rates of disability-adjusted life-years (ASRDALYs) of non-communicable diseases (NCDs) due to high temperature and low temperature for 21 GBD regions, by socio-demographic index, from 1990 to 2021 62
Fig. S8. Age-standardized mortality rates (ASMR) and age-standardized rates of disability-adjusted life-years (ASRDALYs) of non-communicable diseases (NCDs) due to high temperature and low temperature for 204 countries and territories, by socio-demographic index (SDI) in 2021 63
Fig. S9. Forecasted value of global age-standardized mortality rates (ASMR) and age-standardized rates of disability-adjusted life-years (ASRDALYs) of non-communicable diseases (NCDs) due to high temperature and low temperature for the period 2022 to 2031 64

Table S1. List of International Classification of Diseases (ICD) codes mapped to non-communicable diseases (NCDs) attributable to high temperature and low temperature in GBD 2021.

ICD codes mapped to non-fatal causes and injuries in the GBD 2021							ICD codes mapped to the causes of death	
Cause ID	Cause Hierarchy Level	Cause Name	ICD 10	ICD-10 Used in Hospital/Claims Analyses	ICD 9	ICD-9 Used in Hospital/Claims Analyses	ICD 10	ICD 9
493	3	Ischemic heart disease	I20-I21.6, I21.9-I25.9	I20-I25.9	410-414.9, V17.3	410-414.9	I20-I25.9	410-414.9
494	3	Stroke	G45-G46.8, I60-I62, I62.9-I64, I64.1, I65-I69.998	I60-I69.4	430-439.6, V12.54, V17.1	430-437.9	G45-G46.8, I60-I63.9, I65-I66.9, I67.0-I67.3, I67.5-I67.6, I68.1-I68.2, I69.0-I69.3	430-435.9, 437.0-437.2, 437.5-437.8
495	4	Ischemic stroke	G45-G46.8, I63-I63.9, I65-I66.9, I67.2-I67.848, I69.3-I69.4	I63-I63.9	433-435.9, 437.0-437.2, 437.4-437.9	434-434.91	G45-G46.8, I63-I63.9, I65-I66.9, I67.2-I67.3, I67.5-I67.6, I69.3	433-435.9, 437.0-437.1, 437.5-437.8
496	4	Intracerebral hemorrhage	I61-I62, I62.9, I69.0-I69.298	I61-I62.9	431, 431.1-432.9	431, 431.1-432.9	I61-I62, I62.1-I62.9, I68.1-I68.2, I69.1-I69.2	431-432.9, 437.2

497	4	Subarachnoid hemorrhage	I60-I60.9, I67.0-I67.1	I60-I60.9, I67.0-I67.1	430-430.9, 431.0, 437.3	430-430.9, 431.0, 437.3	I60-I60.9, I62.0, I67.0-I67.1, I69.0	430-430.9
498	3	Hypertensive heart disease	I11-I11.2, I11.9		402-402.91		I11-I11.9	402-402.9
499	3	Cardiomyopathy and myocarditis	B33.2-B33.20, B33.22-B33.24, D86.85, I40-I41.8, I42-I43.8, I51.4-I51.6	B33.2-B33.20, B33.22-B33.24, I40-I41.8, I51.4-I51.6	074.2, 074.23, 422-422.99, 425-425.5, 425.7-425.9, 429.0-429.1	074.2, 074.23, 422-422.99, 429.0-429.1	B33.2, I40-I41.9, I42.1-I42.8, I43-I43.9, I51.4	422-422.9, 425.0-425.5, 425.7-425.8, 429.0
942	4	Myocarditis	B33.2-B33.20, B33.22-B33.24, D86.85, I40-I41.8, I51.4-I51.6	B33.2-B33.20, B33.22-B33.24, I40-I41.8, I51.4-I51.6	074.2, 074.23, 422-422.99, 429.0-429.1	074.2, 074.23, 422-422.99, 429.0-429.1	B33.2, I40-I41.9, I51.4	422-422.9
8	4	Alcoholic cardiomyopathy	I42.6		425.5		I42.6	425.5
944	4	Other cardiomyopathy	I42.0-I42.5, I42.7		425.0-425.18, 425.3, 425.8-425.9	410-414.9	I42.1-I42.5, I42.7-I42.8, I43-I43.9	425.0-425.4, 425.7-425.8, 429.0
509	3	Chronic obstructive pulmonary disease	J41-J42.4, J43-J44.9	J41-J44.9	491-492.9, 496-499	491-492.9, 496-499	J41-J44.9	491-492.9, 496-499

587	3	Diabetes mellitus	E08-E08.11, E08.3-E08.9, E10-E10.11, E10.3- E11.1, E11.3-E12.1, E12.3-E13.11, E13.3-E14.1, E14.3-E14.9, R73-R73.9	E08-E08.11, E08.3-E08.9, E10-E10.11, E10.3-E11.1, E11.3-E12.1, E12.3-E13.11, E13.3-E14.1, E14.3- E14.9	249-249.31, 249.5-250.39, 250.5-250.99, 362.0-362.07, 790.2-790.29, V18-V18.0, V42.83, V45.85, V58.67, V77.1	249-249.31, 249.5-250.39, 250.5-250.99, 362.0-362.07	E10-E10.1, E10.3-E11.1, E11.3-E11.9, P70.2	775.1
975	4	Diabetes mellitus type 1	E10-E10.11, E10.3-E10.9	E10-E10.11, E10.3-E10.9	250-250.0, 250.01, 250.03-250.1, 250.11, 250.13-250.2, 250.21, 250.23-250.3, 250.31, 250.33-250.39, 250.5, 250.51, 250.53-250.6, 250.61, 250.63-250.7, 250.71, 250.73 250.8, 250.81, 250.83-250.9, 250.91, 250.93-250.99	250-250.0, 250.01-250.01, 250.03-250.1, 250.11-250.11, 250.13- 250.2, 250.21-250.21, 250.23-250.3, 250.31-250.31, 250.33- 250.39, 250.5-250.5, 250.51-250.51, 250.53-250.6, 250.61-250.61, 250.63-250.7, 250.71-250.71, 250.73-250.8, 250.81-250.81, 250.83 250.9, 250.91-250.91, 250.93-250.99	E10-E10.1, E10.3-E10.9, P70.2	775.1
976	4	Diabetes mellitus type 2			250.00, 250.02, 250.10, 250.12, 250.20, 250.22, 250.30, 250.32, 250.50, 250.52, 250.60, 250.62, 250.70, 250.72, 250.80, 250.82, 250.90, 250.92	410-414.9	E11-E11.1, E11.3-E11.9	

589	3	Chronic kidney disease	D63.1, E08.2-E08.29, E10.2-E10.29, E11.2-E11.29, E12.2, E13.2-E13.29, E14.2, I12-I13.9, N02-N08.8, N15.0, N17-N19, Q60-Q63.2, Q63.8-Q63.9, Q64.2- Q64.9	D63.1, E08.2-E08.29, E10.2-E10.29, E11.2-E11.29, E12.2, E13.2-E13.29, E14.2, I12-I13.9, N02-N08.8, N15.0, N17-N19, P96.0-P96.0	249.4-249.41, 250.4-250.49, 285.21, 403-404.93, 581-587.9, 753.0-753.4, 753.6-753.9, V13.03-V13.09, V18.6, V18.69, V42.0, V45.1-V45.12, V45.73, V56-V56.8, V59.4, V81.5-V81.6	249.4-249.41, 250.4-250.49, 285.21, 403-404.93, 581-587.9	D63.1, E10.2, E11.2, I12-I13.9, N02-N08.8, N15.0, N18-N18.9, Q61-Q62.8	403-404.9, 581-583.9, 585-585.9, 589-589.9, 753-753.3

Table S2. Deaths and DALYs attributable to non-communicable diseases associated with high temperature in 2021 and relative change from 1990 to 2021 and 2019 to 2021.

**Categories**	**Deaths**						**DALYs**					
**location**	**Number of cases**	**ASMR**	**Relative change in ASMR 1990-2021**	**Relative change in ASMR 2019-2021**	**Trends of age-standardized rate from 1990 to 2021 (β)**	***P* value**	**Number of cases**	**ASRDALYs**	**Relative change in ASRDALYs 1990-2021**	**Relative change in ASRDALYs 2019-2021**	**Trends of age-standardized rate from 1990 to 2021 (β)**	***P* value**
**Global**	302464.7 (171170.6, 472625.3)	3.6 (2.0, 5.6)	0.35 (0.15, 0.79)	-0.17 (-0.22, -0.12)	**0.044 (0.031, 0.056)**	**<0.001**	6947660.6 (4013964.7, 10611801.7)	81.3 (47.0, 124.3)	0.34 (0.14, 0.75)	-0.17 (-0.22, -0.12)	**0.942 (0.665, 1.219)**	**<0.001**
**Sex**												
Female	135335.8 (76889.9, 211042.1)	2.9 (1.7, 4.6)	0.33 (0.12, 0.78)	-0.18 (-0.23, -0.12)	**0.035 (0.025, 0.045)**	**<0.001**	2927714.7 (1713517.0, 4439118.4)	64.9 (38.1, 97.8)	0.30 (0.09, 0.71)	-0.18 (-0.23, -0.11)	**0.695 (0.470, 0.920)**	**<0.001**
Male	167128.9 (92923.3, 264023.7)	4.4 (2.4, 7.0)	0.33 (0.12, 0.79)	-0.17 (-0.23, -0.10)	**0.052 (0.037, 0.068)**	**<0.001**	4019946.0 (2282700.4, 6225095.6)	99.7 (56.5, 155.1)	0.35 (0.13, 0.79)	-0.17 (-0.23, -0.10)	**1.185 (0.846, 1.524)**	**<0.001**
**Regions**												
Andean Latin America	56.5 (-14.4, 128.9)	0.1 (-0.0, 0.2)	-2.14 (-18.64, 18.21)	-0.06 (-2.79, 1.73)	**0.007 (0.005, 0.008)**	**<0.001**	1389.4 (-150.0, 3014.6)	2.3 (-0.3, 5.0)	-2.66 (-37.50, 23.44)	-0.06 (-2.09, 1.64)	**0.138 (0.112, 0.164)**	**<0.001**
Australasia	75.4 (15.2, 182.0)	0.1 (0.0, 0.3)	-0.73 (-0.84, -0.52)	-0.54 (-0.68, -0.49)	**-0.006 (-0.008, -0.004)**	**<0.001**	1117.8 (243.7, 2624.4)	2.1 (0.5, 4.8)	-0.75 (-0.84, -0.55)	-0.54 (-0.69, -0.50)	**-0.110 (-0.150, -0.069)**	**<0.001**
Caribbean	400.7 (265.2, 545.0)	0.7 (0.5, 1.0)	0.15 (-4.66, 4.04)	-0.28 (-0.35, -0.16)	-0.005 (-0.076, 0.067)	0.897	8178.0 (5238.2, 11238.9)	15.2 (9.6, 21.0)	0.17 (-3.40, 4.78)	-0.30 (-0.39, -0.16)	-0.157 (-2.054, 1.740)	0.867
Central Asia	3485.6 (1521.1, 7299.0)	5.0 (2.1, 10.9)	0.95 (0.54, 2.90)	0.21 (0.13, 0.29)	**0.058 (0.037, 0.080)**	**<0.001**	79994.1 (37382.7, 157915.8)	100.6 (45.7, 204.7)	0.93 (0.50, 2.62)	0.21 (0.12, 0.30)	**1.090 (0.618, 1.561)**	**<0.001**
Central Europe	1953.4 (388.8, 5271.9)	0.8 (0.2, 2.2)	0.49 (0.07, 6.42)	0.08 (-0.05, 0.77)	-0.002 (-0.012, 0.008)	0.738	32378.2 (6637.9, 86565.8)	14.6 (3.1, 38.9)	0.42 (0.01, 5.80)	0.08 (-0.05, 0.73)	-0.066 (-0.247, 0.115)	0.462
Central Latin America	2921.5 (1582.8, 4280.4)	1.2 (0.7, 1.8)	5.29 (-21.33, 31.27)	-0.28 (-0.35, -0.22)	**0.052 (0.035, 0.069)**	**<0.001**	66208.1 (35119.7, 96845.4)	26.2 (13.9, 38.3)	4.33 (-51.58, 21.17)	-0.26 (-0.33, -0.19)	**1.069 (0.723, 1.415)**	**<0.001**
Central Sub-Saharan Africa	74.2 (-683.9, 542.5)	0.1 (-1.7, 1.2)	-1.04 (-13.40, 4.89)	-1.50 (-3.16, 3.36)	**0.043 (0.035, 0.051)**	**<0.001**	3079.5 (-17642.8, 15663.6)	3.3 (-33.1, 25.9)	-1.10 (-11.07, 10.49)	-4.48 (-5.08, 4.33)	**0.963 (0.797, 1.130)**	**<0.001**
East Asia	43913.5 (13440.5, 93134.1)	2.4 (0.7, 5.0)	-0.17 (-0.48, 1.07)	-0.01 (-0.19, 0.17)	-0.008 (-0.025, 0.008)	0.302	779921.2 (238051.6, 1679487.9)	39.0 (12.1, 83.1)	-0.22 (-0.50, 0.73)	-0.01 (-0.20, 0.19)	-0.253 (-0.515, 0.009)	0.058
Eastern Europe	2763.0 (329.0, 8446.5)	0.8 (0.1, 2.4)	2.58 (-38.49, 37.83)	0.28 (0.05, 0.93)	**0.015 (0.004, 0.026)**	**0.007**	52104.7 (7498.3, 151979.3)	15.4 (2.3, 43.3)	2.97 (-63.95, 48.58)	0.34 (0.05, 1.06)	**0.298 (0.075, 0.521)**	**0.010**

Eastern Sub-Saharan Africa	2111.8 (890.2, 3386.4)	1.4 (0.6, 2.3)	0.51 (-2.34, 3.40)	-0.01 (-0.07, 0.14)	**0.018 (0.011, 0.025)**	**<0.001**	60627.8 (25873.6, 96674.1)	31.4 (13.3, 50.2)	0.42 (-1.71, 3.19)	-0.00 (-0.06, 0.13)	**0.349 (0.180, 0.519)**	**<0.001**
High-income Asia Pacific	1408.5 (290.0, 3438.9)	0.2 (0.1, 0.6)	-0.67 (-0.76, -0.56)	-0.16 (-0.36, -0.10)	**-0.007 (-0.012, -0.001)**	**0.021**	20806.3 (4608.3, 50602.1)	4.6 (1.1, 11.1)	-0.65 (-0.75, -0.55)	-0.16 (-0.34, -0.10)	**-0.125 (-0.225, -0.025)**	**0.016**
High-income North America	7256.1 (2202.3, 16363.4)	1.0 (0.3, 2.3)	-0.21 (-0.46, 0.61)	-0.12 (-0.18, -0.09)	-0.004 (-0.013, 0.004)	0.313	129574.1 (40621.3, 286010.1)	20.7 (6.5, 45.0)	-0.21 (-0.44, 0.49)	-0.14 (-0.20, -0.10)	-0.064 (-0.231, 0.103)	0.437
North Africa and Middle East	51803.9 (27133.6, 83869.3)	13.1 (6.8, 21.3)	0.20 (0.02, 0.50)	0.10 (0.05, 0.14)	**0.039 (0.004, 0.073)**	**0.028**	1291369.9 (686983.4, 2070497.7)	275.7 (145.7, 444.6)	0.18 (-0.01, 0.50)	0.08 (0.03, 0.14)	**0.865 (0.144, 1.586)**	**0.020**
Oceania	40.9 (21.7, 54.9)	0.6 (0.3, 0.8)	4.74 (-12.19, 24.60)	0.16 (0.02, 0.49)	**0.017 (0.013, 0.021)**	**<0.001**	1149.7 (602.4, 1554.4)	14.3 (7.5, 19.2)	4.66 (-9.02, 16.62)	0.18 (0.05, 0.49)	**0.387 (0.293, 0.481)**	**<0.001**
South Asia	142733.5 (85789.2, 204728.4)	10.9 (6.5, 15.8)	0.22 (0.03, 0.63)	-0.30 (-0.35, -0.23)	**0.131 (0.075, 0.188)**	**<0.001**	3433656.4 (2089550.1, 4926236.5)	231.3 (141.6, 330.9)	0.19 (0.01, 0.57)	-0.29 (-0.35, -0.22)	**2.389 (1.217, 3.562)**	**<0.001**
Southeast Asia	22245.4 (16609.6, 28844.7)	4.0 (3.0, 5.2)	0.65 (0.24, 2.43)	-0.25 (-0.29, -0.20)	**0.068 (0.029, 0.107)**	**0.001**	510809.6 (387085.3, 660177.4)	79.7 (60.0, 102.9)	0.57 (0.17, 2.40)	-0.26 (-0.30, -0.20)	**1.238 (0.429, 2.047)**	**0.004**
Southern Latin America	266.3 (45.1, 560.9)	0.3 (0.1, 0.6)	-0.55 (-0.75, -0.36)	-0.39 (-0.64, -0.32)	-0.002 (-0.005, 0.002)	0.420	5049.6 (997.6, 10502.4)	5.9 (1.2, 12.2)	-0.56 (-0.74, -0.40)	-0.38 (-0.62, -0.32)	-0.049 (-0.124, 0.027)	0.200
Southern Sub-Saharan Africa	250.1 (-27.6, 607.9)	0.5 (-0.1, 1.2)	0.92 (-7.22, 8.11)	-0.56 (-1.35, -0.49)	**0.018 (0.009, 0.028)**	**<0.001**	6295.1 (-475.6, 14930.8)	10.6 (-1.0, 25.5)	0.87 (-4.75, 8.48)	-0.54 (-1.10, -0.47)	**0.364 (0.172, 0.556)**	**<0.001**
Tropical Latin America	1004.1 (62.3, 2112.1)	0.4 (0.0, 0.8)	0.11 (-5.88, 3.82)	-0.51 (-0.68, -0.31)	**0.011 (0.003, 0.019)**	**0.01**	21309.2 (-4.0, 45397.5)	8.3 (0.0, 17.7)	0.14 (-5.52, 6.11)	-0.51 (-0.79, -0.33)	**0.218 (0.055, 0.381)**	**0.011**
Western Europe	1718.3 (560.9, 4263.4)	0.1 (0.0, 0.4)	-0.29 (-0.52, 0.80)	-0.27 (-0.32, -0.13)	-0.002 (-0.005, 0.000)	0.105	24129.2 (7671.3, 59749.6)	2.4 (0.8, 6.0)	-0.34 (-0.54, 0.64)	-0.28 (-0.32, -0.13)	**-0.044 (-0.087, -0.001)**	**0.044**
Western Sub-Saharan Africa	15982.0 (11094.7, 20906.8)	9.8 (6.9, 12.7)	0.38 (0.10, 1.79)	0.09 (0.02, 0.18)	**0.115 (0.075, 0.154)**	**<0.001**	418512.8 (284663.1, 561462.7)	197.9 (137.2, 258.8)	0.29 (0.02, 1.62)	0.08 (-0.00, 0.19)	**2.060 (1.242, 2.877)**	**<0.001**
**SDI**												
High SDI	20165.5 (8340.2, 38914.7)	1.0 (0.4, 1.8)	0.09 (-0.22, 0.86)	-0.06 (-0.09, -0.01)	**0.006 (0.002, 0.009)**	**0.005**	459215.2 (210657.2, 811917.8)	27.2 (12.9, 46.7)	0.37 (-0.01, 1.27)	-0.02 (-0.06, 0.03)	**0.299 (0.219, 0.378)**	**<0.001**

High-middle SDI	29220.2 (8865.8, 63227.2)	1.5 (0.5, 3.3)	0.24 (-0.14, 1.11)	0.05 (-0.08, 0.19)	**0.010 (0.002, 0.017)**	**0.012**	536865.7 (177326.4, 1136718.7)	28.2 (9.5, 59.0)	0.17 (-0.17, 0.98)	0.04 (-0.09, 0.18)	0.132 (-0.003, 0.267)	0.054
Middle SDI	95106.1 (53964.6, 146062.4)	3.9 (2.2, 6.1)	0.26 (0.01, 0.85)	-0.15 (-0.21, -0.09)	**0.037 (0.023, 0.052)**	**<0.001**	2086105.0 (1203142.8, 3165388.1)	79.5 (45.6, 120.7)	0.24 (-0.02, 0.81)	-0.17 (-0.22, -0.11)	**0.688 (0.391, 0.985)**	**<0.001**
Low-middle SDI	129257.1 (78991.5, 185416.2)	10.1 (6.2, 14.5)	0.31 (0.13, 0.71)	-0.22 (-0.27, -0.17)	**0.126 (0.084, 0.169)**	**<0.001**	3124800.9 (1917522.4, 4476579.3)	214.8 (132.6, 307.6)	0.26 (0.09, 0.68)	-0.22 (-0.28, -0.16)	**2.390 (1.483, 3.297)**	**<0.001**
Low SDI	28589.0 (17244.2, 40485.2)	6.8 (4.0, 9.7)	0.26 (0.05, 1.39)	-0.26 (-0.31, -0.19)	**0.096 (0.064, 0.128)**	**<0.001**	737876.0 (443569.4, 1042070.3)	139.0 (84.2, 195.7)	0.20 (0.01, 1.29)	-0.24 (-0.31, -0.17)	**1.558 (0.945, 2.172)**	**<0.001**

ASMR, age-standardized mortality rates; ASRDALYs, age-standardized rates of DALYs; DALYs, disability-adjusted life-years; SDI, socio-demographic index.

Table S3. Deaths and DALYs attributable to non-communicable diseases associated with low temperature in 2021 and relative change from 1990 to 2021 and 2019 to 2021.

Categories	Deaths						DALYs					
**location**	**Number of cases**	**ASMR**	**Relative change in ASMR 1990-2021**	**Relative change in ASMR 2019-2021**	**Trends of age-standardized rate from 1990 to 2021 (β)**	***P* value**	**Number of cases**	**ASRDALYs**	**Relative change in ASRDALYs 1990-2021**	**Relative change in ASRDALYs 2019-2021**	**Trends of age-standardized rate from 1990 to 2021 (β)**	***P* value**
**Global**	1477729.8 (1316829.3, 1631404.8)	18.0 (16.0, 19.9)	-0.41 (-0.45, -0.37)	-0.02 (-0.09, 0.05)	**-0.491 (-0.522, -0.460)**	**<0.001**	27797533.3 (25270393.5, 30766299.9)	328.8 (298.6, 363.6)	-0.43 (-0.47, -0.38)	-0.03 (-0.10, 0.05)	**-9.699 (-10.299, -9.099)**	**<0.001**
**Sex**												
Female	691053.9 (589240.8, 769557.2)	14.8 (12.6, 16.4)	-0.45 (-0.49, -0.39)	-0.02 (-0.10, 0.08)	**-0.468 (-0.496, -0.440)**	**<0.001**	11814698.2 (10343397.7, 13147692.2)	256.4 (224.8, 285.2)	-0.47 (-0.52, -0.41)	-0.03 (-0.11, 0.07)	**-8.803 (-9.338, -8.268)**	**<0.001**
Male	786675.9 (705841.6, 879465.3)	22.1 (19.8, 24.6)	-0.37 (-0.43, -0.31)	-0.02 (-0.13, 0.09)	**-0.521 (-0.556, -0.485)**	**<0.001**	15982835.1 (14351874.8, 17928300.4)	411.9 (370.0, 461.7)	-0.40 (-0.46, -0.33)	-0.03 (-0.14, 0.08)	**-10.739 (-11.437, -10.041)**	**<0.001**
**Regions**												
Andean Latin America	5859.7 (4931.5, 7061.0)	10.3 (8.7, 12.4)	-0.33 (-0.44, -0.19)	-0.02 (-0.12, 0.07)	**-0.181 (-0.210, -0.152)**	**<0.001**	122100.7 (101629.4, 148157.0)	205.1 (171.2, 248.6)	-0.36 (-0.48, -0.23)	-0.01 (-0.11, 0.10)	**-4.239 (-4.845, -3.634)**	**<0.001**
Australasia	5169.8 (4367.4, 5743.1)	8.4 (7.2, 9.3)	-0.62 (-0.65, -0.60)	0.02 (0.01, 0.03)	**-0.495 (-0.539, -0.452)**	**<0.001**	76239.0 (67162.1, 83618.5)	137.9 (123.6, 150.5)	-0.65 (-0.67, -0.63)	0.02 (0.00, 0.03)	**-8.993 (-9.872, -8.115)**	**<0.001**
Caribbean	1061.2 (890.0, 1258.0)	1.9 (1.6, 2.3)	-0.37 (-0.46, -0.26)	0.16 (0.06, 0.28)	**-0.053 (-0.074, -0.032)**	**<0.001**	22109.2 (18433.9, 26223.7)	41.3 (34.5, 49.0)	-0.34 (-0.43, -0.21)	0.15 (0.05, 0.27)	**-1.000 (-1.410, -0.590)**	**<0.001**
Central Asia	26741.5 (24016.3, 29890.4)	39.4 (35.4, 44.0)	-0.17 (-0.24, -0.10)	-0.01 (-0.08, 0.05)	**-0.515 (-0.656, -0.374)**	**<0.001**	585572.4 (526229.8, 659400.0)	753.0 (676.5, 845.1)	-0.20 (-0.27, -0.12)	-0.01 (-0.09, 0.06)	**-12.679 (-16.003, -9.355)**	**<0.001**
Central Europe	61728.7 (55676.1, 67219.8)	26.1 (23.6, 28.4)	-0.43 (-0.47, -0.40)	0.11 (0.05, 0.17)	**-0.903 (-0.982, -0.824)**	**<0.001**	1012538.5 (926973.6, 1104838.8)	454.4 (417.4, 496.0)	-0.46 (-0.50, -0.43)	0.11 (0.04, 0.18)	**-17.593 (-19.105, -16.082)**	**<0.001**
Central Latin America	27945.1 (25063.6, 31608.7)	11.7 (10.5, 13.2)	-0.28 (-0.34, -0.21)	0.08 (-0.00, 0.17)	**-0.173 (-0.197, -0.148)**	**<0.001**	584371.5 (523236.5, 663473.9)	233.9 (209.5, 265.5)	-0.25 (-0.32, -0.17)	0.10 (0.00, 0.20)	**-3.214 (-3.676, -2.752)**	**<0.001**
Central Sub-Saharan Africa	2842.7 (2247.2, 3511.5)	6.9 (5.5, 8.5)	-0.25 (-0.43, -0.02)	0.06 (-0.01, 0.15)	**-0.114 (-0.141, -0.087)**	**<0.001**	74620.2 (58136.2, 93138.7)	137.2 (108.6, 169.2)	-0.29 (-0.46, -0.06)	0.05 (-0.01, 0.15)	**-2.617 (-3.182, -2.051)**	**<0.001**
East Asia	564536.9 (478665.4, 664433.3)	29.9 (25.3, 35.0)	-0.50 (-0.58, -0.40)	-0.02 (-0.18, 0.16)	**-1.071 (-1.141, -1.001)**	**<0.001**	9938122.0 (8265507.5, 11740901.5)	489.2 (410.9, 576.9)	-0.53 (-0.61, -0.44)	-0.02 (-0.20, 0.19)	**-19.880 (-21.046, -18.714)**	**<0.001**

Eastern Europe	115177.8 (103289.8, 127058.7)	32.5 (29.1, 35.8)	-0.32 (-0.38, -0.25)	0.02 (-0.06, 0.11)	**-0.919 (-1.132, -0.706)**	**<0.001**	2134628.1 (1935337.0, 2364235.6)	626.0 (568.8, 691.8)	-0.29 (-0.36, -0.22)	0.01 (-0.08, 0.11)	**-17.129 (-21.989, -12.268)**	**<0.001**
Eastern Sub-Saharan Africa	17002.2 (15197.4, 19226.7)	12.5 (11.1, 14.1)	-0.31 (-0.38, -0.22)	0.10 (0.03, 0.16)	**-0.244 (-0.276, -0.211)**	**<0.001**	437576.8 (390746.6, 496021.7)	249.0 (222.5, 281.9)	-0.37 (-0.44, -0.28)	0.10 (0.03, 0.16)	**-6.128 (-6.843, -5.414)**	**<0.001**
High-income Asia Pacific	41630.4 (33116.1, 46736.3)	6.7 (5.5, 7.4)	-0.65 (-0.67, -0.64)	-0.04 (-0.04, -0.03)	**-0.416 (-0.459, -0.374)**	**<0.001**	567879.9 (477965.1, 627108.7)	114.6 (101.5, 124.4)	-0.66 (-0.67, -0.64)	-0.04 (-0.05, -0.03)	**-7.401 (-8.051, -6.751)**	**<0.001**
High-income North America	98836.7 (84891.0, 107353.5)	14.1 (12.2, 15.2)	-0.35 (-0.39, -0.33)	-0.06 (-0.07, -0.04)	**-0.331 (-0.368, -0.295)**	**<0.001**	1702494.9 (1525662.9, 1820731.0)	266.2 (242.1, 283.3)	-0.36 (-0.39, -0.34)	-0.06 (-0.08, -0.05)	**-6.290 (-6.959, -5.621)**	**<0.001**
North Africa and Middle East	108199.4 (96271.8, 122580.1)	29.5 (25.9, 33.1)	-0.34 (-0.39, -0.28)	-0.08 (-0.12, -0.05)	**-0.526 (-0.616, -0.436)**	**<0.001**	2383418.0 (2110828.0, 2746296.9)	546.4 (485.6, 621.3)	-0.39 (-0.44, -0.33)	-0.09 (-0.13, -0.05)	**-12.489 (-14.218, -10.759)**	**<0.001**
Oceania	1082.7 (846.8, 1334.7)	17.5 (13.9, 21.4)	-0.20 (-0.39, 0.04)	-0.09 (-0.16, -0.02)	**-0.155 (-0.200, -0.110)**	**<0.001**	30090.0 (23539.2, 37381.1)	380.3 (297.7, 468.7)	-0.22 (-0.41, 0.05)	-0.09 (-0.16, -0.02)	**-3.678 (-4.646, -2.710)**	**<0.001**
South Asia	208641.3 (159471.5, 262090.0)	16.0 (12.2, 20.2)	-0.06 (-0.17, 0.08)	-0.14 (-0.20, -0.07)	-0.025 (-0.085, 0.035)	0.404	4976454.8 (3875592.5, 6171605.3)	336.2 (259.8, 419.2)	-0.08 (-0.20, 0.04)	-0.14 (-0.21, -0.06)	-1.074 (-2.275, 0.127)	0.078
Southeast Asia	20586.6 (17495.2, 24666.0)	3.7 (3.1, 4.4)	-0.28 (-0.38, -0.14)	0.12 (0.04, 0.21)	-0.072 (-0.095, -0.050)	**<0.001**	469958.5 (397494.6, 560466.1)	73.7 (62.5, 88.0)	-0.32 (-0.42, -0.19)	0.11 (0.02, 0.21)	**-1.668 (-2.116, -1.221)**	**<0.001**
Southern Latin America	12873.7 (11609.3, 13708.0)	14.2 (12.8, 15.1)	-0.53 (-0.55, -0.51)	-0.10 (-0.12, -0.09)	**-0.463 (-0.505, -0.420)**	**<0.001**	227253.1 (211025.7, 239927.2)	260.6 (243.0, 274.7)	-0.55 (-0.57, -0.54)	-0.10 (-0.12, -0.08)	**-9.819 (-10.614, -9.024)**	**<0.001**
Southern Sub-Saharan Africa	13149.4 (12185.1, 14108.1)	27.4 (25.2, 29.4)	0.13 (0.05, 0.21)	0.16 (0.12, 0.21)	0.061 (-0.080, 0.202)	0.384	304599.9 (281039.7, 328694.3)	538.9 (499.1, 578.3)	0.09 (0.01, 0.17)	0.18 (0.13, 0.23)	0.693 (-2.190, 3.575)	0.627
Tropical Latin America	16008.9 (13888.0, 17935.4)	6.4 (5.6, 7.2)	-0.52 (-0.54, -0.50)	0.28 (0.24, 0.32)	**-0.258 (-0.289, -0.226)**	**<0.001**	338605.1 (303565.3, 374623.2)	132.4 (118.0, 146.4)	-0.53 (-0.55, -0.51)	0.31 (0.26, 0.35)	**-5.636 (-6.326, -4.947)**	**<0.001**
Western Europe	124973.2 (104731.0, 137461.9)	10.6 (9.0, 11.5)	-0.56 (-0.59, -0.54)	-0.00 (-0.01, 0.00)	**-0.498 (-0.539, -0.457)**	**<0.001**	1710470.5 (1491860.9, 1856595.9)	167.9 (150.4, 180.6)	-0.59 (-0.61, -0.58)	-0.01 (-0.02, -0.00)	**-8.996 (-9.775, -8.218)**	**<0.001**
Western Sub-Saharan Africa	3681.8 (2578.8, 4876.5)	2.2 (1.6, 2.9)	-0.45 (-0.53, -0.33)	-0.28 (-0.34, -0.22)	**-0.078 (-0.106, -0.051)**	**<0.001**	98430.5 (68881.5, 134524.1)	45.5 (31.8, 60.5)	-0.47 (-0.56, -0.34)	-0.28 (-0.35, -0.21)	**-1.778 (-2.362, -1.194)**	**<0.001**
**SDI**												

High SDI	276232.6 (236517.3, 302627.8)	11.5 (10.0, 12.5)	-0.51 (-0.53, -0.49)	-0.02 (-0.04, -0.00)	**-0.454 (-0.485, -0.422)**	**<0.001**	4368372.7 (3926400.9, 4697469.2)	208.0 (190.1, 222.7)	-0.51 (-0.53, -0.50)	-0.03 (-0.05, -0.01)	**-8.229 (-8.839, -7.619)**	**<0.001**
High-middle SDI	459562.2 (407532.3, 505767.6)	23.9 (21.2, 26.3)	-0.45 (-0.49, -0.40)	-0.01 (-0.09, 0.09)	**-0.816 (-0.878, -0.755)**	**<0.001**	7945906.9 (7172611.3, 8731129.8)	411.6 (371.3, 451.9)	-0.48 (-0.52, -0.42)	-0.01 (-0.10, 0.10)	**-16.003 (-17.282, -14.724)**	**<0.001**
Middle SDI	489336.2 (431456.4, 551255.2)	21.1 (18.5, 23.7)	-0.41 (-0.48, -0.32)	-0.01 (-0.13, 0.12)	**-0.541 (-0.584, -0.499)**	**<0.001**	9444612.1 (8428072.6, 10676562.7)	372.0 (330.9, 418.8)	-0.45 (-0.52, -0.38)	-0.01 (-0.14, 0.12)	**-11.348 (-12.165, -10.531)**	**<0.001**
Low-middle SDI	192929.5 (160660.8, 232019.8)	15.2 (12.6, 18.4)	-0.13 (-0.21, -0.05)	-0.11 (-0.16, -0.06)	**-0.079 (-0.120, -0.038)**	**<0.001**	4565128.7 (3828434.5, 5460042.0)	316.9 (264.7, 379.1)	-0.15 (-0.22, -0.06)	-0.11 (-0.17, -0.06)	**-1.975 (-2.807, -1.144)**	**<0.001**
Low SDI	58429.5 (50235.5, 67773.1)	14.1 (12.0, 16.4)	-0.19 (-0.26, -0.11)	-0.08 (-0.13, -0.02)	**-0.127 (-0.173, -0.081)**	**<0.001**	1451870.1 (1263822.3, 1672774.2)	282.8 (243.9, 327.4)	-0.25 (-0.31, -0.18)	-0.07 (-0.13, -0.01)	**-3.862 (-4.735, -2.989)**	**<0.001**

ASMR, age-standardized mortality rates; ASRDALYs, age-standardized rates of DALYs; DALYs, disability-adjusted life-years; SDI = socio-demographic index.

Table S4. Non-communicable disease deaths attributable to high temperature by sex in 2021 at the region and SDI levels and relative change from 1990 to 2021 and 2019 to 2021.

**Categories**	**Female death**						**Male Death**					
**location**	**Number of cases**	**ASMR**	**Relative change in ASMR 1990 - 2021**	**Relative change in ASMR 2019-2021**	**Trends of age-standardized rate from 1990 to 2021 (β)**	***P* value**	**Number of cases**	**ASMR**	**Relative change in ASMR 1990-2021**	**Relative change in ASMR 2019-2021**	**Trends of age-standardized rate from 1990 to 2021 (β)**	***P* value**
**Regions**												
Andean Latin America	31.9 (-4.3, 69.2)	0.1 (-0.0, 0.2)	-2.47 (-32.06, 17.47)	-0.07 (-1.97, 1.38)	**0.007 (0.005, 0.008)**	**<0.001**	**24.6 (-9.8, 61.7)**	0.1 (-0.0, 0.2)	-1.88 (-17.87, 7.29)	-0.04 (-1.97, 2.88)	**0.007 (0.005, 0.008)**	**<0.001**
Australasia	36.1 (7.1, 85.3)	0.1 (0.0, 0.2)	-0.73 (-0.86, -0.48)	-0.54 (-0.69, -0.49)	**-0.005 (-0.007, -0.003)**	**<0.001**	**39.3 (7.4, 96.1)**	0.2 (0.0, 0.4)	-0.74 (-0.83, -0.57)	-0.54 (-0.71, -0.50)	**-0.008 (-0.011, -0.005)**	**<0.001**
Caribbean	217.8 (148.5, 291.9)	0.7 (0.5, 1.0)	0.05 (-4.10, 2.92)	-0.28 (-0.35, -0.16)	-0.008 (-0.086, 0.070)	0.840	182.9 (115.7, 255.2)	0.7 (0.5, 1.0)	0.28 (-4.43, 5.14)	-0.28 (-0.37, -0.15)	-0.001 (-0.066, 0.063)	0.971
Central Asia	1679.8 (717.0, 3538.7)	4.2 (1.7, 8.9)	0.97 (0.55, 2.87)	0.22 (0.14, 0.30)	**0.046 (0.029, 0.064)**	**<0.001**	1805.8 (803.3, 3721.8)	6.3 (2.7, 13.7)	0.89 (0.48, 2.78)	0.21 (0.13, 0.30)	**0.073 (0.046, 0.101)**	**<0.001**
Central Europe	1026.0 (182.7, 2773.2)	0.7 (0.1, 1.8)	0.53 (0.08, 6.79)	0.10 (-0.03, 0.90)	-0.001 (-0.009, 0.007)	0.747	927.4 (189.7, 2460.9)	1.0 (0.2, 2.8)	0.43 (0.04, 5.91)	0.06 (-0.06, 0.69)	-0.003 (-0.015, 0.010)	0.672
Central Latin America	1385.6 (788.4, 2010.5)	1.0 (0.6, 1.5)	3.58 (-29.03, 24.98)	-0.29 (-0.37, -0.22)	**0.043 (0.028, 0.058)**	**<0.001**	1535.9 (785.2, 2253.9)	1.4 (0.7, 2.0)	8.44 (-27.16, 28.59)	-0.27 (-0.36, -0.18)	**0.062 (0.043, 0.082)**	**<0.001**
Central Sub-Saharan Africa	6.5 (-376.2, 232.6)	-0.1 (-1.7, 1.0)	-0.96 (-7.08, 0.43)	-0.78 (-5.22, 4.67)	**0.037 (0.030, 0.044)**	**<0.001**	67.7 (-303.7, 292.6)	0.3 (-1.6, 1.4)	-1.16 (-10.34, 14.91)	5.55 (-2.62, 3.55)	**0.052 (0.044, 0.061)**	**<0.001**
East Asia	18942.9 (5781.3, 42301.7)	1.8 (0.5, 4.0)	-0.32 (-0.57, 0.71)	-0.02 (-0.26, 0.25)	**-0.021 (-0.035, -0.008)**	**0.003**	24970.6 (7418.5, 54187.3)	3.2 (1.0, 6.8)	-0.01 (-0.40, 1.34)	-0.00 (-0.25, 0.26)	0.010 (-0.011, 0.031)	0.344
Eastern Europe	1506.5 (107.0, 4771.1)	0.6 (0.1, 1.9)	2.61 (-28.82, 29.69)	0.28 (0.01, 0.99)	**0.012 (0.004, 0.020)**	**0.004**	1256.5 (184.8, 3682.1)	1.0 (0.2, 3.1)	2.23 (-43.98, 34.18)	0.26 (0.01, 0.93)	**0.018 (0.003, 0.034)**	**0.021**
Eastern Sub-Saharan Africa	894.5 (343.9, 1495.7)	1.1 (0.4, 1.9)	0.50 (-3.80, 3.72)	0.01 (-0.06, 0.23)	**0.014 (0.007, 0.020)**	**<0.001**	1217.4 (547.0, 1950.2)	1.7 (0.8, 2.7)	0.55 (-1.94, 2.99)	-0.03 (-0.08, 0.11)	**0.024 (0.016, 0.033)**	**<0.001**

High-income Asia Pacific	688.6 (137.1, 1693.3)	0.2 (0.0, 0.4)	-0.72 (-0.80, -0.62)	-0.15 (-0.37, -0.09)	**-0.007 (-0.011, -0.002)**	**0.003**	719.9 (158.9, 1771.6)	0.3 (0.1, 0.8)	-0.63 (-0.73, -0.52)	-0.18 (-0.36, -0.11)	-0.007 (-0.014, 0.000)	0.059
High-income North America	3369.1 (1029.1, 7524.2)	0.8 (0.3, 1.8)	-0.20 (-0.50, 0.75)	-0.12 (-0.19, -0.09)	-0.003 (-0.010, 0.004)	0.391	3887.0 (1128.8, 8903.0)	1.3 (0.4, 3.0)	-0.24 (-0.44, 0.50)	-0.11 (-0.18, -0.07)	**-0.008 (-0.018, 0.003)**	**0.148**
North Africa and Middle East	22556.3 (11691.7, 36352.9)	11.8 (6.1, 19.2)	0.15 (-0.02, 0.44)	0.09 (0.05, 0.14)	0.024 (-0.008, 0.056)	0.138	29247.6 (15295.5, 47321.9)	14.4 (7.6, 23.3)	0.24 (0.04, 0.55)	0.10 (0.05, 0.15)	**0.053 (0.015, 0.091)**	**0.007**
Oceania	19.4 (11.4, 25.0)	0.6 (0.3, 0.8)	4.00 (-6.41, 21.21)	0.10 (-0.01, 0.34)	**0.017 (0.013, 0.021)**	**<0.001**	21.5 (10.0, 29.8)	0.6 (0.3, 0.9)	5.71 (-14.08, 18.57)	0.22 (0.06, 0.70)	**0.017 (0.013, 0.022)**	**<0.001**
South Asia	62872.4 (38509.2, 90239.1)	9.3 (5.7, 13.4)	0.19 (0.00, 0.55)	-0.31 (-0.38, -0.22)	**0.103 (0.053, 0.153)**	**<0.001**	79861.1 (47211.1, 118898.4)	12.7 (7.4, 18.9)	0.27 (0.04, 0.72)	-0.28 (-0.36, -0.20)	**0.170 (0.106, 0.235)**	**<0.001**
Southeast Asia	10778.2 (8233.9, 13952.8)	3.5 (2.7, 4.6)	0.51 (0.15, 1.88)	-0.25 (-0.29, -0.20)	**0.052 (0.016, 0.087)**	**0.006**	11467.2 (8438.9, 15062.6)	4.5 (3.3, 5.9)	0.82 (0.32, 3.58)	-0.25 (-0.30, -0.20)	**0.087 (0.043, 0.130)**	**<0.001**
Southern Latin America	127.1 (19.9, 267.7)	0.2 (0.0, 0.5)	-0.56 (-0.76, -0.35)	-0.39 (-0.66, -0.32)	-0.001 (-0.004, 0.002)	0.371	139.3 (25.7, 295.5)	0.4 (0.1, 0.8)	-0.54 (-0.74, -0.37)	-0.39 (-0.66, -0.33)	-0.002 (-0.007, 0.003)	0.502
Southern Sub-Saharan Africa	141.4 (-24.1, 357.4)	0.5 (-0.1, 1.2)	1.04 (-5.57, 6.57)	-0.55 (-1.16, 0.01)	**0.019 (0.010, 0.027)**	**<0.001**	108.6 (-13.0, 260.6)	0.5 (-0.1, 1.2)	0.75 (-5.05, 4.98)	-0.58 (-1.11, -0.52)	**0.017 (0.007, 0.028)**	**0.002**
Tropical Latin America	528.6 (113.2, 1043.2)	0.4 (0.1, 0.7)	-0.06 (-4.86, 4.82)	-0.49 (-0.61, -0.30)	**0.007 (0.000, 0.014)**	**0.038**	475.6 (-49.1, 1071.2)	0.4 (-0.0, 1.0)	0.38 (-11.93, 17.74)	-0.53 (-0.80, -0.26)	**0.015 (0.006, 0.024)**	**0.002**
Western Europe	849.1 (276.4, 1987.9)	0.1 (0.0, 0.3)	-0.31 (-0.56, 0.90)	-0.26 (-0.31, -0.09)	-0.002 (-0.004, 0.000)	0.064	869.1 (268.0, 2254.7)	0.2 (0.1, 0.5)	-0.29 (-0.49, 0.80)	-0.28 (-0.32, -0.15)	-0.003 (-0.006, 0.001)	0.106
Western Sub-Saharan Africa	7678.0 (5436.3, 10082.0)	9.1 (6.4, 11.9)	0.32 (0.04, 1.61)	0.09 (0.01, 0.20)	**0.098 (0.061, 0.134)**	**<0.001**	8304.0 (5765.7, 10997.9)	10.5 (7.4, 13.9)	0.45 (0.13, 2.04)	0.08 (-0.03, 0.24)	**0.134 (0.092, 0.177)**	**<0.001**
**SDI**												
High SDI	8375.1 (3298.7, 16974.0)	0.7 (0.3, 1.3)	-0.00 (-0.31, 0.74)	-0.07 (-0.11, -0.02)	0.002 (-0.001, 0.005)	0.174	11790.4 (5001.8, 22220.4)	1.3 (0.6, 2.5)	0.09 (-0.20, 0.85)	-0.05 (-0.09, -0.01)	**0.007 (0.003, 0.012)**	**0.004**

High-middle SDI	13506.8 (4033.0, 30030.8)	1.2 (0.4, 2.6)	0.16 (-0.20, 1.09)	0.05 (-0.12, 0.22)	0.005 (-0.001, 0.011)	0.087	15713.4 (4779.4, 33890.8)	2.0 (0.6, 4.3)	0.27 (-0.11, 1.15)	0.04 (-0.13, 0.26)	**0.015 (0.005, 0.025)**	**0.004**
Middle SDI	42217.2 (24187.8, 64268.2)	3.2 (1.8, 4.9)	0.15 (-0.11, 0.72)	-0.16 (-0.23, -0.10)	**0.023 (0.010, 0.036)**	**<0.001**	52888.9 (29536.6, 82966.1)	4.8 (2.7, 7.6)	0.38 (0.08, 0.97)	-0.14 (-0.22, -0.06)	**0.056 (0.039, 0.073)**	**<0.001**
Low-middle SDI	58075.6 (36295.1, 82751.1)	8.6 (5.3, 12.3)	0.26 (0.07, 0.65)	-0.23 (-0.29, -0.16)	**0.100 (0.062, 0.138)**	**<0.001**	71181.5 (42858.1, 105332.4)	11.8 (7.0, 17.6)	0.36 (0.15, 0.86)	-0.21 (-0.28, -0.13)	**0.161 (0.112, 0.210)**	**<0.001**
Low SDI	13100.1 (7829.6, 18729.7)	6.0 (3.6, 8.7)	0.21 (-0.02, 1.30)	-0.25 (-0.32, -0.17)	**0.078 (0.049, 0.108)**	**<0.001**	15488.9 (9000.0, 22333.2)	7.6 (4.4, 11.0)	0.32 (0.07, 1.43)	-0.26 (-0.33, -0.18)	**0.118 (0.082, 0.153)**	**<0.001**

ASMR, age-standardized mortality rates; SDI, socio-demographic index.

Table S5. Non-communicable diseases DALYs attributable to high temperature by sex at the region and SDI levels in 2021 and relative change from 1990 to 2021 and 2019 to 2021.

**Categories**	**Female DALYs**						**Male DALYs**					
**location**	**Number of cases**	**ASRDALYs**	**Relative change in ASRDALYs 1990-2021**	**Relative change in SRDALYs 2019-2021**	**Trends of age-standardized rate from 1990 to 2021 (β)**	***P* value**	**Number of cases**	**ASRDALYs**	**Relative change in ASRDALYs 1990-2021**	**Relative change in ASRDALYs 2019-2021**	**Trends of age-standardized rate from 1990 to 2021 (β)**	***P* value**
**Regions**												
Andean Latin America	755.3 (-16.0, 1563.9)	2.4 (-0.1, 5.0)	-3.42 (-29.68, 40.52)	-0.08 (-1.15, 0.68)	**0.132 (0.107, 0.156)**	**<0.001**	634.1 (-139.2, 1477.7)	2.2 (-0.5, 5.1)	-2.21 (-27.53, 14.82)	-0.05 (-2.03, 3.88)	**0.144 (0.116, 0.172)**	**<0.001**
Australasia	470.6 (97.4, 1091.7)	1.5 (0.3, 3.4)	-0.75 (-0.86, -0.51)	-0.54 (-0.68, -0.49)	**-0.081 (-0.110, -0.052)**	**<0.001**	647.2 (125.7, 1583.2)	2.7 (0.5, 6.5)	-0.75 (-0.84, -0.58)	-0.55 (-0.71, -0.50)	**-0.147 (-0.200, -0.093)**	**<0.001**
Caribbean	4321.7 (2891.1, 5844.4)	15.1 (10.0, 20.5)	0.06 (-3.56, 3.37)	-0.29 (-0.37, -0.16)	-0.247 (-2.376, 1.883)	0.814	3856.3 (2298.5, 5485.1)	15.3 (9.1, 21.8)	0.32 (-5.44, 4.05)	-0.30 (-0.40, -0.15)	-0.061 (-1.717, 1.595)	0.940
Central Asia	34307.0 (15873.5, 67223.6)	78.1 (35.4, 156.8)	0.96 (0.54, 2.59)	0.22 (0.13, 0.31)	**0.830 (0.476, 1.184)**	**<0.001**	45687.1 (21414.8, 89696.6)	129.6 (58.4, 264.4)	0.87 (0.43, 2.68)	0.21 (0.12, 0.30)	**1.391 (0.769, 2.014)**	**<0.001**
Central Europe	14640.5 (2754.1, 38763.5)	10.4 (2.0, 27.3)	0.43 (-0.01, 7.09)	0.12 (-0.02, 0.90)	-0.052 (-0.184, 0.081)	0.431	17737.7 (3752.6, 46463.4)	19.5 (4.2, 51.1)	0.38 (-0.00, 5.68)	0.06 (-0.07, 0.66)	-0.092 (-0.334, 0.150)	0.443
Central Latin America	29403.3 (16583.5, 43442.0)	21.7 (12.3, 32.0)	2.85 (-16.93, 19.54)	-0.27 (-0.35, -0.19)	**0.826 (0.540, 1.112)**	**<0.001**	36804.8 (18634.0, 54650.0)	31.4 (15.9, 46.5)	6.67 (-24.47, 31.13)	-0.25 (-0.35, -0.15)	**1.343 (0.928, 1.758)**	**<0.001**
Central Sub-Saharan Africa	744.7 (-8608.9, 6313.0)	0.7 (-31.8, 19.9)	-1.02 (-9.14, 4.72)	-1.21 (-6.50, 2.30)	**0.792 (0.644, 0.939)**	**<0.001**	2334.8 (-8629.1, 9049.5)	6.9 (-33.4, 31.1)	-1.19 (-14.99, 12.32)	2.27 (-3.48, 2.66)	**1.187 (0.999, 1.376)**	**<0.001**
East Asia	307159.2 (93217.0, 684526.3)	28.3 (8.7, 62.9)	-0.38 (-0.60, 0.33)	-0.02 (-0.27, 0.28)	**-0.483 (-0.699, -0.268)**	**<0.001**	472762.0 (140160.1, 1026290.2)	52.2 (15.8, 112.6)	-0.07 (-0.43, 1.04)	-0.00 (-0.27, 0.30)	0.030 (-0.298, 0.358)	0.853
Eastern Europe	23406.3 (2489.4, 70793.6)	10.2 (1.2, 29.4)	3.09 (-52.09, 24.97)	0.34 (0.06, 1.14)	0.208 (0.068, 0.348)	0.005	28698.3 (4731.5, 80957.9)	22.3 (3.6, 63.2)	2.63 (-42.61, 77.87)	0.32 (-0.00, 1.03)	**0.406 (0.063, 0.749)**	**0.022**
Eastern Sub-Saharan Africa	24460.5 (10056.4, 40411.2)	24.8 (9.8, 41.4)	0.37 (-3.76, 2.87)	0.01 (-0.07, 0.18)	**0.232 (0.091, 0.374)**	**0.002**	36167.3 (15594.6, 57410.4)	38.6 (17.4, 61.8)	0.48 (-1.95, 2.69)	-0.01 (-0.07, 0.12)	**0.493 (0.291, 0.694)**	**<0.001**
High-income Asia Pacific	8432.1 (1773.5, 20282.7)	2.8 (0.6, 6.5)	-0.73 (-0.80, -0.63)	-0.15 (-0.34, -0.07)	**-0.125 (-0.198, -0.051)**	**0.002**	12374.3 (2865.9, 30741.8)	6.5 (1.6, 16.2)	-0.62 (-0.71, -0.50)	-0.17 (-0.33, -0.11)	-0.133 (-0.266, 0.000)	0.050

High-income North America	53540.1 (17417.7, 113289.1)	15.1 (5.2, 31.2)	-0.19 (-0.48, 0.56)	-0.14 (-0.21, -0.10)	-0.031 (-0.158, 0.096)	0.623	76033.9 (22022.4, 170888.0)	27.1 (8.0, 59.8)	-0.24 (-0.44, 0.46)	-0.13 (-0.20, -0.09)	-0.133 (-0.348, 0.083)	0.218
North Africa and Middle East	530184.8 (281675.6, 860799.3)	236.7 (123.7, 379.3)	0.10 (-0.08, 0.42)	0.08 (0.03, 0.14)	0.301 (-0.344, 0.946)	0.348	761185.1 (395703.2, 1237214.9)	312.6 (163.7, 504.8)	0.25 (0.04, 0.59)	0.09 (0.03, 0.14)	**1.385 (0.581, 2.189)**	**0.001**
Oceania	533.6 (302.2, 699.3)	13.9 (8.1, 18.0)	3.84 (-5.92, 12.39)	0.13 (0.02, 0.37)	**0.377 (0.289, 0.466)**	**<0.001**	616.1 (284.1, 860.4)	14.6 (6.7, 20.2)	5.69 (-11.53, 11.09)	0.23 (0.07, 0.67)	**0.396 (0.297, 0.496)**	**<0.001**
South Asia	1451436.2 (891567.6, 2087360.8)	193.4 (119.9, 276.9)	0.14 (-0.04, 0.50)	-0.30 (-0.37, -0.21)	**1.758 (0.753, 2.762)**	**0.001**	1982220.2 (1187719.4, 2968360.6)	271.1 (162.0, 407.6)	0.24 (0.01, 0.66)	-0.29 (-0.37, -0.19)	**3.189 (1.829, 4.548)**	**<0.001**
Southeast Asia	225199.5 (177270.9, 289400.1)	66.8 (52.2, 86.1)	0.38 (0.04, 1.71)	-0.25 (-0.30, -0.20)	**0.781 (0.076, 1.486)**	**0.031**	285610.1 (211187.1, 375358.7)	93.9 (69.2, 123.1)	0.76 (0.27, 3.50)	-0.26 (-0.31, -0.20)	**1.746 (0.818, 2.674)**	**<0.001**
Southern Latin America	2106.9 (372.6, 4320.2)	4.3 (0.8, 8.7)	-0.57 (-0.75, -0.39)	-0.38 (-0.63, -0.31)	-0.037 (-0.092, 0.018)	0.175	2942.6 (575.6, 6216.7)	7.8 (1.5, 16.5)	-0.55 (-0.74, -0.40)	-0.38 (-0.63, -0.32)	-0.061 (-0.162, 0.040)	0.227
Southern Sub-Saharan Africa	3285.0 (-410.4, 7913.5)	9.8 (-1.4, 24.1)	0.96 (-5.49, 9.67)	-0.53 (-1.25, -0.36)	**0.364 (0.193, 0.536)**	**<0.001**	3010.2 (-289.8, 7062.2)	11.5 (-1.3, 27.6)	0.77 (-7.44, 6.49)	-0.56 (-1.02, -0.50)	**0.360 (0.140, 0.579)**	**0.002**
Tropical Latin America	10785.8 (2185.0, 20773.8)	7.7 (1.5, 14.7)	-0.05 (-5.27, 4.73)	-0.48 (-0.60, -0.30)	**0.143 (0.003, 0.283)**	**0.045**	10523.4 (-2065.5, 24282.8)	9.2 (-1.6, 21.0)	0.43 (-14.18, 7.35)	-0.54 (-0.87, 0.09)	**0.308 (0.117, 0.499)**	**0.003**
Western Europe	10203.1 (3438.3, 24285.2)	1.6 (0.5, 3.7)	-0.37 (-0.59, 0.64)	-0.27 (-0.32, -0.08)	**-0.034 (-0.063, -0.005)**	**0.022**	13926.0 (4074.3, 36708.1)	3.4 (1.0, 9.0)	-0.34 (-0.52, 0.70)	-0.28 (-0.32, -0.15)	**-0.061 (-0.121, -0.001)**	**0.047**
Western Sub-Saharan Africa	192338.5 (134817.9, 257590.1)	178.6 (126.6, 234.4)	0.22 (-0.05, 1.41)	0.08 (-0.01, 0.21)	**1.558 (0.819, 2.297)**	**<0.001**	226174.3 (152953.5, 304449.8)	219.4 (152.3, 290.6)	0.38 (0.05, 1.96)	0.08 (-0.04, 0.23)	**2.624 (1.728, 3.520)**	**<0.001**
**SDI**												
High SDI	167010.2 (74998.3, 302947.4)	18.2 (8.9, 31.0)	0.26 (-0.11, 1.02)	-0.03 (-0.08, 0.04)	**0.152 (0.093, 0.211)**	**<0.001**	292205.0 (132800.8, 515088.0)	36.6 (16.8, 62.8)	0.38 (-0.00, 1.29)	-0.02 (-0.07, 0.04)	**0.414 (0.309, 0.519)**	**<0.001**

High-middle SDI	221113.9 (72650.1, 467330.7)	20.4 (7.0, 42.6)	0.08 (-0.24, 0.92)	0.03 (-0.13, 0.21)	0.043 (-0.060, 0.147)	0.400	315751.8 (102723.2, 678472.6)	37.7 (12.3, 80.8)	0.21 (-0.15, 0.99)	0.04 (-0.14, 0.25)	**0.222 (0.044, 0.400)**	**0.016**
Middle SDI	864946.2 (509534.5, 1305368.0)	62.8 (37.0, 94.8)	0.12 (-0.13, 0.64)	-0.17 (-0.24, -0.10)	**0.371 (0.123, 0.619)**	**0.005**	1221158.7 (687756.3, 1876938.0)	98.2 (55.3, 152.2)	0.35 (0.06, 0.91)	-0.16 (-0.23, -0.08)	**1.059 (0.706, 1.411)**	**<0.001**
Low-middle SDI	1345221.2 (836470.0, 1917009.3)	179.4 (112.0, 255.1)	0.20 (0.02, 0.55)	-0.22 (-0.29, -0.14)	**1.724 (0.940, 2.508)**	**<0.001**	1779579.7 (1081636.1, 2602258.2)	253.3 (152.8, 371.4)	0.33 (0.12, 0.83)	-0.21 (-0.29, -0.13)	**3.190 (2.143, 4.237)**	**<0.001**
Low SDI	328197.8 (194905.6, 465307.0)	122.5 (73.3, 174.1)	0.15 (-0.05, 1.15)	-0.24 (-0.31, -0.16)	**1.223 (0.675, 1.771)**	**<0.001**	409678.2 (245473.5, 582454.0)	156.6 (91.6, 225.5)	0.25 (0.01, 1.34)	-0.24 (-0.33, -0.16)	**1.948 (1.253, 2.643)**	**<0.001**

ASRDALYs, age-standardized rates of DALYs; DALYs, disability-adjusted life-years; SDI, socio-demographic index.

Table S6. Non-communicable disease deaths attributable to low temperature by sex at the region and SDI levels in 2021 and relative change from 1990 to 2021 and 2019 to 2021.

Categories	Female deaths						Male deaths					
**location**	**Number of cases**	**ASMR**	**Relative change in ASMR 1990 - 2021**	**Relative change in ASMR 2019-2021**	**Trends of age-standardized rate from 1990 to 2021 (β)**	***P* value**	**Number of cases**	**ASMR**	**Relative change in ASMR 1990 -2021**	**Relative change in ASMR 2019-2021**	**Trends of age-standardized rate from 1990 to 2021 (β)**	***P* value**
**Regions**												
Andean Latin America	2905.3 (2473.2, 3479.2)	9.5 (8.1, 11.4)	-0.34 (-0.44, -0.21)	-0.03 (-0.12, 0.06)	**-0.195 (-0.223, -0.167)**	**<0.001**	2954.4 (2464.3, 3595.5)	11.2 (9.3, 13.6)	-0.31 (-0.43, -0.16)	-0.01 (-0.12, 0.10)	**-0.164 (-0.196, -0.133)**	**<0.001**
Australasia	2476.4 (1956.4, 2799.5)	6.8 (5.5, 7.6)	-0.61 (-0.64, -0.59)	0.01 (-0.01, 0.03)	**-0.373 (-0.404, -0.343)**	**<0.001**	2693.4 (2414.0, 2957.4)	10.4 (9.3, 11.4)	-0.65 (-0.67, -0.63)	0.03 (0.01, 0.04)	**-0.675 (-0.739, -0.611)**	**<0.001**
Caribbean	496.5 (417.2, 596.0)	1.7 (1.4, 2.0)	-0.41 (-0.49, -0.31)	0.16 (0.06, 0.27)	**-0.052 (-0.071, -0.033)**	**<0.001**	564.7 (472.3, 664.8)	2.3 (1.9, 2.7)	-0.33 (-0.42, -0.21)	0.17 (0.05, 0.30)	**-0.054 (-0.078, -0.031)**	**<0.001**
Central Asia	13115.6 (11692.6, 14699.4)	32.8 (29.3, 36.7)	-0.17 (-0.24, -0.10)	-0.01 (-0.07, 0.06)	**-0.468 (-0.584, -0.352)**	**<0.001**	13626.0 (12276.0, 15331.0)	49.0 (44.2, 54.9)	-0.18 (-0.25, -0.11)	-0.02 (-0.08, 0.05)	**-0.627 (-0.802, -0.451)**	**<0.001**
Central Europe	32897.8 (28962.3, 36074.3)	21.4 (19.0, 23.4)	-0.44 (-0.48, -0.41)	0.12 (0.07, 0.18)	**-0.777 (-0.844, -0.711)**	**<0.001**	28830.9 (26582.9, 31295.2)	32.2 (29.6, 34.9)	-0.44 (-0.47, -0.40)	0.10 (0.04, 0.16)	**-1.093 (-1.189, -0.997)**	**<0.001**
Central Latin America	13352.6 (11696.9, 15336.9)	10.1 (8.8, 11.6)	-0.36 (-0.43, -0.28)	0.07 (-0.03, 0.18)	**-0.205 (-0.229, -0.181)**	**<0.001**	14592.5 (12707.9, 16740.1)	13.5 (11.8, 15.5)	-0.18 (-0.28, -0.08)	0.10 (-0.02, 0.22)	**-0.133 (-0.158, -0.107)**	**<0.001**
Central Sub-Saharan Africa	1460.5 (1119.1, 1854.2)	6.5 (5.1, 8.3)	-0.24 (-0.42, 0.01)	0.05 (-0.01, 0.15)	**-0.102 (-0.128, -0.075)**	**<0.001**	1382.3 (1057.5, 1690.8)	7.3 (5.8, 8.8)	-0.27 (-0.43, -0.03)	0.06 (-0.00, 0.15)	**-0.131 (-0.159, -0.102)**	**<0.001**
East Asia	244869.0 (194576.8, 302448.9)	22.8 (18.1, 28.2)	-0.56 (-0.65, -0.44)	-0.02 (-0.23, 0.27)	**-1.076 (-1.139, -1.013)**	**<0.001**	319667.9 (258733.0, 386660.6)	40.2 (32.9, 47.8)	-0.43 (-0.55, -0.30)	-0.01 (-0.24, 0.26)	**-1.096 (-1.188, -1.003)**	**<0.001**
Eastern Europe	65152.1 (56086.6, 73342.9)	26.3 (22.7, 29.6)	-0.34 (-0.42, -0.26)	0.04 (-0.06, 0.17)	**-0.802 (-0.962, -0.641)**	**<0.001**	50025.7 (44084.5, 56894.5)	41.6 (36.7, 47.1)	-0.33 (-0.41, -0.25)	-0.01 (-0.13, 0.11)	**-1.236 (-1.518, -0.953)**	**<0.001**
Eastern Sub-Saharan Africa	8072.0 (7016.6, 9441.5)	11.4 (9.8, 13.3)	-0.32 (-0.41, -0.20)	0.10 (0.01, 0.20)	**-0.231 (-0.262, -0.200)**	**<0.001**	8930.2 (7843.9, 10122.4)	13.7 (12.0, 15.5)	-0.29 (-0.38, -0.16)	0.09 (-0.00, 0.19)	**-0.252 (-0.287, -0.217)**	**<0.001**

High-income Asia Pacific	20255.7 (14248.4, 23878.5)	4.6 (3.5, 5.3)	-0.71 (-0.75, -0.69)	-0.04 (-0.05, -0.03)	**-0.374 (-0.415, -0.333)**	**<0.001**	21374.7 (18897.1, 22991.6)	9.3 (8.3, 9.9)	-0.61 (-0.63, -0.60)	-0.04 (-0.05, -0.03)	**-0.497 (-0.541, -0.453)**	**<0.001**
High-income North America	48598.3 (40028.3, 53911.8)	11.6 (9.8, 12.7)	-0.32 (-0.36, -0.29)	-0.06 (-0.07, -0.04)	**-0.245 (-0.277, -0.212)**	**<0.001**	50238.4 (45015.7, 53885.4)	17.1 (15.3, 18.3)	-0.40 (-0.43, -0.38)	-0.06 (-0.08, -0.04)	**-0.475 (-0.521, -0.428)**	**<0.001**
North Africa and Middle East	49923.1 (43980.8, 56460.2)	28.2 (24.6, 31.6)	-0.32 (-0.38, -0.26)	-0.09 (-0.12, -0.05)	**-0.432 (-0.523, -0.341)**	**<0.001**	58276.3 (51785.4, 66575.1)	30.7 (27.1, 34.7)	-0.35 (-0.40, -0.29)	-0.08 (-0.12, -0.05)	**-0.622 (-0.712, -0.532)**	**<0.001**
Oceania	479.8 (371.0, 592.6)	16.0 (12.5, 19.7)	-0.20 (-0.38, 0.05)	-0.08 (-0.15, -0.02)	**-0.153 (-0.197, -0.109)**	**<0.001**	602.9 (471.9, 748.2)	19.1 (15.2, 23.6)	-0.20 (-0.39, 0.04)	-0.09 (-0.18, -0.02)	**-0.162 (-0.210, -0.114)**	**<0.001**
South Asia	89973.3 (68817.9, 112914.5)	13.4 (10.1, 16.8)	-0.09 (-0.22, 0.08)	-0.15 (-0.23, -0.06)	-0.041 (-0.095, 0.014)	0.139	118668.0 (89002.2, 152431.2)	18.9 (14.2, 24.4)	-0.00 (-0.14, 0.20)	-0.12 (-0.22, -0.02)	0.009 (-0.059, 0.077)	0.791
Southeast Asia	9422.9 (7826.0, 11349.3)	3.1 (2.6, 3.8)	-0.34 (-0.45, -0.19)	0.11 (0.03, 0.21)	**-0.077 (-0.097, -0.058)**	**<0.001**	11163.7 (9413.9, 13256.4)	4.4 (3.7, 5.2)	-0.21 (-0.33, -0.05)	0.12 (0.02, 0.25)	**-0.065 (-0.090, -0.039)**	**<0.001**
Southern Latin America	6313.4 (5499.4, 6819.6)	11.3 (10.0, 12.2)	-0.53 (-0.55, -0.51)	-0.10 (-0.12, -0.08)	**-0.361 (-0.397, -0.325)**	**<0.001**	6560.3 (6141.4, 6920.5)	17.9 (16.7, 18.9)	-0.53 (-0.54, -0.51)	-0.11 (-0.13, -0.09)	**-0.586 (-0.638, -0.535)**	**<0.001**
Southern Sub-Saharan Africa	7234.4 (6660.1, 7867.8)	25.2 (23.0, 27.3)	0.17 (0.08, 0.28)	0.17 (0.12, 0.23)	0.111 (-0.010, 0.233)	0.072	5914.9 (5450.4, 6413.3)	30.3 (27.9, 32.7)	0.09 (-0.00, 0.19)	0.14 (0.08, 0.21)	-0.012 (-0.182, 0.159)	0.891
Tropical Latin America	7596.0 (6324.6, 8611.8)	5.3 (4.4, 6.0)	-0.53 (-0.56, -0.50)	0.30 (0.24, 0.34)	**-0.223 (-0.250, -0.196)**	**<0.001**	8412.9 (7491.4, 9318.1)	7.8 (6.9, 8.7)	-0.50 (-0.52, -0.47)	0.28 (0.22, 0.33)	**-0.293 (-0.330, -0.256)**	**<0.001**
Western Europe	64747.0 (50535.2, 73024.9)	8.5 (6.8, 9.4)	-0.56 (-0.59, -0.54)	-0.00 (-0.02, 0.01)	**-0.391 (-0.423, -0.359)**	**<0.001**	60226.2 (53761.4, 64820.5)	13.3 (11.9, 14.2)	-0.58 (-0.60, -0.57)	-0.01 (-0.02, 0.00)	**-0.683 (-0.740, -0.626)**	**<0.001**
Western Sub-Saharan Africa	1712.1 (1207.6, 2295.4)	2.0 (1.5, 2.7)	-0.50 (-0.59, -0.38)	-0.29 (-0.35, -0.20)	**-0.082 (-0.107, -0.056)**	**<0.001**	1969.8 (1346.0, 2677.5)	2.5 (1.7, 3.3)	-0.40 (-0.51, -0.22)	-0.28 (-0.38, -0.16)	**-0.073 (-0.102, -0.044)**	**<0.001**
**SDI**												

High SDI	136755.0 (110132.1, 153207.5)	9.1 (7.6, 10.1)	-0.52 (-0.54, -0.50)	-0.02 (-0.04, 0.00)	**-0.370 (-0.394, -0.346)**	**<0.001**	139477.6 (126153.9, 149571.4)	14.4 (13.0, 15.4)	-0.52 (-0.55, -0.51)	-0.02 (-0.05, 0.00)	**-0.601 (-0.645, -0.557)**	**<0.001**
High-middle SDI	227088.9 (190312.4, 255665.8)	19.6 (16.4, 22.0)	-0.47 (-0.52, -0.41)	0.00 (-0.10, 0.13)	**-0.740 (-0.792, -0.687)**	**<0.001**	232473.3 (205336.9, 261476.2)	29.9 (26.4, 33.5)	-0.43 (-0.50, -0.36)	-0.01 (-0.15, 0.13)	**-0.940 (-1.016, -0.863)**	**<0.001**
Middle SDI	212671.8 (178974.3, 245818.1)	16.6 (13.9, 19.2)	-0.49 (-0.57, -0.39)	-0.01 (-0.15, 0.17)	**-0.583 (-0.628, -0.539)**	**<0.001**	276664.4 (236916.3, 321827.5)	26.9 (23.2, 31.1)	-0.31 (-0.43, -0.19)	-0.00 (-0.18, 0.20)	**-0.474 (-0.520, -0.428)**	**<0.001**
Low-middle SDI	86743.0 (70868.9, 104427.2)	13.1 (10.7, 15.8)	-0.20 (-0.28, -0.09)	-0.12 (-0.18, -0.06)	**-0.105 (-0.141, -0.069)**	**<0.001**	106186.5 (85233.2, 130794.4)	17.7 (14.1, 21.7)	-0.06 (-0.16, 0.06)	-0.10 (-0.17, -0.03)	-0.042 (-0.090, 0.006)	0.084
Low SDI	27153.1 (22987.1, 31510.5)	12.8 (10.8, 14.9)	-0.20 (-0.29, -0.09)	-0.08 (-0.14, -0.01)	**-0.129 (-0.173, -0.085)**	**<0.001**	31276.4 (26530.0, 36845.9)	15.6 (13.2, 18.4)	-0.17 (-0.26, -0.06)	-0.08 (-0.16, 0.01)	**-0.120 (-0.168, -0.072)**	**<0.001**

ASMR, age-standardized mortality rates; SDI, socio-demographic index.

Table S7. Non-communicable diseases DALYs attributable to low temperature by sex at the region and SDI levels in 2021 and relative change from 1990 to 2021 and 2019 to 2021.

Categories	Female DALYs						Male DALYs					
**Location**	**Number of cases**	**ASRDALYs**	**Relative change in ASRDALYs 1990-2021**	**Relative change in ASRDALYs 2019-2021**	**Trends of age-standardized rate from 1990 to 2021 (β)**	***P* value**	**Number of cases**	**ASRDALYs**	**Relative change in ASRDALYs 1990-2021**	**Relative change in ASRDALYs 2019-2021**	**Trends of age-standardized rate from 1990 to 2021 (β)**	***P* value**
**Regions**												
Andean Latin America	57374.8 (47872.2, 69381.5)	184.3 (154.1, 222.8)	-0.38 (-0.48, -0.25)	-0.02 (-0.12, 0.08)	**-4.406 (-4.971, -3.841)**	**<0.001**	64725.9 (52969.3, 79227.1)	227.5 (186.2, 277.6)	-0.35 (-0.47, -0.20)	-0.00 (-0.12, 0.12)	**-4.040 (-4.716, -3.364)**	**<0.001**
Australasia	32715.5 (27197.8, 36489.0)	102.7 (87.8, 113.5)	-0.63 (-0.66, -0.61)	0.01 (-0.01, 0.03)	**-6.251 (-6.829, -5.673)**	**<0.001**	43523.5 (39862.5, 47360.2)	177.0 (162.6, 193.2)	-0.67 (-0.68, -0.65)	0.02 (0.01, 0.04)	**-12.470 (-13.744, -11.197)**	**<0.001**
Caribbean	9616.9 (7965.1, 11571.5)	33.7 (27.8, 40.7)	-0.39 (-0.48, -0.27)	0.15 (0.05, 0.26)	**-0.989 (-1.352, -0.625)**	**<0.001**	12492.3 (10407.4, 14865.8)	49.8 (41.5, 59.1)	-0.29 (-0.39, -0.16)	0.15 (0.03, 0.29)	**-1.009 (-1.472, -0.545)**	**<0.001**
Central Asia	253880.0 (226720.3, 286216.2)	587.6 (524.5, 660.8)	-0.21 (-0.28, -0.13)	-0.00 (-0.08, 0.07)	**-10.675 (-13.214, -8.136)**	**<0.001**	331692.4 (297691.3, 375635.2)	966.5 (870.1, 1087.8)	-0.21 (-0.29, -0.13)	-0.02 (-0.09, 0.05)	**-15.929 (-20.178, -11.681)**	**<0.001**
Central Europe	467103.5 (417089.6, 510995.2)	333.0 (299.5, 364.3)	-0.49 (-0.52, -0.45)	0.13 (0.06, 0.19)	**-14.064 (-15.229, -12.899)**	**<0.001**	545434.9 (502124.3, 593486.9)	599.0 (551.8, 651.5)	-0.46 (-0.49, -0.42)	0.10 (0.03, 0.17)	**-22.281 (-24.226, -20.336)**	**<0.001**
Central Latin America	258220.0 (225361.6, 302938.6)	191.7 (167.6, 224.7)	-0.34 (-0.42, -0.25)	0.09 (-0.03, 0.22)	**-3.814 (-4.228, -3.400)**	**<0.001**	326151.5 (281589.2, 375677.6)	282.2 (244.5, 324.6)	-0.15 (-0.27, -0.04)	0.10 (-0.03, 0.24)	**-2.466 (-2.989, -1.942)**	**<0.001**
Central Sub-Saharan Africa	35105.6 (26204.6, 44992.4)	123.9 (94.8, 157.5)	-0.28 (-0.47, -0.03)	0.05 (-0.02, 0.15)	**-2.265 (-2.801, -1.730)**	**<0.001**	39514.6 (29816.4, 49112.2)	151.5 (117.2, 184.4)	-0.30 (-0.46, -0.06)	0.06 (-0.01, 0.15)	**-3.076 (-3.698, -2.454)**	**<0.001**
East Asia	4004821.7 (3207250.2, 4964416.6)	363.0 (290.7, 449.8)	-0.60 (-0.69, -0.48)	-0.02 (-0.25, 0.29)	**-19.999 (-21.120, -18.878)**	**<0.001**	5933300.3 (4733127.3, 7297255.0)	647.7 (523.7, 786.2)	-0.47 (-0.59, -0.32)	-0.02 (-0.27, 0.29)	**-20.090 (-21.463, -18.716)**	**<0.001**
Eastern Europe	1004897.8 (870786.0, 1131794.3)	439.6 (382.4, 494.2)	-0.34 (-0.42, -0.25)	0.04 (-0.07, 0.19)	**-13.820 (-16.882, -10.758)**	**<0.001**	1129730.3 (994133.6, 1284909.4)	878.3 (774.6, 998.4)	-0.28 (-0.37, -0.19)	-0.01 (-0.13, 0.12)	**-23.520 (-30.681, -16.360)**	**<0.001**
Eastern Sub-Saharan Africa	194311.0 (169619.2, 228285.5)	217.8 (190.3, 254.8)	-0.39 (-0.47, -0.27)	0.10 (0.01, 0.21)	**-5.728 (-6.389, -5.067)**	**<0.001**	243265.8 (212809.8, 276246.3)	282.8 (248.4, 320.9)	-0.34 (-0.44, -0.23)	0.10 (0.00, 0.20)	**-6.433 (-7.210, -5.655)**	**<0.001**

High-income Asia Pacific	235057.1 (175187.1, 272090.1)	70.7 (57.2, 79.4)	-0.73 (-0.75, -0.71)	-0.04 (-0.05, -0.03)	**-6.302 (-6.935, -5.669)**	**<0.001**	332822.7 (302798.5, 354903.9)	165.1 (152.1, 175.5)	-0.61 (-0.63, -0.60)	-0.04 (-0.05, -0.03)	**-9.074 (-9.769, -8.378)**	**<0.001**
High-income North America	752922.7 (654013.4, 812967.1)	206.6 (184.0, 220.9)	-0.32 (-0.36, -0.30)	-0.06 (-0.08, -0.04)	**-4.298 (-4.841, -3.754)**	**<0.001**	949572.2 (877539.0, 1012238.6)	333.6 (309.2, 355.2)	-0.40 (-0.43, -0.38)	-0.07 (-0.08, -0.05)	**-9.136 (-10.051, -8.221)**	**<0.001**
North Africa and Middle East	1025099.5 (905093.6, 1172536.8)	492.3 (434.4, 557.7)	-0.38 (-0.44, -0.32)	-0.09 (-0.13, -0.06)	**-10.684 (-12.242, -9.127)**	**<0.001**	1358318.5 (1193524.4, 1585209.0)	597.3 (529.8, 687.8)	-0.39 (-0.44, -0.33)	-0.09 (-0.13, -0.05)	**-14.293 (-16.182, -12.403)**	**<0.001**
Oceania	13220.9 (10208.6, 16450.1)	346.0 (268.0, 427.5)	-0.22 (-0.41, 0.04)	-0.08 (-0.15, -0.02)	**-3.654 (-4.559, -2.749)**	**<0.001**	16869.1 (13145.1, 21130.0)	413.5 (324.8, 513.2)	-0.21 (-0.42, 0.05)	-0.09 (-0.18, -0.02)	**-3.746 (-4.831, -2.660)**	**<0.001**
South Asia	2047599.0 (1598091.6, 2561837.3)	273.9 (213.0, 342.4)	-0.13 (-0.25, 0.04)	-0.15 (-0.24, -0.04)	**-1.332 (-2.400, -0.264)**	**0.016**	2928855.8 (2230851.8, 3735003.7)	401.7 (304.1, 514.9)	-0.03 (-0.18, 0.15)	-0.12 (-0.24, -0.01)	-0.517 (-1.891, 0.858)	0.449
Southeast Asia	194736.4 (162086.3, 237116.3)	58.1 (48.3, 70.6)	-0.40 (-0.50, -0.27)	0.09 (-0.01, 0.21)	**-1.784 (-2.163, -1.405)**	**<0.001**	275222.1 (232693.5, 327004.9)	91.2 (77.0, 108.4)	-0.24 (-0.36, -0.09)	0.12 (0.00, 0.26)	**-1.527 (-2.055, -0.999)**	**<0.001**
Southern Latin America	98324.4 (88949.5, 104868.4)	193.2 (176.7, 204.9)	-0.55 (-0.57, -0.54)	-0.09 (-0.11, -0.08)	**-7.014 (-7.661, -6.367)**	**<0.001**	128928.7 (122916.2, 135752.0)	342.7 (326.0, 360.7)	-0.55 (-0.57, -0.54)	-0.10 (-0.12, -0.08)	**-13.166 (-14.156, -12.175)**	**<0.001**
Southern Sub-Saharan Africa	152874.5 (140873.5, 167306.3)	474.1 (437.4, 518.0)	0.11 (0.01, 0.22)	0.19 (0.12, 0.26)	1.885 (-0.414, 4.183)	0.104	151725.4 (138672.0, 166230.9)	622.0 (573.3, 675.4)	0.07 (-0.03, 0.18)	0.17 (0.09, 0.25)	-0.827 (-4.612, 2.957)	0.658
Tropical Latin America	147513.4 (128193.9, 164710.9)	104.4 (90.9, 116.4)	-0.54 (-0.56, -0.51)	0.33 (0.27, 0.38)	**-4.621 (-5.183, -4.059)**	**<0.001**	191091.8 (173231.4, 210875.2)	165.6 (150.2, 182.7)	-0.52 (-0.54, -0.49)	0.30 (0.23, 0.35)	**-6.711 (-7.545, -5.877)**	**<0.001**
Western Europe	785962.2 (641494.0, 875922.8)	123.1 (104.9, 134.9)	-0.59 (-0.62, -0.57)	-0.01 (-0.02, 0.00)	**-6.420 (-6.984, -5.855)**	**<0.001**	924508.3 (848824.5, 985415.2)	220.0 (204.0, 233.2)	-0.61 (-0.62, -0.60)	-0.01 (-0.02, -0.00)	**-12.695 (-13.790, -11.599)**	**<0.001**
Western Sub-Saharan Africa	43341.4 (29811.2, 59247.5)	39.7 (28.0, 53.5)	-0.52 (-0.61, -0.40)	-0.29 (-0.37, -0.19)	**-1.829 (-2.361, -1.296)**	**<0.001**	55089.1 (37019.5, 76415.9)	52.0 (35.5, 70.7)	-0.41 (-0.53, -0.23)	-0.27 (-0.38, -0.15)	**-1.677 (-2.311, -1.043)**	**<0.001**
**SDI**												
High SDI	1902638.4 (1611117.3, 2086568.1)	152.8 (134.2, 164.5)	-0.52 (-0.54, -0.50)	-0.03 (-0.05, -0.00)	**-6.224 (-6.661, -5.786)**	**<0.001**	2465734.3 (2294861.1, 2632967.3)	269.6 (251.9, 288.1)	-0.53 (-0.55, -0.51)	-0.03 (-0.06, -0.01)	**-11.161 (-12.025, -10.296)**	**<0.001**

High-middle SDI	3473146.1<br>(3020078.4, 3918439.3)	311.3<br>(271.8, 351.0)	-0.51<br>(-0.56, -0.45)	0.00<br>(-0.11, 0.14)	**-13.605**<br>**(-14.595, -12.615)**	**<0.001**	4472760.8<br>(3951418.4, 5082865.5)	531.8<br>(469.3, 601.5)	-0.46<br>(-0.53, -0.39)	-0.02<br>(-0.16, 0.14)	**-19.264**<br>**(-20.884, -17.645)**	**<0.001**
Middle SDI	3842799.7<br>(3302636.2, 4447082.2)	284.1<br>(244.3, 328.2)	-0.53<br>(-0.60, -0.44)	-0.01<br>(-0.15, 0.17)	**-11.736**<br>**(-12.622, -10.851)**	**<0.001**	5601812.4<br>(4823651.7, 6549071.1)	475.3<br>(409.6, 553.7)	-0.38<br>(-0.48, -0.26)	-0.01<br>(-0.19, 0.19)	**-10.664**<br>**(-11.500, -9.828)**	**<0.001**
Low-middle SDI	1934249.7<br>(1605404.1, 2322576.6)	261.8<br>(216.9, 314.8)	-0.21<br>(-0.30, -0.11)	-0.12<br>(-0.19, -0.05)	**-2.486**<br>**(-3.196, -1.776)**	**<0.001**	2630879.0<br>(2144274.8, 3254736.7)	376.1<br>(304.3, 464.0)	-0.08<br>(-0.18, 0.05)	-0.10<br>(-0.18, -0.02)	**-1.266**<br>**(-2.251, -0.281)**	**0.013**
Low SDI	652039.9<br>(558256.7, 753034.8)	251.7<br>(213.7, 291.7)	-0.27<br>(-0.34, -0.18)	-0.08<br>(-0.15, 0.01)	**-3.792**<br>**(-4.610, -2.975)**	**<0.001**	799830.2<br>(685164.1, 941610.1)	315.6<br>(268.3, 371.3)	-0.23<br>(-0.31, -0.13)	-0.07<br>(-0.16, 0.03)	**-3.835**<br>**(-4.769, -2.901)**	**<0.001**

ASRDALYs, age-standardized rates of DALYs; DALYs, disability-adjusted life-years; SDI, socio-demographic index.

Table S8. ASMR and ASRDALYs of cause-specific non-communicable diseases attributable to Hi-Tem and Lo-Tem in 2021, and the temporal trends from 1990 to 2021.

Cause	Hi-Tem-related deaths			Lo-Tem-related deaths			Hi-Tem-related DALYs			Lo-Tem-related DALYs		
	**ASMR**	**Trends of age-standardized rate from 1990 to 2021 (β)**	***P* value**	**ASMR**	**Trends of age-standardized rate from 1990 to 2021 (β)**	***P* value**	**ASRDALYs**	**Trends of age-standardized rate from 1990 to 2021 (β)**	***P* value**	**ASRDALYs**	**Trends of age-standardized rate from 1990 to 2021 (β)**	***P* value**
**Cardiovascular diseases**	2.45 (1.09, 4.54)	**0.0269 (0.0187, 0.0350)**	**<0.001**	12.54 (11.11, 14.05)	**-0.3543 (-0.3751, -0.3335)**	**<0.001**	55.79 (26.25, 99.59)	**0.5864 (0.3971, 0.7757)**	**<0.001**	232.49 (209.18, 261.17)	**-6.8877 (-7.2918, -6.4836)**	**<0.001**
Ischemic heart disease	1.34 (0.20, 3.07)	**0.0195 (0.0152, 0.0238)**	**<0.001**	6.14 (5.23, 7.53)	**-0.1437 (-0.1547, -0.1327)**	**<0.001**	30.57 (5.41, 66.96)	**0.4518 (0.3513, 0.5524)**	**<0.001**	117.40 (101.19, 144.86)	**-2.6886 (-2.9030, -2.4742)**	**<0.001**
Stroke	0.94 (0.12, 2.25)	0.0049 (0.0016, 0.0082)	0.005	5.01 (4.29, 5.85)	**-0.1870 (-0.1974, -0.1767)**	**<0.001**	21.01 (3.80, 48.31)	**0.0792 (0.0053, 0.1532)**	**0.037**	89.92 (76.78, 106.36)	**-3.6794 (-3.8744, -3.4845)**	**<0.001**
Ischemic stroke	0.44 (0.02, 1.05)	**0.0043 (0.0030, 0.0056)**	**<0.001**	2.73 (2.32, 3.17)	**-0.1054 (-0.1114, -0.0994)**	**<0.001**	7.87 (0.87, 18.36)	**0.0723 (0.0489, 0.0957)**	**<0.001**	41.52 (35.74, 48.31)	**-1.6371 (-1.7302, -1.5440)**	**<0.001**
Intracerebral hemorrhage	0.46 (0.08, 1.07)	0.0010 (-0.0008, 0.0028)	0.271	2.05 (1.74, 2.45)	**-0.0649 (-0.0703, -0.0594)**	**<0.001**	11.71 (2.66, 26.16)	0.0152 (-0.0299, 0.0602)	0.497	42.80 (35.87, 51.74)	**-1.6014 (-1.7099, -1.4929)**	**<0.001**
Subarachnoid hemorrhage	0.05 (0.01, 0.10)	**-0.0004 (-0.0006, -0.0002)**	**<0.001**	0.23 (0.19, 0.28)	**-0.0168 (-0.0185, -0.0151)**	**<0.001**	1.43 (0.27, 3.14)	**-0.0082 (-0.0143, -0.0021)**	**0.010**	5.60 (4.62, 6.77)	**-0.4410 (-0.4855, -0.3965)**	**<0.001**
Hypertensive heart disease	0.11 (-0.06, 0.33)	**0.0016 (0.0011, 0.0021)**	**<0.001**	1.11 (0.93, 1.28)	**-0.0142 (-0.0167, -0.0117)**	**<0.001**	2.24 (-0.99, 6.37)	**0.0304 (0.0210, 0.0397)**	**<0.001**	18.17 (15.09, 20.84)	**-0.3362 (-0.3844, -0.2880)**	**<0.001**
Cardiomyopathy and myocarditis	0.06 (-0.02, 0.15)	**0.0009 (0.0005, 0.0012)**	**<0.001**	0.27 (0.21, 0.34)	**-0.0094 (-0.0101, -0.0087)**	**<0.001**	1.97 (-0.80, 4.76)	**0.0249 (0.0146, 0.0352)**	**<0.001**	7.01 (5.35, 9.03)	**-0.1834 (-0.2125, -0.1543)**	**<0.001**
Myocarditis	0.01 (-0.00, 0.02)	**0.0001 (0.0000, 0.0001)**	**<0.001**	0.02 (0.02, 0.03)	**-0.0006 (-0.0008, -0.0005)**	**<0.001**	0.28 (-0.03, 0.61)	0.0006 (-0.0007, 0.0019)	0.327	0.65 (0.47, 0.88)	**-0.0227 (-0.0249, -0.0204)**	**<0.001**
Alcoholic cardiomyopathy	0.00 (-0.00, 0.01)	0.0000 (0.0000, 0.0001)	0.083	0.05 (0.04, 0.06)	**-0.0016 (-0.0025, -0.0008)**	**<0.001**	0.11 (-0.11, 0.36)	0.0016 (-0.0001, 0.0034)	0.063	1.56 (1.21, 1.92)	**-0.0448 (-0.0786, -0.0109)**	**0.011**
Other cardiomyopathy	0.05 (-0.02, 0.13)	**0.0007 (0.0005, 0.0010)**	**<0.001**	0.20 (0.16, 0.25)	**-0.0071 (-0.0078, -0.0064)**	**<0.001**	1.58 (-0.65, 3.85)	**0.0226 (0.0144, 0.0309)**	**<0.001**	4.79 (3.56, 6.38)	**-0.1160 (-0.1296, -0.1024)**	**<0.001**
Chronic obstructive pulmonary disease	0.45 (-0.09, 1.24)	**0.0044 (0.0020, 0.0068)**	**<0.001**	3.69 (2.97, 4.48)	**-0.1355 (-0.1453, -0.1257)**	**<0.001**	8.18 (-1.42, 22.29)	**0.0617 (0.0171, 0.1064)**	**0.008**	59.22 (47.58, 72.37)	**-2.6485 (-2.8426, -2.4544)**	**<0.001**
Diabetes and kidney diseases	0.69 (0.31, 1.17)	**0.0124 (0.0105, 0.0143)**	**<0.001**	1.79 (1.54, 2.06)	-0.0015 (-0.0034, 0.0004)	0.125	17.32 (8.10, 28.63)	**0.2941 (0.2469, 0.3414)**	**<0.001**	37.13 (31.95, 43.35)	**-0.1626 (-0.2063, -0.1189)**	**<0.001**
Diabetes mellitus	0.49 (0.16, 0.88)	**0.0084 (0.0071, 0.0096)**	**<0.001**	0.79 (0.61, 1.05)	**-0.0058 (-0.0069, -0.0047)**	**<0.001**	11.58 (3.91, 20.62)	**0.1868 (0.1562, 0.2175)**	**<0.001**	16.86 (12.47, 22.77)	**-0.1420 (-0.1672, -0.1169)**	**<0.001**
Diabetes mellitus type 1	0.02 (0.01, 0.03)	**0.0001 (0.0001, 0.0002)**	**<0.001**	0.02 (0.02, 0.03)	**-0.0008 (-0.0008, -0.0007)**	**<0.001**	0.85 (0.29, 1.52)	**0.0071 (0.0047, 0.0096)**	**<0.001**	1.06 (0.73, 1.51)	**-0.0298 (-0.0323, -0.0272)**	**<0.001**

Diabetes mellitus type 2	0.47<br>(0.15, 0.85)	**0.0082**<br>**(0.0070, 0.0095)**	**<0.001**	0.77<br>(0.59, 1.02)	**-0.0050**<br>**(-0.0061, -0.0040)**	**<0.001**	10.74<br>(3.64, 19.13)	**0.1797**<br>**(0.1512, 0.2083)**	**<0.001**	15.80<br>(11.74, 21.22)	**-0.1123**<br>**(-0.1351, -0.0895)**	**<0.001**
Chronic kidney disease	0.21<br>(-0.01, 0.49)	**0.0041**<br>**(0.0035, 0.0047)**	**<0.001**	0.99<br>(0.84, 1.12)	**0.0043**<br>**(0.0032, 0.0053)**	**<0.001**	5.74<br>(0.13, 12.99)	**0.1073**<br>**(0.0905, 0.1241)**	**<0.001**	20.27<br>(17.11, 23.53)	-0.0206<br>(-0.0421, 0.0010)	0.060

ASMR, age-standardized mortality rates; ASRDALYs, age-standardized rates of DALYs; DALYs, disability-adjusted life-years; Hi-Tem, high temperature; Lo-Tem, low temperature.

Table S9. Deaths and DALYs attributable to non-communicable diseases associated with high temperature in 204 countries and territories in 2021 and relative change from 1990 to 2021 and 2019 to 2021.

**Categories**	**Deaths**						**DALYs**					
**Location**	**Number of cases**	**ASMR**	**Relative change in ASMR 1990-2021**	**Relative change in ASMR 2019-2021**	**Trends of age-standardized rate from 1990 to 2021 (β)**	***P* value**	**Number of cases**	**ASRDALYs**	**Relative change in ASRDALYs 1990-2021**	**Relative change in ASRDALYs 2019-2021**	**Trends of age-standardized rate from 1990 to 2021 (β)**	***P* value**
Afghanistan	695.8 (314.8, 1300.8)	8.3 (3.8, 15.0)	0.14 (-0.12, 0.72)	0.07 (0.02, 0.16)	**0.058 (0.024, 0.092)**	**0.001**	20356.3 (9298.0, 37656.5)	179.5 (81.3, 335.1)	0.09 (-0.17, 0.67)	0.08 (0.02, 0.16)	**0.994 (0.235, 1.753)**	**0.012**
Albania	37.3 (9.7, 100.3)	0.9 (0.2, 2.5)	4.24 (-96.74, 146.32)	0.66 (0.21, 2.96)	**0.012 (0.004, 0.020)**	**0.005**	604.9 (155.2, 1616.9)	14.6 (3.8, 38.9)	3.67 (-85.13, 100.46)	0.62 (0.19, 2.90)	**0.176 (0.038, 0.315)**	**0.014**
Algeria	1955.2 (1008.5, 3301.2)	7.7 (4.0, 13.2)	0.60 (0.21, 1.79)	0.22 (0.15, 0.30)	0.034 (-0.005, 0.073)	0.085	39170.8 (20237.2, 66299.8)	124.7 (64.5, 209.8)	0.47 (0.09, 1.52)	0.24 (0.16, 0.32)	0.153 (-0.532, 0.837)	0.652
American Samoa	0.1 (0.0, 0.1)	0.2 (0.0, 0.3)	-1.65 (-5.50, 9.41)	-0.77 (-0.96, -0.68)	**0.021 (0.009, 0.033)**	**0.001**	1.8 (0.2, 3.4)	3.5 (0.3, 6.8)	-1.67 (-3.07, 7.96)	-0.77 (-0.93, -0.68)	**0.463 (0.201, 0.724)**	**0.001**
Andorra	-0.0 (-0.0, 0.0)	-0.0 (-0.0, 0.0)	NA	-2.97 (-3.08, 0.16)	**0.000 (0.000, 0.000)**	**<0.001**	-0.0 (-0.1, 0.1)	-0.0 (-0.0, 0.0)	NA	-1.08 (-2.67, 0.41)	**0.002 (0.001, 0.003)**	**<0.001**
Angola	11.1 (-113.2, 98.5)	0.0 (-1.4, 1.0)	-1.01 (-5.09, 2.01)	-0.77 (-2.79, 2.18)	**0.100 (0.092, 0.108)**	**<0.001**	511.6 (-2864.4, 2883.7)	2.2 (-26.0, 21.7)	-1.04 (-6.18, 2.21)	-0.58 (-1.97, 2.70)	**2.154 (1.990, 2.318)**	**<0.001**
Antigua and Barbuda	0.7 (0.5, 0.8)	0.7 (0.5, 0.9)	-0.08 (-0.19, 0.06)	-0.27 (-0.37, -0.17)	0.004 (-0.008, 0.016)	0.486	14.1 (10.4, 17.6)	13.4 (9.9, 16.9)	-0.14 (-0.26, -0.00)	-0.26 (-0.36, -0.15)	0.045 (-0.191, 0.281)	0.701
Argentina	259.1 (45.0, 539.9)	0.5 (0.1, 0.9)	-0.49 (-0.71, -0.28)	-0.38 (-0.64, -0.31)	0.000 (-0.005, 0.005)	0.985	4923.4 (990.6, 10125.4)	8.9 (1.8, 18.3)	-0.50 (-0.70, -0.33)	-0.37 (-0.62, -0.31)	-0.030 (-0.137, 0.077)	0.568
Armenia	62.7 (12.3, 168.5)	1.5 (0.3, 3.9)	0.38 (0.06, 3.38)	0.19 (0.09, 0.33)	0.002 (-0.011, 0.015)	0.721	1115.5 (223.9, 2986.3)	26.2 (5.3, 70.0)	0.34 (0.01, 3.41)	0.19 (0.08, 0.33)	0.037 (-0.192, 0.266)	0.740
Australia	74.9 (15.2, 180.2)	0.1 (0.0, 0.3)	-0.73 (-0.84, -0.53)	-0.54 (-0.68, -0.49)	**-0.007 (-0.010, -0.004)**	**<0.001**	1110.4 (243.0, 2600.2)	2.4 (0.6, 5.6)	-0.75 (-0.84, -0.55)	-0.54 (-0.69, -0.50)	**-0.133 (-0.180, -0.085)**	**<0.001**
Austria	35.3 (5.0, 113.3)	0.2 (0.0, 0.5)	-0.17 (-0.54, 1.90)	-0.38 (-0.51, -0.33)	-0.002 (-0.006, 0.002)	0.262	501.4 (75.8, 1608.0)	2.6 (0.4, 8.2)	-0.25 (-0.55, 1.40)	-0.39 (-0.51, -0.34)	-0.051 (-0.117, 0.016)	0.129
Azerbaijan	301.2 (102.4, 750.4)	3.7 (1.2, 9.6)	1.22 (0.61, 6.53)	0.40 (0.20, 0.80)	**0.051 (0.020, 0.082)**	**0.002**	6764.5 (2488.9, 16298.0)	70.6 (25.1, 172.0)	1.07 (0.50, 4.78)	0.42 (0.21, 0.80)	**0.754 (0.100, 1.408)**	**0.025**
Bahamas	5.3 (1.4, 9.1)	1.4 (0.4, 2.4)	1.53 (-22.02, 23.25)	-1.49 (-7.18, 4.13)	0.000 (-0.033, 0.033)	0.999	130.9 (37.3, 222.6)	31.7 (9.1, 54.0)	1.38 (-23.07, 24.41)	-1.53 (-9.43, 4.64)	0.005 (-0.693, 0.703)	0.988

Bahrain	176.4<br>(110.5, 249.2)	35.4<br>(22.5, 49.1)	-0.21<br>(-0.36, -0.02)	0.05<br>(-0.01, 0.11)	**-0.356<br>(-0.664, -0.047)**	**0.025**	4465.4<br>(2802.3, 6383.8)	592.0<br>(371.6, 829.5)	-0.29<br>(-0.43, -0.10)	0.05<br>(-0.02, 0.11)	**-9.246<br>(-14.757, -3.736)**	**0.002**
Bangladesh	9548.0<br>(4556.6, 15146.7)	8.0<br>(3.9, 12.7)	2.28<br>(-9.89, 16.22)	-0.12<br>(-0.17, -0.07)	**0.264<br>(0.156, 0.373)**	**<0.001**	208092.7<br>(99116.3, 332455.8)	154.6<br>(73.9, 246.2)	1.87<br>(-9.51, 13.78)	-0.12<br>(-0.18, -0.07)	**5.213<br>(2.875, 7.552)**	**<0.001**
Barbados	4.2<br>(2.9, 5.6)	0.8<br>(0.6, 1.1)	0.06<br>(-0.15, 0.33)	-0.21<br>(-0.29, -0.14)	0.006<br>(-0.007, 0.018)	0.361	76.0<br>(52.3, 103.1)	15.0<br>(10.3, 20.4)	0.00<br>(-0.21, 0.30)	-0.22<br>(-0.30, -0.15)	0.083<br>(-0.159, 0.324)	0.489
Belarus	42.3<br>(3.9, 192.4)	0.3<br>(0.0, 1.2)	-33.64<br>(-1258.58, 550.36)	1.40<br>(-4.37, 12.65)	**0.006<br>(0.003, 0.009)**	**<0.001**	750.3<br>(71.0, 3272.9)	4.7<br>(0.4, 20.1)	-24.21<br>(-1336.20, 992.55)	1.57<br>(-4.96, 9.39)	**0.109<br>(0.049, 0.169)**	**<0.001**
Belgium	7.4<br>(0.2, 30.2)	0.0<br>(0.0, 0.1)	-0.87<br>(-0.95, -0.76)	-0.81<br>(-0.97, -0.71)	**-0.003<br>(-0.006, -0.001)**	**0.019**	111.1<br>(3.8, 464.2)	0.5<br>(0.0, 1.9)	-0.87<br>(-0.95, -0.76)	-0.81<br>(-0.97, -0.70)	**-0.062<br>(-0.110, -0.015)**	**0.012**
Belize	5.3<br>(3.1, 8.3)	1.9<br>(1.1, 3.0)	5.28<br>(-93.23, 86.95)	-0.36<br>(-0.44, -0.14)	**0.044<br>(0.017, 0.071)**	**0.002**	133.1<br>(76.4, 210.9)	42.1<br>(24.5, 66.4)	5.00<br>(-110.09, 160.61)	-0.35<br>(-0.43, -0.14)	**0.987<br>(0.406, 1.567)**	**0.002**
Benin	375.6<br>(60.6, 516.5)	8.7<br>(1.5, 11.9)	2.63<br>(-12.50, 2.91)	0.17<br>(0.07, 2.28)	**0.183<br>(0.143, 0.224)**	**<0.001**	9322.2<br>(1317.6, 13295.8)	171.7<br>(27.4, 237.9)	2.51<br>(-10.87, 3.07)	0.17<br>(0.05, 2.55)	**3.569<br>(2.754, 4.384)**	**<0.001**
Bermuda	0.6<br>(0.0, 1.5)	0.4<br>(0.0, 1.0)	-0.07<br>(-0.66, 2.15)	-0.21<br>(-0.50, -0.12)	-0.001<br>(-0.005, 0.003)	0.664	10.3<br>(0.5, 25.7)	7.8<br>(0.4, 19.2)	-0.08<br>(-0.62, 1.71)	-0.20<br>(-0.50, -0.13)	-0.020<br>(-0.104, 0.064)	0.628
Bhutan	0.6<br>(-0.1, 1.8)	0.1<br>(-0.0, 0.3)	0.91<br>(-3.45, 6.21)	-0.12<br>(-1.23, 0.30)	**0.002<br>(0.001, 0.002)**	**<0.001**	12.7<br>(-1.4, 38.8)	2.1<br>(-0.2, 6.4)	0.72<br>(-3.73, 4.86)	-0.13<br>(-0.93, 0.20)	**0.029<br>(0.018, 0.041)**	**<0.001**
Bolivia (Plurinational State of)	51.3<br>(6.6, 100.8)	0.6<br>(0.1, 1.3)	-28.71<br>(-42.62, 51.08)	-0.00<br>(-0.10, 0.14)	**0.025<br>(0.020, 0.030)**	**<0.001**	1203.8<br>(180.8, 2319.3)	13.1<br>(1.8, 25.5)	24.87<br>(-26.86, 39.59)	0.00<br>(-0.11, 0.13)	**0.482<br>(0.373, 0.592)**	**<0.001**
Bosnia and Herzegovina	57.4<br>(10.0, 150.9)	0.9<br>(0.2, 2.4)	1.50<br>(0.47, 23.96)	0.33<br>(0.05, 2.63)	0.006<br>(-0.003, 0.016)	0.203	975.0<br>(176.0, 2573.8)	15.7<br>(2.9, 41.3)	1.27<br>(0.35, 23.28)	0.32<br>(0.02, 2.66)	0.074<br>(-0.102, 0.249)	0.398
Botswana	21.6<br>(0.6, 50.6)	1.9<br>(0.0, 4.3)	-0.45<br>(-0.99, 0.11)	-0.68<br>(-0.96, -0.63)	0.005<br>(-0.066, 0.075)	0.893	523.0<br>(19.4, 1207.7)	36.7<br>(1.4, 85.5)	-0.47<br>(-0.95, 0.15)	-0.68<br>(-0.91, -0.63)	-0.047<br>(-1.446, 1.352)	0.946
Brazil	875.6<br>(29.3, 1876.6)	0.4<br>(0.0, 0.8)	0.22<br>(-10.80, 6.21)	-0.52<br>(-0.71, -0.23)	**0.010<br>(0.002, 0.018)**	**0.013**	18507.0<br>(-463.5, 40014.0)	7.4<br>(-0.2, 16.0)	0.22<br>(-7.48, 6.01)	-0.52<br>(-0.73, -0.15)	**0.207<br>(0.044, 0.371)**	**0.015**
Brunei Darussalam	3.4<br>(2.5, 4.3)	1.3<br>(0.9, 1.7)	0.13<br>(-0.14, 0.68)	-0.41<br>(-0.52, -0.33)	**0.026<br>(0.007, 0.044)**	**0.008**	85.0<br>(60.2, 107.7)	24.8<br>(17.9, 31.5)	0.07<br>(-0.19, 0.55)	-0.39<br>(-0.51, -0.30)	**0.404<br>(0.058, 0.750)**	**0.024**
Bulgaria	250.2<br>(41.3, 700.8)	1.8<br>(0.3, 4.9)	0.34<br>(-0.24, 12.88)	0.38<br>(0.12, 2.31)	-0.010<br>(-0.032, 0.012)	0.343	4330.8<br>(727.8, 12009.2)	31.9<br>(5.4, 88.6)	0.46<br>(-0.13, 13.11)	0.38<br>(0.11, 2.25)	-0.133<br>(-0.523, 0.257)	0.491
Burkina Faso	1619.0<br>(1157.6, 2177.9)	20.6<br>(15.2, 27.3)	0.58<br>(0.18, 8.24)	0.08<br>(0.03, 0.16)	**0.292<br>(0.215, 0.370)**	**<0.001**	42247.2<br>(29201.7, 56984.3)	419.2<br>(297.1, 564.2)	0.51<br>(0.12, 7.54)	0.08<br>(0.01, 0.15)	**5.601<br>(4.029, 7.172)**	**<0.001**

Burundi	-1.8<br>(-9.2, 1.4)	-0.1<br>(-0.2, 0.0)	-0.38<br>(-2.53, 1.40)	-0.38<br>(-1.60, 0.40)	0.000<br>(-0.001, 0.001)	0.969	-46.3<br>(-248.6, 40.3)	-1.0<br>(-4.8, 0.7)	-0.44<br>(-2.79, 1.27)	-0.39<br>(-1.93, 0.79)	0.004<br>(-0.022, 0.030)	0.764
Cabo Verde	0.3<br>(-0.4, 1.3)	0.1<br>(-0.1, 0.3)	-0.26<br>(-3.97, 7.79)	0.45<br>(-1.81, 6.03)	0.001<br>(0.000, 0.003)	0.130	5.9<br>(-8.5, 24.5)	1.3<br>(-1.9, 5.6)	-0.35<br>(-3.22, 6.70)	0.46<br>(-1.07, 4.32)	0.021<br>(-0.013, 0.054)	0.220
Cambodia	951.0<br>(595.9, 1174.4)	9.5<br>(6.0, 11.6)	0.28<br>(-1.52, 1.77)	-0.43<br>(-0.52, -0.26)	**0.273**<br>**(0.126, 0.420)**	**<0.001**	22674.2<br>(13869.2, 28742.0)	188.9<br>(117.3, 235.0)	0.15<br>(-1.47, 1.42)	-0.43<br>(-0.53, -0.26)	**4.944**<br>**(1.868, 8.021)**	**0.003**
Cameroon	712.6<br>(467.2, 1032.7)	6.8<br>(4.6, 9.9)	0.46<br>(0.15, 1.04)	-0.01<br>(-0.09, 0.10)	**0.107**<br>**(0.071, 0.143)**	**<0.001**	19496.5<br>(12393.2, 28876.1)	141.8<br>(92.8, 205.6)	0.44<br>(0.11, 1.06)	-0.02<br>(-0.10, 0.10)	**2.180**<br>**(1.394, 2.967)**	**<0.001**
Canada	59.2<br>(4.6, 187.0)	0.1<br>(0.0, 0.2)	-0.28<br>(-4.99, 10.41)	0.83<br>(0.55, 2.35)	**-0.002**<br>**(-0.004, -0.001)**	**0.009**	962.6<br>(110.4, 2997.7)	1.3<br>(0.2, 4.1)	-0.33<br>(-4.55, 4.79)	0.82<br>(0.54, 1.81)	**-0.041**<br>**(-0.069, -0.013)**	**0.005**
Central African Republic	41.0<br>(-99.0, 120.2)	2.2<br>(-6.0, 6.6)	-4.64<br>(-2.65, 4.68)	0.02<br>(-3.08, 3.44)	**0.073**<br>**(0.009, 0.136)**	**0.026**	1271.7<br>(-2938.4, 3702.7)	50.3<br>(-126.0, 149.8)	-6.61<br>(-3.28, 3.60)	0.02<br>(-2.96, 4.94)	**1.630**<br>**(0.226, 3.034)**	**0.024**
Chad	1048.4<br>(671.7, 1515.0)	21.1<br>(13.7, 30.0)	0.36<br>(0.07, 0.83)	0.05<br>(-0.02, 0.15)	**0.305**<br>**(0.216, 0.394)**	**<0.001**	29407.9<br>(18633.6, 42759.0)	443.6<br>(283.6, 641.7)	0.35<br>(0.04, 0.83)	0.05<br>(-0.02, 0.15)	**6.314**<br>**(4.398, 8.231)**	**<0.001**
Chile	0.5<br>(-0.2, 3.6)	0.0<br>(-0.0, 0.0)	-0.26<br>(-7.31, 10.43)	-0.75<br>(-1.42, -0.43)	0.000<br>(0.000, 0.000)	0.733	11.8<br>(-1.8, 74.2)	0.0<br>(-0.0, 0.3)	0.09<br>(-6.74, 11.14)	-0.75<br>(-1.31, -0.51)	0.001<br>(-0.001, 0.003)	0.253
China	42506.2<br>(13117.0, 90228.0)	2.4<br>(0.7, 5.0)	-0.18<br>(-0.50, 1.04)	-0.01<br>(-0.20, 0.17)	-0.009<br>(-0.026, 0.008)	0.306	752871.4<br>(232272.4, 1619285.7)	39.1<br>(12.3, 83.4)	-0.23<br>(-0.52, 0.71)	-0.01<br>(-0.21, 0.20)	-0.261<br>(-0.532, 0.010)	0.059
Colombia	230.0<br>(149.2, 280.0)	0.4<br>(0.3, 0.5)	-1.40<br>(-1.73, -1.14)	-0.48<br>(-0.58, -0.35)	**0.069**<br>**(0.061, 0.078)**	**<0.001**	4347.0<br>(2880.6, 5330.3)	7.8<br>(5.2, 9.6)	-1.39<br>(-1.71, -1.13)	-0.48<br>(-0.58, -0.34)	**1.345**<br>**(1.183, 1.508)**	**<0.001**
Comoros	-1.0<br>(-2.9, 0.3)	-0.3<br>(-0.7, 0.1)	-0.56<br>(-5.01, 0.21)	-0.30<br>(-1.63, 0.38)	**0.008**<br>**(0.006, 0.011)**	**<0.001**	-23.5<br>(-66.8, 8.5)	-4.9<br>(-13.9, 1.6)	-0.59<br>(-6.86, 4.08)	-0.30<br>(-1.53, 0.16)	**0.193**<br>**(0.137, 0.248)**	**<0.001**
Congo	-4.7<br>(-24.3, 20.4)	-0.3<br>(-1.3, 0.9)	-0.67<br>(-2.36, 1.11)	0.16<br>(-2.23, 1.28)	**0.024**<br>**(0.018, 0.030)**	**<0.001**	-85.8<br>(-619.7, 583.4)	-4.8<br>(-24.1, 19.9)	-0.75<br>(-2.43, 1.00)	0.30<br>(-2.68, 1.63)	**0.566**<br>**(0.441, 0.690)**	**<0.001**
Cook Islands	0.1<br>(0.0, 0.2)	0.5<br>(-0.0, 1.0)	-0.65<br>(-1.02, -0.47)	-0.83<br>(-1.00, -0.73)	0.002<br>(-0.037, 0.041)	0.919	2.8<br>(0.2, 5.4)	10.7<br>(0.6, 21.0)	-0.65<br>(-0.97, -0.46)	-0.83<br>(-0.99, -0.73)	0.001<br>(-0.849, 0.851)	0.998
Costa Rica	1.4<br>(0.1, 1.9)	0.0<br>(0.0, 0.0)	-0.28<br>(-11.55, 10.33)	-0.63<br>(-0.95, 0.21)	0.001<br>(0.000, 0.002)	0.094	27.6<br>(2.9, 37.5)	0.5<br>(0.1, 0.7)	-0.32<br>(-2.23, 5.81)	-0.62<br>(-0.95, 0.31)	0.015<br>(-0.004, 0.034)	0.116
Croatia	113.6<br>(22.0, 306.6)	1.2<br>(0.2, 3.1)	0.48<br>(-23.66, 33.99)	0.24<br>(0.05, 1.61)	-0.006<br>(-0.021, 0.010)	0.465	1685.2<br>(341.7, 4478.3)	18.2<br>(3.8, 48.2)	0.39<br>(-21.02, 31.98)	0.23<br>(0.04, 1.51)	-0.121<br>(-0.380, 0.137)	0.345
Cte d'Ivoire	181.3<br>(-92.2, 290.7)	2.0<br>(-1.0, 3.2)	-4.15<br>(-4.44, 1.06)	0.07<br>(-1.63, 1.29)	**0.115**<br>**(0.089, 0.141)**	**<0.001**	4889.1<br>(-2550.8, 7968.8)	41.4<br>(-21.0, 66.4)	-4.27<br>(-4.70, 0.97)	0.06<br>(-1.64, 1.22)	**2.378**<br>**(1.826, 2.930)**	**<0.001**

Cuba	176.5 (107.2, 248.9)	0.9 (0.5, 1.2)	-0.14 (-0.35, 0.07)	-0.26 (-0.35, -0.16)	-0.002 (-0.019, 0.015)	0.818	3225.3 (1941.2, 4550.8)	16.6 (9.9, 23.5)	-0.15 (-0.36, 0.07)	-0.27 (-0.36, -0.17)	-0.051 (-0.385, 0.282)	0.756
Cyprus	59.4 (27.0, 110.9)	3.5 (1.6, 6.6)	-0.35 (-0.53, 0.18)	0.03 (-0.08, 0.28)	**-0.112 (-0.162, -0.062)**	**<0.001**	929.9 (418.0, 1733.5)	50.6 (22.7, 94.1)	-0.36 (-0.53, 0.16)	0.02 (-0.09, 0.29)	**-1.635 (-2.374, -0.896)**	**<0.001**
Czechia	26.2 (2.0, 110.1)	0.1 (0.0, 0.5)	-0.29 (-0.77, 3.53)	-0.53 (-0.76, -0.43)	0.000 (-0.004, 0.004)	0.936	396.2 (33.4, 1632.4)	1.8 (0.2, 7.4)	-0.42 (-0.66, 3.45)	-0.53 (-0.75, -0.43)	-0.022 (-0.089, 0.045)	0.506
Democratic People's Republic of Korea	620.2 (82.7, 1570.6)	2.1 (0.3, 5.4)	0.54 (-2.46, 7.20)	-0.04 (-0.10, 0.07)	0.017 (-0.001, 0.035)	0.062	13138.8 (1554.5, 34932.6)	41.5 (5.2, 108.9)	0.60 (-2.57, 5.08)	-0.04 (-0.10, 0.07)	**0.361 (0.025, 0.697)**	**0.036**
Democratic Republic of the Congo	30.1 (-463.5, 315.6)	0.0 (-1.7, 1.1)	-1.01 (-3.89, 0.03)	-1.06 (-10.23, 13.23)	**0.031 (0.023, 0.038)**	**<0.001**	1454.9 (-11789.2, 9132.3)	2.0 (-33.5, 22.5)	-1.06 (-4.43, -0.11)	-1.37 (-6.36, 10.46)	**0.686 (0.537, 0.835)**	**<0.001**
Denmark	1.0 (0.0, 4.3)	0.0 (0.0, 0.0)	-0.65 (-3.57, 2.36)	0.02 (-3.15, 4.15)	**-0.001 (-0.002, 0.000)**	**0.006**	15.0 (0.1, 66.0)	0.1 (0.0, 0.5)	-0.66 (-2.69, 2.09)	-0.01 (-2.07, 3.19)	**-0.019 (-0.032, -0.006)**	**0.006**
Djibouti	133.7 (89.8, 194.3)	28.1 (19.6, 40.5)	-0.17 (-0.38, 0.13)	0.04 (-0.03, 0.15)	0.052 (-0.070, 0.174)	0.390	3668.2 (2434.1, 5326.3)	566.8 (383.7, 824.8)	-0.20 (-0.42, 0.13)	0.04 (-0.04, 0.15)	0.427 (-2.089, 2.943)	0.731
Dominica	0.4 (0.2, 0.5)	0.5 (0.3, 0.7)	1.03 (0.47, 3.03)	-0.17 (-0.25, -0.11)	0.009 (0.000, 0.017)	0.057	7.6 (4.5, 11.0)	9.2 (5.3, 13.3)	0.93 (0.41, 2.45)	-0.17 (-0.25, -0.11)	0.150 (-0.021, 0.321)	0.084
Dominican Republic	30.7 (16.0, 46.9)	0.3 (0.2, 0.5)	-3.14 (-4.73, 0.74)	-0.44 (-0.57, -0.21)	**0.025 (0.020, 0.030)**	**<0.001**	741.3 (394.6, 1123.4)	7.2 (3.8, 11.0)	-4.25 (-7.98, 0.78)	-0.45 (-0.57, -0.21)	**0.550 (0.441, 0.658)**	**<0.001**
Ecuador	-7.7 (-27.9, 8.8)	-0.1 (-0.2, 0.1)	-0.76 (-1.31, 0.00)	0.18 (-4.49, 1.67)	**0.005 (0.002, 0.009)**	**0.002**	-113.8 (-501.7, 207.4)	-0.8 (-3.2, 1.2)	-0.81 (-1.40, -0.06)	0.93 (-2.98, 0.47)	**0.118 (0.059, 0.176)**	**<0.001**
Egypt	10694.6 (3550.7, 20160.9)	23.6 (7.9, 44.3)	1.60 (-0.59, 13.50)	0.01 (-0.12, 0.18)	**0.421 (0.315, 0.528)**	**<0.001**	270519.3 (91506.9, 510958.7)	453.3 (151.8, 854.3)	1.49 (0.51, 11.80)	0.01 (-0.13, 0.20)	**7.396 (5.333, 9.459)**	**<0.001**
El Salvador	41.3 (3.4, 88.6)	0.7 (0.1, 1.4)	-1.35 (-3.81, -1.02)	-0.27 (-0.98, 0.44)	**0.082 (0.063, 0.101)**	**<0.001**	1026.3 (249.5, 2003.6)	16.9 (4.5, 32.4)	-1.42 (-4.73, -1.05)	-0.26 (-0.72, 0.37)	**1.811 (1.404, 2.217)**	**<0.001**
Equatorial Guinea	-2.1 (-6.9, 0.0)	-0.6 (-1.7, -0.0)	-0.37 (-0.95, 1.27)	0.01 (-0.76, 0.67)	**0.020 (0.014, 0.027)**	**<0.001**	-51.5 (-180.5, 3.2)	-10.3 (-34.0, 0.1)	-0.45 (-0.99, 1.05)	0.02 (-1.00, 0.66)	**0.479 (0.348, 0.610)**	**<0.001**
Eritrea	251.2 (161.9, 354.4)	11.8 (7.7, 16.3)	0.13 (-0.10, 0.73)	0.14 (0.10, 0.24)	**0.059 (0.020, 0.098)**	**0.004**	7231.1 (4545.5, 10352.7)	245.5 (158.6, 343.3)	0.04 (-0.20, 0.56)	0.15 (0.09, 0.24)	0.645 (-0.205, 1.496)	0.132
Estonia	3.8 (-0.7, 9.6)	0.1 (-0.0, 0.3)	-7.78 (-59333.57, 522.28)	4.91 (-20.59, 22.19)	**0.003 (0.002, 0.005)**	**<0.001**	55.8 (-6.1, 140.7)	2.0 (-0.2, 5.1)	-6.54 (-66553.96, 101162.72)	4.91 (-16.80, 33.03)	**0.057 (0.029, 0.086)**	**<0.001**

Eswatini	2.8 (-1.1, 8.1)	0.6 (-0.2, 1.8)	1.48 (-6.20, 10.92)	-0.38 (-1.08, 0.44)	0.015 (-0.001, 0.032)	0.072	76.5 (-27.4, 216.4)	13.1 (-5.0, 37.3)	1.53 (-6.54, 10.26)	-0.36 (-1.09, 0.57)	0.334 (-0.023, 0.691)	0.066
Ethiopia	230.2 (116.8, 361.1)	0.5 (0.3, 0.9)	-0.22 (-0.76, 1.12)	-0.21 (-0.38, 0.08)	**-0.005 (-0.010, -0.001)**	**0.031**	6857.6 (3578.0, 10661.6)	13.0 (6.7, 20.4)	-0.38 (-0.61, 0.40)	-0.20 (-0.38, 0.08)	**-0.314 (-0.440, -0.189)**	**<0.001**
Fiji	4.5 (1.3, 8.5)	0.7 (0.2, 1.3)	18.45 (-42.62, 22.24)	0.22 (-0.01, 0.51)	**0.017 (0.011, 0.023)**	**<0.001**	118.7 (34.8, 226.5)	14.9 (4.1, 28.5)	13.79 (-38.79, 53.05)	0.22 (-0.01, 0.49)	**0.371 (0.235, 0.507)**	**<0.001**
Finland	5.0 (-0.3, 20.6)	0.0 (-0.0, 0.1)	-20.02 (-1006.25, 1263.75)	2.21 (-7.10, 8.88)	0.000 (0.000, 0.001)	0.069	72.8 (-2.5, 285.3)	0.6 (-0.0, 2.2)	-89.22 (-848.89, 719.48)	2.18 (-4.55, 7.66)	0.007 (-0.003, 0.016)	0.156
France	82.1 (1.9, 314.1)	0.0 (0.0, 0.2)	-0.78 (-0.93, -0.69)	-0.73 (-0.97, -0.61)	-0.002 (-0.005, 0.001)	0.225	1129.1 (38.1, 4307.6)	0.7 (0.0, 2.8)	-0.79 (-0.92, -0.70)	-0.72 (-0.95, -0.60)	-0.031 (-0.080, 0.017)	0.194
Gabon	-1.2 (-7.2, 2.9)	-0.2 (-0.9, 0.3)	-0.39 (-12.90, 2.29)	0.38 (-4.24, 3.35)	**0.004 (0.001, 0.008)**	**0.015**	-21.4 (-162.9, 76.6)	-2.7 (-17.0, 7.0)	-0.52 (-18.67, 5.99)	0.92 (-3.35, 1.52)	**0.122 (0.048, 0.196)**	**0.002**
Gambia	76.1 (48.3, 104.9)	9.1 (5.8, 12.4)	1.08 (0.26, 4.89)	0.15 (0.08, 0.32)	**0.178 (0.130, 0.226)**	**<0.001**	1892.2 (1189.2, 2651.3)	184.0 (116.1, 255.0)	1.01 (0.21, 4.64)	0.14 (0.07, 0.30)	**3.437 (2.439, 4.436)**	**<0.001**
Georgia	77.7 (13.6, 208.2)	1.2 (0.2, 3.3)	0.28 (-0.40, 7.52)	0.40 (0.20, 1.27)	-0.002 (-0.020, 0.016)	0.814	1411.8 (265.4, 3686.7)	24.0 (4.7, 62.8)	0.32 (-0.30, 7.35)	0.38 (0.19, 1.21)	-0.073 (-0.449, 0.303)	0.695
Germany	151.6 (23.0, 554.9)	0.1 (0.0, 0.2)	-0.67 (-0.78, -0.02)	-0.70 (-0.82, -0.60)	**-0.004 (-0.007, 0.000)**	**0.043**	2250.7 (358.7, 8384.7)	1.1 (0.2, 4.2)	-0.68 (-0.79, -0.14)	-0.69 (-0.82, -0.59)	**-0.066 (-0.124, -0.007)**	**0.030**
Ghana	1158.1 (838.7, 1514.5)	8.5 (6.2, 10.9)	1.02 (0.42, 3.56)	0.01 (-0.07, 0.13)	**0.144 (0.100, 0.187)**	**<0.001**	29938.1 (21367.4, 40064.7)	173.3 (125.2, 226.9)	0.93 (0.33, 3.39)	-0.01 (-0.08, 0.12)	**2.890 (1.949, 3.831)**	**<0.001**
Greece	271.9 (110.5, 547.4)	0.9 (0.4, 1.8)	1.08 (0.19, 6.87)	0.91 (0.50, 2.67)	0.002 (-0.008, 0.012)	0.657	3950.4 (1591.1, 7964.9)	16.2 (6.2, 32.9)	1.18 (0.28, 7.68)	0.85 (0.46, 2.69)	0.065 (-0.097, 0.227)	0.420
Greenland	-0.0 (-0.3, 0.1)	-0.1 (-0.5, 0.1)	-0.95 (-2.68, -0.72)	-0.17 (-26.39, 52.71)	**0.039 (0.029, 0.049)**	**<0.001**	-0.7 (-5.7, 1.7)	-1.4 (-9.1, 2.4)	-0.96 (-3.12, -0.70)	-0.28 (-24.63, 50.90)	**0.746 (0.555, 0.937)**	**<0.001**
Grenada	1.2 (0.9, 1.5)	1.2 (0.9, 1.5)	0.39 (0.13, 0.71)	-0.20 (-0.28, -0.14)	**0.021 (0.003, 0.038)**	**0.021**	26.0 (19.2, 32.8)	23.2 (17.3, 29.4)	0.29 (0.04, 0.62)	-0.19 (-0.28, -0.13)	**0.366 (0.019, 0.714)**	**0.040**
Guam	1.8 (1.0, 3.0)	0.9 (0.4, 1.4)	1.80 (-45.71, 17.41)	0.00 (-0.14, 1.17)	**0.033 (0.012, 0.055)**	**0.004**	45.7 (24.5, 76.2)	21.8 (11.5, 37.0)	2.99 (-33.80, 30.87)	0.07 (-0.09, 1.46)	**0.857 (0.371, 1.343)**	**0.001**
Guatemala	41.4 (21.9, 67.0)	0.4 (0.2, 0.6)	-1.73 (-10.95, 10.73)	-0.43 (-0.57, -0.28)	**0.041 (0.032, 0.049)**	**<0.001**	1084.1 (623.0, 1699.5)	9.3 (5.3, 14.8)	-1.92 (-15.85, 11.45)	-0.40 (-0.52, -0.26)	**0.835 (0.679, 0.992)**	**<0.001**
Guinea	194.4 (52.0, 298.3)	3.9 (1.0, 5.9)	1.80 (-22.71, 30.51)	0.23 (0.12, 2.02)	**0.075 (0.054, 0.095)**	**<0.001**	5012.1 (1401.4, 7730.1)	81.4 (22.0, 125.1)	1.65 (-22.18, 30.89)	0.23 (0.11, 1.69)	**1.517 (1.079, 1.954)**	**<0.001**

Guinea-Bissau	53.3 (23.2, 87.1)	9.2 (4.0, 14.9)	0.76 (-2.93, 12.00)	0.03 (-0.10, 0.50)	**0.122 (0.032, 0.212)**	**0.010**	1538.6 (626.8, 2524.9)	195.7 (86.1, 319.5)	0.62 (-2.65, 10.34)	0.02 (-0.10, 0.49)	**2.305 (0.304, 4.306)**	**0.025**
Guyana	2.8 (-5.3, 7.0)	0.5 (-1.0, 1.2)	0.06 (-3.30, 1.28)	-0.23 (-2.45, 2.24)	0.013 (-0.002, 0.029)	0.095	68.8 (-134.9, 179.4)	10.4 (-20.8, 27.0)	-0.07 (-3.19, 3.40)	-0.23 (-2.85, 1.73)	0.245 (-0.098, 0.587)	0.155
Haiti	49.2 (-9.3, 106.7)	0.8 (-0.2, 1.7)	2.45 (-2.61, 1.35)	-0.44 (-1.39, 0.07)	-0.099 (-0.748, 0.549)	0.757	1348.0 (-236.0, 2887.6)	17.4 (-3.6, 37.9)	1.19 (-2.92, 1.10)	-0.43 (-1.24, 0.04)	-2.162 (-16.133, 11.809)	0.754
Honduras	68.9 (35.3, 112.2)	1.3 (0.6, 2.1)	-5.58 (-452.87, 232.60)	-0.28 (-0.34, -0.14)	0.179 (-0.142, 0.501)	0.263	1556.9 (835.5, 2534.2)	25.0 (13.1, 40.8)	-6.11 (-74.27, 299.48)	-0.27 (-0.33, -0.16)	3.434 (-2.736, 9.604)	0.265
Hungary	341.3 (72.5, 905.0)	1.6 (0.4, 4.2)	0.50 (-0.11, 6.15)	0.07 (-0.06, 0.73)	-0.004 (-0.024, 0.016)	0.700	5733.9 (1341.5, 14821.2)	29.4 (7.1, 75.2)	0.40 (-0.10, 5.27)	0.10 (-0.05, 0.74)	-0.170 (-0.546, 0.205)	0.361
Iceland	0.0 (-0.0, 0.0)	0.0 (-0.0, 0.0)	NA	NA	0.000 (0.000, 0.000)	0.541	0.0 (-0.0, 0.0)	0.0 (-0.0, 0.0)	NA	NA	0.000 (0.000, 0.000)	0.385
India	104887.8 (63793.1, 150538.6)	9.9 (6.0, 14.3)	0.27 (0.07, 0.70)	-0.35 (-0.41, -0.29)	**0.137 (0.072, 0.203)**	**<0.001**	2477660.1 (1530638.3, 3521542.2)	208.1 (127.5, 296.7)	0.20 (0.01, 0.59)	-0.35 (-0.42, -0.28)	**2.293 (0.960, 3.625)**	**0.001**
Indonesia	2518.2 (1940.1, 3174.7)	1.3 (1.0, 1.6)	3.62 (-19.87, 22.49)	-0.40 (-0.53, -0.21)	**0.056 (0.043, 0.068)**	**<0.001**	63910.7 (48954.4, 80460.2)	26.8 (20.6, 33.7)	3.59 (-20.93, 31.47)	-0.40 (-0.54, -0.21)	**1.162 (0.890, 1.435)**	**<0.001**
Iran (Islamic Republic of)	4940.8 (2483.8, 8337.8)	7.3 (3.7, 12.4)	0.24 (-0.05, 2.03)	0.02 (-0.04, 0.07)	-0.011 (-0.038, 0.015)	0.393	103402.9 (51628.2, 174239.3)	137.0 (68.5, 230.2)	0.20 (-0.09, 1.88)	0.01 (-0.06, 0.07)	-0.216 (-0.705, 0.272)	0.373
Iraq	10584.7 (5461.8, 17031.7)	56.9 (29.3, 89.8)	0.28 (0.01, 0.57)	0.17 (0.07, 0.25)	0.032 (-0.152, 0.216)	0.722	251882.0 (132483.5, 409988.1)	1088.1 (562.6, 1757.1)	0.10 (-0.15, 0.40)	0.13 (0.06, 0.21)	-3.789 (-7.620, 0.041)	0.052
Ireland	0.8 (-0.0, 4.1)	0.0 (-0.0, 0.0)	NA	NA	0.000 (-0.001, 0.000)	0.157	13.0 (-0.1, 66.4)	0.2 (-0.0, 0.8)	NA	NA	-0.006 (-0.015, 0.002)	0.131
Israel	176.4 (37.8, 381.6)	1.3 (0.3, 2.7)	0.51 (-2.93, 20.57)	0.04 (-0.05, 0.65)	-0.006 (-0.022, 0.009)	0.408	2626.6 (593.3, 5617.9)	20.4 (4.7, 43.7)	0.38 (-2.47, 21.28)	0.03 (-0.06, 0.63)	-0.173 (-0.438, 0.092)	0.193
Italy	613.2 (207.2, 1465.1)	0.3 (0.1, 0.8)	0.65 (-0.10, 8.14)	0.04 (-0.06, 0.37)	-0.002 (-0.008, 0.004)	0.429	8067.7 (2747.0, 19449.7)	4.9 (1.7, 12.0)	0.53 (-0.12, 8.36)	0.04 (-0.06, 0.41)	-0.052 (-0.147, 0.042)	0.267
Jamaica	37.7 (20.2, 60.2)	1.2 (0.6, 1.9)	0.99 (0.47, 1.56)	-0.31 (-0.41, -0.17)	**0.031 (0.018, 0.043)**	**<0.001**	723.8 (371.5, 1151.8)	23.2 (11.9, 36.8)	0.84 (0.31, 1.42)	-0.31 (-0.42, -0.17)	**0.588 (0.335, 0.840)**	**<0.001**
Japan	840.5 (157.3, 2073.2)	0.2 (0.0, 0.4)	-0.73 (-0.83, -0.68)	-0.29 (-0.54, -0.21)	**-0.005 (-0.010, -0.001)**	**0.029**	11681.4 (2191.4, 29059.4)	3.3 (0.7, 8.3)	-0.71 (-0.83, -0.65)	-0.28 (-0.52, -0.21)	**-0.092 (-0.179, -0.006)**	**0.037**
Jordan	141.8 (40.6, 310.6)	2.5 (0.8, 5.6)	0.98 (-10.80, 17.46)	0.13 (0.03, 0.36)	0.019 (-0.013, 0.050)	0.234	3365.3 (921.6, 7340.4)	46.9 (13.4, 102.8)	0.78 (-9.34, 8.96)	0.12 (0.02, 0.36)	0.185 (-0.440, 0.810)	0.55

Kazakhstan	459.6<br>(163.3, 945.7)	3.2<br>(1.1, 6.8)	1.47<br>(-10.36, 11.76)	0.24<br>(0.15, 0.36)	**0.024<br>(0.002, 0.046)**	**0.032**	10203.2<br>(3806.7, 20020.2)	60.2<br>(22.2, 120.6)	1.40<br>(-7.84, 16.91)	0.25<br>(0.14, 0.37)	0.403<br>(-0.086, 0.892)	0.103
Kenya	57.4<br>(14.1, 93.8)	0.3<br>(0.1, 0.5)	-4.94<br>(-94.14, 24.40)	-0.30<br>(-0.64, 0.12)	**0.012<br>(0.009, 0.015)**	**<0.001**	1466.1<br>(330.2, 2397.6)	6.2<br>(1.5, 10.1)	-4.87<br>(-50.74, 25.81)	-0.31<br>(-0.64, 0.05)	**0.254<br>(0.192, 0.316)**	**<0.001**
Kiribati	0.5<br>(0.1, 0.9)	0.8<br>(0.2, 1.4)	-0.50<br>(-0.86, -0.05)	-0.80<br>(-0.95, -0.72)	0.022<br>(-0.030, 0.073)	0.401	14.8<br>(2.7, 26.2)	19.3<br>(3.9, 33.6)	-0.53<br>(-0.86, -0.15)	-0.80<br>(-0.94, -0.73)	0.432<br>(-0.807, 1.672)	0.482
Kuwait	636.2<br>(341.3, 944.5)	26.0<br>(14.4, 38.2)	-0.23<br>(-0.37, -0.07)	0.08<br>(-0.08, 0.25)	**-0.242<br>(-0.428, -0.056)**	**0.012**	15712.3<br>(8495.0, 23594.0)	493.8<br>(270.4, 732.3)	-0.30<br>(-0.44, -0.15)	0.08<br>(-0.10, 0.26)	**-6.368<br>(-10.129, -2.607)**	**0.002**
Kyrgyzstan	41.6<br>(5.7, 129.8)	1.1<br>(0.1, 3.6)	0.16<br>(-0.90, 2.17)	-0.05<br>(-0.37, 0.07)	**0.011<br>(0.001, 0.021)**	**0.037**	872.7<br>(133.2, 2557.3)	19.8<br>(2.8, 60.1)	0.05<br>(-0.53, 2.41)	-0.04<br>(-0.36, 0.08)	0.094<br>(-0.111, 0.299)	0.357
Lao People's Democratic Republic	165.0<br>(84.1, 268.6)	4.2<br>(2.2, 6.9)	1.30<br>(-14.28, 17.28)	-0.25<br>(-0.30, -0.15)	**0.074<br>(0.029, 0.118)**	**0.002**	4310.4<br>(2201.3, 7014.7)	89.7<br>(45.6, 145.4)	1.03<br>(-12.48, 12.59)	-0.25<br>(-0.30, -0.15)	**1.382<br>(0.414, 2.349)**	**0.007**
Latvia	3.0<br>(-1.3, 11.1)	0.1<br>(-0.0, 0.3)	-8.04<br>(-14997.88, 2465.66)	366.49<br>(-82.07, 152.50)	**0.002<br>(0.001, 0.003)**	**<0.001**	64.6<br>(-25.8, 206.7)	2.0<br>(-0.9, 6.3)	-11.47<br>(-28232.25, 4300.78)	13.75<br>(-118.15, 72.54)	**0.047<br>(0.026, 0.069)**	**<0.001**
Lebanon	35.0<br>(2.5, 93.7)	0.5<br>(0.0, 1.4)	3.43<br>(-46.91, 48.91)	0.33<br>(-1.49, 2.55)	0.007<br>(-0.001, 0.015)	0.089	604.8<br>(48.6, 1626.7)	9.8<br>(0.8, 26.2)	2.67<br>(-51.11, 43.45)	0.33<br>(-1.29, 2.72)	0.104<br>(-0.044, 0.251)	0.161
Lesotho	0.0<br>(-0.1, 0.2)	0.0<br>(-0.0, 0.0)	-0.19<br>(-13.77, 31.00)	-1.00<br>(-1.19, -0.83)	**0.002<br>(0.000, 0.004)**	**0.015**	0.1<br>(-3.2, 4.0)	0.0<br>(-0.3, 0.4)	0.01<br>(-15.91, 30.83)	-1.00<br>(-1.11, -0.79)	**0.050<br>(0.011, 0.088)**	**0.014**
Liberia	-0.7<br>(-16.1, 14.7)	-0.1<br>(-1.0, 0.8)	-0.82<br>(-5.05, 1.24)	-1.24<br>(-7.68, 12.70)	0.030<br>(0.000, 0.059)	0.050	-4.2<br>(-433.7, 422.8)	-0.8<br>(-18.8, 17.3)	-0.88<br>(-5.64, 1.25)	-1.13<br>(-13.85, 10.76)	**0.626<br>(0.005, 1.247)**	**0.048**
Libya	432.8<br>(175.2, 836.5)	9.6<br>(3.9, 18.5)	1.24<br>(0.60, 5.31)	0.31<br>(0.18, 0.60)	**0.127<br>(0.090, 0.164)**	**<0.001**	10833.1<br>(4371.0, 20852.2)	202.7<br>(82.0, 385.4)	1.16<br>(0.56, 4.33)	0.30<br>(0.17, 0.58)	**2.498<br>(1.701, 3.295)**	**<0.001**
Lithuania	6.4<br>(-0.4, 27.9)	0.1<br>(-0.0, 0.4)	-4.95<br>(-1629.47, 32.13)	1.31<br>(-2.57, 7.10)	**0.003<br>(0.002, 0.005)**	**<0.001**	99.0<br>(-9.2, 406.1)	1.7<br>(-0.2, 6.5)	-4.05<br>(-1700.07, 13.39)	1.48<br>(-5.89, 8.49)	**0.070<br>(0.047, 0.092)**	**<0.001**
Luxembourg	0.2<br>(-0.0, 1.1)	0.0<br>(-0.0, 0.1)	-0.88<br>(-0.99, -0.62)	-0.86<br>(-1.00, -0.75)	-0.003<br>(-0.006, 0.000)	0.062	3.5<br>(0.0, 16.9)	0.3<br>(0.0, 1.5)	-0.89<br>(-0.99, -0.73)	-0.85<br>(-0.99, -0.74)	**-0.054<br>(-0.102, -0.006)**	**0.028**
Madagascar	84.7<br>(3.4, 197.3)	0.9<br>(-0.0, 2.1)	-13.86<br>(-26.58, 59.92)	0.08<br>(-0.46, 0.43)	**0.023<br>(0.018, 0.028)**	**<0.001**	2614.6<br>(163.2, 6033.1)	20.0<br>(0.7, 46.7)	-22.10<br>(-36.46, 25.20)	0.07<br>(-0.33, 0.40)	**0.485<br>(0.375, 0.595)**	**<0.001**
Malawi	134.9<br>(-4.5, 302.8)	2.1<br>(-0.1, 4.9)	3.86<br>(-23.47, 26.88)	0.54<br>(-0.40, 1.76)	**0.030<br>(0.014, 0.046)**	**<0.001**	3804.0<br>(29.3, 8126.4)	45.7<br>(-1.5, 102.5)	3.48<br>(-26.53, 33.15)	0.53<br>(-0.47, 1.45)	**0.619<br>(0.274, 0.964)**	**<0.001**
Malaysia	445.2<br>(375.6, 522.7)	1.8<br>(1.5, 2.1)	0.76<br>(0.39, 2.28)	-0.37<br>(-0.43, -0.29)	**0.052<br>(0.025, 0.079)**	**<0.001**	10300.7<br>(8801.1, 12103.7)	36.4<br>(31.0, 42.8)	0.63<br>(0.30, 1.90)	-0.37<br>(-0.44, -0.29)	**1.029<br>(0.473, 1.585)**	**<0.001**

Maldives	4.1 (2.8, 5.4)	1.4 (1.0, 1.9)	-0.33 (-0.49, 0.33)	-0.29 (-0.40, -0.19)	-0.006 (-0.030, 0.017)	0.594	90.4 (63.4, 119.6)	26.1 (18.3, 34.7)	-0.46 (-0.60, 0.02)	-0.29 (-0.40, -0.18)	-0.413 (-0.903, 0.076)	0.095
Mali	1895.8 (1350.6, 2591.2)	25.7 (18.7, 35.1)	0.15 (-0.05, 0.41)	0.10 (0.04, 0.18)	**0.208 (0.117, 0.298)**	**<0.001**	52126.6 (36521.8, 71913.4)	533.0 (379.8, 728.9)	0.10 (-0.11, 0.39)	0.10 (0.04, 0.18)	**3.639 (1.716, 5.562)**	**<0.001**
Malta	6.9 (0.2, 20.7)	0.6 (0.0, 1.9)	0.10 (-15.52, 24.21)	0.67 (-6.12, 4.95)	-0.003 (-0.011, 0.004)	0.324	103.8 (3.3, 314.5)	10.7 (0.3, 32.1)	0.07 (-37.94, 15.82)	0.65 (-4.91, 7.19)	-0.066 (-0.188, 0.055)	0.272
Marshall Islands	1.8 (1.2, 2.6)	6.1 (4.1, 8.4)	2.57 (1.09, 11.02)	0.15 (0.09, 0.23)	**0.151 (0.120, 0.182)**	**<0.001**	55.6 (35.6, 81.1)	144.0 (95.6, 203.4)	2.65 (1.11, 11.06)	0.15 (0.08, 0.23)	**3.579 (2.858, 4.300)**	**<0.001**
Mauritania	744.8 (486.6, 1055.1)	40.4 (26.5, 57.5)	-0.05 (-0.26, 0.22)	0.24 (0.16, 0.34)	-0.049 (-0.178, 0.079)	0.437	17110.5 (11230.4, 24109.6)	784.0 (511.2, 1109.8)	-0.12 (-0.32, 0.14)	0.24 (0.16, 0.34)	**-2.785 (-5.380, -0.190)**	**0.036**
Mauritius	5.3 (-0.6, 13.6)	0.3 (-0.0, 0.8)	3.84 (-106.82, 170.63)	-0.63 (-0.92, -0.52)	**0.011 (0.003, 0.018)**	**0.006**	125.5 (-17.0, 316.1)	7.0 (-0.8, 17.6)	3.95 (-80.48, 264.09)	-0.63 (-0.91, -0.54)	**0.255 (0.089, 0.422)**	**0.004**
Mexico	2106.4 (953.6, 3392.2)	1.8 (0.8, 2.9)	0.98 (-2.87, 6.68)	-0.17 (-0.25, -0.08)	**0.028 (0.018, 0.037)**	**<0.001**	48644.7 (22536.2, 77277.8)	38.1 (17.6, 60.7)	1.00 (0.38, 6.15)	-0.15 (-0.25, -0.05)	**0.602 (0.403, 0.800)**	**<0.001**
Micronesia (Federated States of)	3.0 (2.1, 4.3)	4.7 (3.2, 6.5)	1.89 (0.50, 12.97)	0.09 (0.03, 0.17)	**0.112 (0.081, 0.143)**	**<0.001**	85.1 (57.6, 124.3)	108.3 (73.9, 155.5)	1.82 (0.61, 15.25)	0.09 (0.02, 0.17)	**2.554 (1.845, 3.264)**	**<0.001**
Monaco	0.0 (-0.0, 0.2)	0.0 (-0.0, 0.1)	-0.15 (-4.93, 4.96)	-0.67 (-1.05, -0.45)	0.000 (-0.001, 0.001)	0.851	0.5 (-0.0, 2.4)	0.5 (-0.0, 2.5)	-0.18 (-2.73, 4.07)	-0.66 (-1.01, -0.51)	0.002 (-0.023, 0.026)	0.902
Mongolia	0.4 (-1.6, 2.8)	0.0 (-0.1, 0.2)	-5.95 (-15.50, 16.70)	-0.40 (-1.55, 0.97)	0.001 (-0.001, 0.003)	0.211	6.0 (-54.5, 61.8)	0.4 (-1.8, 3.0)	-2.53 (-17.08, 18.97)	-0.44 (-2.63, 0.63)	0.024 (-0.016, 0.064)	0.233
Montenegro	9.7 (2.5, 26.2)	1.1 (0.3, 3.1)	8.97 (-50.21, 90.47)	0.34 (0.11, 1.05)	**0.023 (0.014, 0.032)**	**<0.001**	171.7 (45.8, 451.6)	19.0 (5.1, 49.7)	7.29 (-31.12, 82.61)	0.30 (0.08, 0.96)	**0.364 (0.203, 0.526)**	**<0.001**
Morocco	1098.0 (496.2, 2147.3)	3.7 (1.7, 7.4)	0.44 (0.11, 2.21)	0.36 (0.20, 0.72)	**0.061 (0.029, 0.093)**	**<0.001**	23727.4 (10550.9, 46452.7)	71.6 (32.0, 140.3)	0.30 (-0.03, 1.81)	0.36 (0.20, 0.72)	**1.005 (0.367, 1.643)**	**0.003**
Mozambique	288.6 (-51.4, 675.0)	2.9 (-0.7, 7.0)	3.72 (-11.74, 21.25)	0.35 (-1.84, 3.56)	**0.053 (0.030, 0.075)**	**<0.001**	8531.0 (-900.7, 19310.8)	66.3 (-12.2, 155.8)	3.25 (-14.98, 18.42)	0.32 (-2.62, 2.78)	**1.212 (0.711, 1.713)**	**<0.001**
Myanmar	4979.7 (3560.4, 6725.0)	11.8 (8.5, 15.8)	0.40 (0.03, 0.93)	-0.10 (-0.15, -0.04)	-0.035 (-0.234, 0.164)	0.721	117654.3 (82505.5, 160083.6)	244.3 (172.8, 330.9)	0.25 (-0.09, 0.82)	-0.11 (-0.16, -0.04)	-1.679 (-5.964, 2.607)	0.430
Namibia	24.2 (-3.4, 58.6)	2.2 (-0.4, 5.3)	0.46 (-0.78, 2.12)	-0.66 (-1.26, -0.60)	**0.074 (0.025, 0.122)**	**0.004**	583.7 (-43.4, 1372.1)	43.1 (-5.3, 103.6)	0.43 (-0.98, 1.74)	-0.66 (-1.17, -0.60)	**1.407 (0.445, 2.368)**	**0.006**
Nauru	0.4 (0.3, 0.5)	6.9 (5.0, 9.3)	0.84 (0.40, 1.67)	0.05 (-0.00, 0.10)	**0.169 (0.122, 0.216)**	**<0.001**	10.9 (7.7, 14.9)	168.3 (120.1, 227.9)	0.86 (0.38, 1.75)	0.05 (-0.01, 0.11)	**4.122 (2.975, 5.269)**	**<0.001**

Nepal	565.8<br>(77.5, 1245.1)	2.9<br>(0.3, 6.3)	0.49<br>(-4.59, 4.94)	-0.46<br>(-0.78, -0.37)	**0.045<br>(0.010, 0.080)**	**0.013**	12871.8<br>(2144.5, 27573.6)	56.5<br>(8.5, 123.3)	0.35<br>(-3.87, 4.67)	-0.46<br>(-0.73, -0.38)	0.711<br>(-0.012, 1.433)	0.054
Netherlands	7.6<br>(0.4, 36.5)	0.0<br>(0.0, 0.1)	-0.86<br>(-0.96, -0.67)	-0.84<br>(-0.96, -0.73)	**-0.003<br>(-0.005, -0.001)**	**0.009**	111.5<br>(5.6, 521.8)	0.3<br>(0.0, 1.4)	-0.89<br>(-0.97, -0.70)	-0.84<br>(-0.96, -0.73)	**-0.067<br>(-0.108, -0.026)**	**0.002**
New Zealand	0.5<br>(0.0, 2.2)	0.0<br>(0.0, 0.0)	-0.82<br>(-7.14, 2.11)	-0.58<br>(-5.72, 6.32)	0.000<br>(-0.001, 0.000)	0.188	7.5<br>(0.4, 33.0)	0.1<br>(0.0, 0.4)	-0.85<br>(-4.31, 1.72)	-0.58<br>(-4.76, 8.06)	-0.005<br>(-0.011, 0.001)	0.113
Nicaragua	62.8<br>(53.3, 74.4)	1.4<br>(1.2, 1.7)	16.34<br>(-43.45, 33.71)	-0.44<br>(-0.52, -0.34)	**0.100<br>(0.030, 0.169)**	**0.006**	1475.1<br>(1254.7, 1751.1)	29.6<br>(25.2, 35.1)	8.16<br>(-28.80, 101.82)	-0.43<br>(-0.52, -0.32)	**2.018<br>(0.584, 3.451)**	**0.007**
Niger	1698.0<br>(1105.7, 2435.1)	25.7<br>(17.4, 35.9)	0.22<br>(-0.05, 1.22)	0.01<br>(-0.04, 0.06)	**0.222<br>(0.143, 0.302)**	**<0.001**	45755.2<br>(29236.3, 67783.7)	513.0<br>(334.8, 732.6)	0.14<br>(-0.13, 1.05)	0.01<br>(-0.05, 0.06)	**3.470<br>(1.841, 5.098)**	**<0.001**
Nigeria	4705.4<br>(2819.3, 6526.5)	6.2<br>(3.8, 8.5)	0.18<br>(-0.21, 2.61)	0.12<br>(-0.08, 0.43)	**0.043<br>(0.000, 0.085)**	**0.048**	122508.5<br>(71998.1, 175728.4)	124.0<br>(74.2, 172.9)	0.09<br>(-0.28, 2.85)	0.11<br>(-0.13, 0.46)	0.592<br>(-0.253, 1.437)	0.163
Niue	0.0<br>(0.0, 0.0)	0.8<br>(0.6, 0.9)	2.44<br>(0.78, 11.83)	-0.44<br>(-0.48, -0.39)	**0.022<br>(0.012, 0.032)**	**<0.001**	0.4<br>(0.3, 0.4)	16.4<br>(12.4, 21.1)	2.38<br>(0.77, 10.03)	-0.43<br>(-0.48, -0.38)	**0.465<br>(0.254, 0.675)**	**<0.001**
North Macedonia	51.6<br>(10.3, 138.4)	2.1<br>(0.4, 5.7)	1.85<br>(0.88, 17.54)	0.13<br>(-0.06, 1.09)	0.026<br>(-0.001, 0.054)	0.059	946.7<br>(199.6, 2502.0)	33.2<br>(6.8, 88.7)	1.29<br>(0.52, 14.36)	0.16<br>(-0.06, 1.08)	0.286<br>(-0.145, 0.717)	0.186
Northern Mariana Islands	0.9<br>(0.8, 1.1)	2.2<br>(1.9, 2.5)	-3.37<br>(-3.10, 19.36)	-0.06<br>(-0.21, 0.13)	**0.099<br>(0.086, 0.113)**	**<0.001**	24.4<br>(21.1, 27.1)	46.4<br>(40.5, 51.5)	-3.30<br>(-2.75, 16.41)	-0.06<br>(-0.22, 0.12)	**2.110<br>(1.823, 2.397)**	**<0.001**
Norway	-0.0<br>(-0.4, 0.3)	-0.0<br>(-0.0, 0.0)	-1.23<br>(-5.47, 14.65)	-1.08<br>(-4.81, 3.13)	0.000<br>(0.000, 0.000)	0.143	-0.1<br>(-4.6, 4.2)	0.0<br>(-0.0, 0.0)	-0.97<br>(-12.91, 21.95)	-0.98<br>(-4.18, 1.45)	**-0.001<br>(-0.003, 0.000)**	**0.05**
Oman	549.2<br>(372.2, 772.6)	38.5<br>(26.2, 53.6)	-0.08<br>(-0.30, 0.24)	-0.04<br>(-0.10, 0.03)	**0.292<br>(0.132, 0.452)**	**<0.001**	13947.1<br>(9391.4, 19707.0)	711.7<br>(484.0, 1000.1)	-0.20<br>(-0.41, 0.11)	-0.06<br>(-0.14, 0.00)	1.756<br>(-1.394, 4.906)	0.264
Pakistan	27731.3<br>(15670.9, 42098.6)	27.1<br>(15.3, 41.2)	0.18<br>(-0.05, 0.51)	-0.03<br>(-0.22, 0.24)	**0.188<br>(0.081, 0.295)**	**0.001**	735019.1<br>(409380.7, 1118310.6)	569.3<br>(320.8, 863.8)	0.17<br>(-0.07, 0.51)	-0.03<br>(-0.24, 0.26)	**3.814<br>(1.511, 6.117)**	**0.002**
Palau	0.5<br>(0.4, 0.7)	2.8<br>(1.9, 3.7)	1.47<br>(0.57, 6.03)	0.30<br>(0.18, 0.51)	**0.062<br>(0.047, 0.077)**	**<0.001**	13.7<br>(9.4, 18.2)	60.6<br>(41.5, 81.1)	1.39<br>(0.50, 4.82)	0.30<br>(0.17, 0.51)	**1.322<br>(0.988, 1.656)**	**<0.001**
Palestine	123.8<br>(37.3, 261.8)	6.7<br>(1.9, 14.4)	1.48<br>(-6.87, 13.01)	0.13<br>(0.03, 0.42)	**0.097<br>(0.039, 0.155)**	**0.002**	2825.0<br>(883.8, 5931.8)	118.0<br>(35.9, 249.4)	1.36<br>(-8.52, 11.27)	0.12<br>(0.02, 0.40)	**1.596<br>(0.571, 2.621)**	**0.003**
Panama	7.5<br>(-8.6, 12.5)	0.2<br>(-0.2, 0.3)	-1.36<br>(-1.87, 8.57)	-0.61<br>(-3.31, 0.94)	**0.028<br>(0.021, 0.035)**	**<0.001**	157.3<br>(-147.9, 254.5)	3.5<br>(-3.3, 5.7)	-1.41<br>(-1.90, 6.34)	-0.60<br>(-2.99, -0.40)	**0.548<br>(0.411, 0.685)**	**<0.001**
Papua New Guinea	20.2<br>(6.5, 30.6)	0.5<br>(0.1, 0.7)	-87.62<br>(-40.02, 29.21)	0.71<br>(-0.67, 3.90)	**0.014<br>(0.009, 0.019)**	**<0.001**	582.5<br>(170.9, 882.5)	10.6<br>(3.3, 16.1)	81.85<br>(-17.47, 27.39)	0.69<br>(-1.02, 2.92)	**0.301<br>(0.194, 0.408)**	**<0.001**

Paraguay	128.6<br>(11.1, 282.3)	2.3<br>(0.2, 5.1)	-0.09<br>(-0.83, 1.03)	-0.47<br>(-0.77, -0.31)	**0.044<br>(0.021, 0.067)**	**<0.001**	2802.2<br>(265.5, 6076.7)	47.9<br>(4.6, 104.2)	-0.08<br>(-0.94, 0.98)	-0.45<br>(-0.77, -0.28)	**0.850<br>(0.383, 1.316)**	**<0.001**
Peru	12.9<br>(-0.7, 26.6)	0.0<br>(-0.0, 0.1)	-1.91<br>(-13.34, 10.92)	-0.03<br>(-4.60, 4.47)	**0.003<br>(0.002, 0.003)**	**<0.001**	299.3<br>(21.5, 588.6)	0.9<br>(0.1, 1.7)	-2.16<br>(-18.83, 11.34)	-0.05<br>(-4.13, 3.97)	**0.056<br>(0.042, 0.070)**	**<0.001**
Philippines	2521.7<br>(2135.4, 2928.2)	3.4<br>(2.9, 3.9)	3.83<br>(-13.69, 16.72)	-0.17<br>(-0.29, -0.03)	**0.097<br>(0.067, 0.126)**	**<0.001**	66676.9<br>(55996.6, 77357.8)	77.1<br>(65.0, 89.3)	4.15<br>(-17.96, 16.46)	-0.17<br>(-0.30, -0.02)	**2.295<br>(1.645, 2.946)**	**<0.001**
Poland	96.0<br>(6.9, 362.7)	0.1<br>(0.0, 0.5)	1.01<br>(-34.85, 28.49)	-0.41<br>(-0.66, -0.32)	0.000<br>(-0.004, 0.003)	0.787	1535.6<br>(129.5, 5671.8)	2.1<br>(0.2, 7.7)	0.62<br>(-27.49, 24.02)	-0.42<br>(-0.65, -0.33)	-0.025<br>(-0.088, 0.037)	0.420
Portugal	7.9<br>(2.7, 20.8)	0.0<br>(0.0, 0.1)	-0.92<br>(-0.95, -0.89)	0.19<br>(-0.02, 0.99)	**-0.005<br>(-0.009, -0.002)**	**0.002**	116.1<br>(38.5, 309.5)	0.5<br>(0.1, 1.3)	-0.92<br>(-0.95, -0.89)	0.15<br>(-0.06, 0.94)	**-0.096<br>(-0.153, -0.038)**	**0.002**
Puerto Rico	51.3<br>(38.5, 63.7)	0.6<br>(0.5, 0.8)	0.01<br>(-0.19, 0.19)	-0.13<br>(-0.26, 0.01)	0.003<br>(-0.006, 0.013)	0.462	924.9<br>(703.2, 1129.7)	14.1<br>(10.8, 17.1)	0.12<br>(-0.11, 0.33)	-0.13<br>(-0.27, 0.02)	0.091<br>(-0.096, 0.277)	0.329
Qatar	176.8<br>(107.4, 263.9)	35.6<br>(22.1, 51.8)	-0.46<br>(-0.59, -0.28)	0.15<br>(0.07, 0.24)	**-1.214<br>(-1.704, -0.724)**	**<0.001**	5114.6<br>(3060.3, 7714.8)	594.9<br>(367.8, 873.2)	-0.49<br>(-0.63, -0.31)	0.07<br>(-0.01, 0.16)	**-22.403<br>(-30.536, -14.270)**	**<0.001**
Republic of Korea	528.2<br>(88.3, 1360.6)	0.6<br>(0.1, 1.5)	-0.55<br>(-3.14, 1.90)	0.11<br>(0.06, 0.28)	**-0.019<br>(-0.031, -0.007)**	**0.004**	8359.2<br>(1561.1, 21269.2)	9.3<br>(1.7, 23.5)	-0.61<br>(-2.59, 1.84)	0.10<br>(0.04, 0.27)	**-0.392<br>(-0.613, -0.172)**	**0.001**
Republic of Moldova	80.6<br>(8.2, 272.3)	1.3<br>(0.1, 4.5)	-0.11<br>(-3.78, 11.22)	-0.28<br>(-0.35, -0.22)	-0.004<br>(-0.035, 0.026)	0.765	1512.0<br>(169.4, 4998.9)	25.6<br>(2.9, 84.3)	0.02<br>(-7.78, 12.91)	-0.28<br>(-0.35, -0.22)	0.113<br>(-0.466, 0.693)	0.692
Romania	531.6<br>(92.7, 1409.3)	1.3<br>(0.2, 3.5)	0.31<br>(-0.01, 3.45)	0.06<br>(-0.08, 0.82)	-0.002<br>(-0.019, 0.015)	0.803	8905.4<br>(1664.3, 23104.3)	24.0<br>(4.7, 61.5)	0.37<br>(0.02, 3.61)	0.06<br>(-0.09, 0.77)	-0.037<br>(-0.347, 0.273)	0.811
Russian Federation	1557.7<br>(161.5, 4503.7)	0.7<br>(0.1, 1.9)	3.34<br>(-35.08, 30.13)	0.71<br>(0.40, 1.53)	**0.013<br>(0.003, 0.022)**	**0.009**	31326.5<br>(4405.2, 86034.3)	13.8<br>(1.9, 36.9)	4.01<br>(-52.61, 39.44)	0.79<br>(0.34, 1.58)	**0.265<br>(0.056, 0.474)**	**0.015**
Rwanda	0.4<br>(-0.5, 1.8)	0.0<br>(-0.0, 0.0)	NA	0.05<br>(-1.46, 1.26)	0.000<br>(0.000, 0.000)	0.051	11.1<br>(-11.6, 45.5)	0.2<br>(-0.2, 0.7)	NA	0.03<br>(-1.53, 1.36)	0.004<br>(-0.001, 0.008)	0.088
Saint Kitts and Nevis	0.5<br>(0.3, 0.6)	0.8<br>(0.6, 1.1)	-0.27<br>(-0.39, -0.12)	-0.24<br>(-0.32, -0.18)	-0.002<br>(-0.017, 0.013)	0.804	10.4<br>(7.0, 14.1)	15.8<br>(10.7, 21.4)	-0.32<br>(-0.44, -0.16)	-0.24<br>(-0.32, -0.17)	-0.087<br>(-0.368, 0.195)	0.534
Saint Lucia	1.9<br>(1.2, 2.6)	0.8<br>(0.5, 1.1)	-0.05<br>(-5.73, 4.47)	-0.07<br>(-0.18, 0.01)	0.005<br>(-0.007, 0.018)	0.404	36.9<br>(23.5, 49.9)	15.6<br>(9.6, 21.1)	-0.10<br>(-8.47, 5.51)	-0.09<br>(-0.21, -0.01)	0.081<br>(-0.158, 0.319)	0.496
Saint Vincent and the Grenadines	1.3<br>(1.0, 1.7)	1.0<br>(0.7, 1.3)	0.31<br>(-4.99, 0.44)	-0.20<br>(-0.27, -0.14)	**0.016<br>(0.001, 0.030)**	**0.035**	29.1<br>(21.2, 37.6)	20.8<br>(15.2, 27.0)	0.32<br>(-6.27, 2.93)	-0.19<br>(-0.26, -0.13)	0.302<br>(0.005, 0.599)	**0.046**
Samoa	0.1<br>(0.0, 0.3)	0.1<br>(0.0, 0.2)	-3.35<br>(-1.96, 2.26)	-0.71<br>(-0.92, -0.58)	0.005<br>(-0.008, 0.019)	0.425	3.5<br>(0.8, 6.8)	2.3<br>(0.4, 4.6)	-4.44<br>(-1.98, 1.23)	-0.70<br>(-0.88, -0.59)	0.123<br>(-0.169, 0.415)	0.397

San Marino	0.2<br>(0.0, 0.4)	0.2<br>(0.0, 0.4)	0.37<br>(-0.30, 8.16)	-0.33<br>(-0.47, -0.12)	0.000<br>(-0.004, 0.004)	0.837	2.1<br>(0.5, 6.2)	2.6<br>(0.6, 7.5)	0.34<br>(-0.27, 7.60)	-0.31<br>(-0.47, -0.09)	-0.009<br>(-0.073, 0.055)	0.775
Sao Tome and Principe	0.3<br>(-0.1, 0.7)	0.3<br>(-0.1, 0.7)	11.53<br>(-2.45, 2.09)	-0.38<br>(-1.04, 0.13)	**0.011<br>(0.002, 0.021)**	**0.023**	7.5<br>(-2.0, 17.7)	6.2<br>(-2.0, 15.1)	5.26<br>(-2.50, 1.57)	-0.36<br>(-0.99, -0.06)	**0.211<br>(0.008, 0.413)**	**0.042**
Saudi Arabia	6649.4<br>(3659.9, 9866.4)	42.9<br>(25.1, 62.1)	0.26<br>(-0.02, 0.65)	0.03<br>(-0.01, 0.08)	**0.233<br>(0.082, 0.384)**	**0.004**	209176.9<br>(113850.5, 314090.3)	893.9<br>(504.5, 1299.7)	0.27<br>(-0.05, 0.71)	0.03<br>(-0.02, 0.08)	**5.854<br>(2.819, 8.889)**	**<0.001**
Senegal	1203.0<br>(861.4, 1595.3)	18.4<br>(13.2, 24.3)	0.25<br>(-0.09, 1.03)	0.15<br>(0.07, 0.29)	**0.171<br>(0.104, 0.237)**	**<0.001**	28787.0<br>(20275.0, 38392.8)	364.5<br>(259.7, 484.3)	0.17<br>(-0.16, 0.85)	0.14<br>(0.07, 0.28)	**2.887<br>(1.506, 4.269)**	**<0.001**
Serbia	347.7<br>(72.1, 880.7)	2.0<br>(0.4, 5.1)	0.55<br>(0.05, 6.83)	0.23<br>(0.03, 1.29)	-0.011<br>(-0.038, 0.017)	0.435	5613.0<br>(1252.7, 13970.8)	33.6<br>(7.6, 83.6)	0.56<br>(0.03, 6.58)	0.23<br>(0.03, 1.26)	-0.147<br>(-0.609, 0.315)	0.521
Seychelles	0.6<br>(0.4, 0.8)	0.5<br>(0.4, 0.7)	0.24<br>(-0.07, 1.00)	-0.49<br>(-0.56, -0.42)	**0.015<br>(0.006, 0.025)**	**0.003**	13.4<br>(9.2, 17.6)	11.5<br>(7.8, 15.2)	0.12<br>(-0.15, 0.71)	-0.49<br>(-0.56, -0.43)	**0.297<br>(0.090, 0.504)**	**0.006**
Sierra Leone	58.4<br>(18.9, 95.6)	1.8<br>(0.6, 2.9)	1.47<br>(-5.48, 6.36)	-0.26<br>(-0.52, -0.01)	**0.046<br>(0.024, 0.068)**	**<0.001**	1558.9<br>(503.6, 2609.4)	37.2<br>(12.0, 61.2)	1.32<br>(-6.17, 5.50)	-0.26<br>(-0.52, -0.02)	**0.957<br>(0.491, 1.422)**	**<0.001**
Singapore	36.3<br>(23.7, 41.2)	0.4<br>(0.3, 0.5)	-0.58<br>(-0.74, -0.21)	-0.29<br>(-0.38, -0.26)	-0.009<br>(-0.020, 0.001)	0.087	680.7<br>(422.4, 769.4)	8.0<br>(4.9, 9.1)	-0.58<br>(-0.75, -0.16)	-0.30<br>(-0.42, -0.27)	-0.162<br>(-0.363, 0.038)	0.109
Slovakia	52.6<br>(6.4, 174.8)	0.6<br>(0.1, 1.8)	0.46<br>(0.16, 4.36)	-0.05<br>(-0.14, 0.30)	0.002<br>(-0.005, 0.008)	0.581	864.4<br>(112.3, 2825.2)	9.2<br>(1.2, 29.8)	0.26<br>(0.01, 3.75)	-0.04<br>(-0.15, 0.30)	-0.022<br>(-0.138, 0.095)	0.709
Slovenia	10.0<br>(1.2, 30.4)	0.2<br>(0.0, 0.6)	0.35<br>(-12.47, 22.27)	-0.15<br>(-0.24, 0.01)	-0.004<br>(-0.009, 0.001)	0.099	144.2<br>(20.0, 442.9)	3.0<br>(0.4, 9.1)	0.22<br>(-13.46, 15.89)	-0.16<br>(-0.26, 0.01)	**-0.087<br>(-0.173, -0.001)**	**0.049**
Solomon Islands	3.0<br>(-1.5, 5.8)	0.9<br>(-0.7, 1.8)	84.11<br>(-11.23, 17.97)	0.24<br>(-1.98, 0.97)	**0.034<br>(0.027, 0.041)**	**<0.001**	89.7<br>(-38.1, 168.1)	22.9<br>(-12.0, 43.9)	19.89<br>(-5.22, 16.70)	0.21<br>(-2.14, 1.08)	**0.823<br>(0.660, 0.986)**	**<0.001**
Somalia	79.4<br>(-167.4, 244.3)	1.6<br>(-3.2, 4.8)	0.15<br>(-7.21, 7.48)	-0.43<br>(-2.98, 1.55)	**0.079<br>(0.035, 0.124)**	**0.001**	2194.4<br>(-5960.6, 7464.7)	35.3<br>(-71.8, 106.7)	0.11<br>(-9.15, 7.19)	-0.42<br>(-2.92, 2.17)	**1.723<br>(0.737, 2.710)**	**0.001**
South Africa	96.5<br>(-34.5, 278.7)	0.2<br>(-0.1, 0.7)	1.27<br>(-10.61, 8.24)	-0.62<br>(-2.30, -0.20)	**0.014<br>(0.007, 0.021)**	**<0.001**	2231.2<br>(-663.3, 6137.7)	4.9<br>(-1.6, 13.8)	0.99<br>(-5.52, 9.22)	-0.62<br>(-1.56, -0.39)	**0.265<br>(0.135, 0.396)**	**<0.001**
South Sudan	686.4<br>(474.6, 958.6)	21.4<br>(14.9, 29.3)	0.67<br>(0.22, 1.41)	0.01<br>(-0.06, 0.12)	**0.356<br>(0.239, 0.472)**	**<0.001**	19448.7<br>(13130.6, 27633.8)	456.6<br>(316.5, 636.7)	0.67<br>(0.18, 1.47)	0.02<br>(-0.06, 0.13)	**7.486<br>(4.964, 10.009)**	**<0.001**
Spain	266.5<br>(111.3, 557.3)	0.2<br>(0.1, 0.4)	-0.58<br>(-0.69, -0.37)	-0.31<br>(-0.35, -0.28)	**-0.006<br>(-0.011, -0.002)**	**0.008**	3743.9<br>(1477.6, 8043.9)	3.6<br>(1.4, 7.8)	-0.59<br>(-0.68, -0.41)	-0.31<br>(-0.34, -0.28)	**-0.114<br>(-0.191, -0.038)**	**0.005**
Sri Lanka	413.9<br>(276.0, 549.0)	1.7<br>(1.2, 2.3)	0.04<br>(-2.59, 4.96)	-0.48<br>(-0.63, -0.34)	0.056<br>(-0.047, 0.159)	0.274	8205.9<br>(5405.5, 11105.6)	31.5<br>(20.8, 42.4)	0.10<br>(-6.74, 1.11)	-0.48<br>(-0.64, -0.33)	1.053<br>(-0.973, 3.079)	0.297

Sudan	7817.4 (5114.4, 11677.3)	47.0 (30.9, 69.2)	-0.15 (-0.33, 0.13)	0.20 (0.12, 0.28)	-0.019 (-0.200, 0.163)	0.834	199083.6 (127032.3, 306226.4)	953.9 (618.9, 1436.3)	-0.22 (-0.41, 0.06)	0.19 (0.10, 0.27)	-2.904 (-6.771, 0.964)	0.136
Suriname	4.1 (-0.9, 6.3)	0.7 (-0.2, 1.0)	1.33 (-4.05, 0.15)	-0.57 (-1.27, -0.42)	**0.038 (0.025, 0.051)**	**<0.001**	97.8 (-18.1, 148.0)	15.2 (-3.1, 23.1)	1.24 (-4.81, 0.17)	-0.57 (-1.22, -0.42)	**0.829 (0.543, 1.115)**	**<0.001**
Sweden	1.0 (-0.7, 5.6)	0.0 (-0.0, 0.0)	0.65 (-37.80, 66.78)	-0.02 (-1.75, 1.00)	0.000 (0.000, 0.000)	0.446	15.2 (-8.5, 80.9)	0.1 (-0.0, 0.4)	0.43 (-42.27, 51.31)	-0.04 (-1.55, 1.07)	-0.002 (-0.006, 0.002)	0.364
Switzerland	4.2 (0.1, 18.7)	0.0 (0.0, 0.1)	-0.72 (-3.16, 2.03)	-0.74 (-0.96, -0.64)	-0.002 (-0.003, 0.000)	0.076	57.2 (1.5, 253.7)	0.3 (0.0, 1.3)	-0.75 (-2.33, 2.09)	-0.74 (-0.96, -0.64)	**-0.030 (-0.057, -0.002)**	**0.035**
Syrian Arab Republic	1011.2 (452.9, 1997.8)	10.1 (4.6, 19.6)	0.63 (0.24, 1.42)	0.02 (-0.08, 0.15)	**0.122 (0.061, 0.182)**	**<0.001**	23434.7 (10312.4, 46619.0)	189.8 (84.9, 374.4)	0.43 (0.03, 1.18)	0.02 (-0.10, 0.16)	**1.654 (0.447, 2.861)**	**0.009**
Taiwan (Province of China)	787.1 (53.8, 1596.6)	1.8 (0.1, 3.7)	-0.15 (-4.40, 4.68)	0.19 (0.10, 0.28)	**-0.018 (-0.032, -0.005)**	**0.008**	13911.0 (865.6, 28253.4)	34.0 (2.3, 68.9)	-0.18 (-3.95, 3.55)	0.18 (0.08, 0.26)	**-0.388 (-0.639, -0.137)**	**0.004**
Tajikistan	216.3 (94.9, 433.0)	5.1 (2.2, 10.4)	0.59 (0.25, 2.43)	0.10 (-0.03, 0.21)	**0.066 (0.041, 0.092)**	**<0.001**	5004.0 (2328.3, 9918.4)	93.7 (41.4, 187.0)	0.47 (0.15, 2.16)	0.10 (-0.03, 0.22)	**1.055 (0.576, 1.534)**	**<0.001**
Thailand	4232.5 (2915.9, 5570.4)	3.9 (2.7, 5.2)	0.15 (-0.28, 1.16)	-0.30 (-0.43, -0.15)	0.038 (-0.018, 0.093)	0.174	89007.3 (61623.7, 117657.3)	85.9 (59.7, 113.6)	0.17 (-0.25, 1.06)	-0.31 (-0.44, -0.15)	0.832 (-0.343, 2.007)	0.159
Timor-Leste	1.0 (-0.3, 2.4)	0.1 (-0.1, 0.3)	-1.13 (-2.71, -0.67)	-0.61 (-1.66, 0.18)	**0.036 (0.032, 0.041)**	**<0.001**	25.0 (-5.7, 57.9)	2.8 (-0.8, 6.6)	-1.14 (-3.21, -0.69)	-0.59 (-1.21, 0.31)	**0.742 (0.652, 0.832)**	**<0.001**
Togo	257.7 (144.1, 367.7)	8.6 (4.9, 12.1)	4.77 (-11.13, 2.81)	0.09 (-0.01, 0.58)	**0.220 (0.176, 0.263)**	**<0.001**	6908.4 (3749.2, 9891.4)	175.5 (98.0, 250.1)	4.51 (-10.66, 2.75)	0.08 (-0.03, 0.55)	**4.494 (3.582, 5.405)**	**<0.001**
Tokelau	0.0 (0.0, 0.0)	2.0 (1.1, 3.0)	-0.19 (-0.39, 0.13)	-0.60 (-0.73, -0.49)	**0.041 (0.000, 0.081)**	**0.048**	0.6 (0.3, 1.0)	41.8 (21.4, 64.3)	-0.21 (-0.43, 0.12)	-0.60 (-0.74, -0.49)	0.863 (-0.012, 1.737)	0.053
Tonga	1.0 (0.7, 1.4)	1.3 (0.8, 1.8)	1.91 (-10.57, 3.87)	-0.30 (-0.38, -0.24)	**0.035 (0.019, 0.050)**	**<0.001**	23.8 (15.5, 31.9)	29.4 (19.1, 39.3)	1.71 (-16.78, 2.77)	-0.30 (-0.37, -0.24)	**0.719 (0.375, 1.064)**	**<0.001**
Trinidad and Tobago	13.0 (2.2, 18.6)	0.7 (0.1, 1.0)	0.00 (-1.20, 0.41)	-0.33 (-0.76, -0.08)	0.011 (-0.002, 0.024)	0.088	284.5 (61.0, 407.3)	14.8 (2.6, 21.2)	-0.05 (-1.14, 0.36)	-0.34 (-0.72, -0.08)	0.199 (-0.083, 0.482)	0.160
Tunisia	872.7 (379.4, 1671.7)	7.5 (3.3, 14.3)	1.18 (0.31, 6.01)	0.31 (0.18, 0.49)	0.033 (-0.017, 0.083)	0.184	16929.6 (7390.0, 32750.4)	133.7 (58.6, 256.5)	1.07 (0.22, 5.59)	0.31 (0.18, 0.49)	0.468 (-0.442, 1.377)	0.302
Turkey	1357.7 (595.6, 2818.7)	1.6 (0.7, 3.4)	0.54 (0.13, 1.94)	0.33 (0.15, 0.60)	**0.018 (0.006, 0.029)**	**0.004**	25424.4 (10809.1, 52953.1)	28.4 (12.3, 58.8)	0.33 (-0.05, 1.53)	0.33 (0.14, 0.61)	0.172 (-0.055, 0.399)	0.131
Turkmenistan	615.2 (334.9, 1050.0)	17.6 (9.4, 30.3)	0.47 (0.13, 1.37)	0.24 (0.13, 0.37)	0.060 (-0.025, 0.146)	0.160	15263.0 (8804.8, 25200.1)	376.5 (212.7, 631.5)	0.49 (0.12, 1.25)	0.23 (0.12, 0.38)	1.489 (-0.420, 3.398)	0.122

Tuvalu	0.3 (0.2, 0.4)	2.7 (1.7, 3.9)	-0.07 (-0.32, 0.25)	-0.52 (-0.63, -0.43)	**0.066 (0.024, 0.107)**	**0.003**	6.5 (4.0, 9.4)	61.7 (38.2, 89.2)	-0.10 (-0.36, 0.23)	-0.52 (-0.64, -0.44)	**1.417 (0.478, 2.356)**	**0.004**
Uganda	-0.3 (-46.6, 32.1)	-0.0 (-0.4, 0.2)	-0.81 (-26.81, 16.55)	-1.10 (-4.81, 3.73)	**0.004 (0.001, 0.008)**	**0.017**	71.3 (-1183.8, 887.4)	-0.0 (-7.7, 5.3)	-0.98 (-22.69, 28.62)	-1.01 (-4.04, 3.91)	**0.096 (0.019, 0.173)**	**0.016**
Ukraine	1069.2 (126.3, 3647.0)	1.3 (0.2, 4.6)	2.42 (0.75, 29.31)	-0.06 (-0.29, 0.43)	**0.029 (0.010, 0.049)**	**0.005**	18296.6 (2394.6, 60142.6)	23.6 (3.2, 76.1)	2.47 (0.91, 16.33)	-0.05 (-0.31, 0.45)	**0.518 (0.155, 0.881)**	**0.007**
United Arab Emirates	878.1 (555.4, 1229.6)	52.8 (33.9, 73.3)	0.12 (-0.05, 0.29)	-0.30 (-0.42, -0.17)	**1.378 (0.967, 1.789)**	**<0.001**	26852.9 (16878.2, 37955.8)	905.3 (579.1, 1254.2)	-0.06 (-0.22, 0.10)	-0.29 (-0.42, -0.16)	**16.451 (9.759, 23.143)**	**<0.001**
United Kingdom	18.3 (-0.2, 91.8)	0.0 (-0.0, 0.1)	-0.86 (-1.02, -0.70)	-0.45 (-1.05, -0.30)	**-0.001 (-0.002, 0.000)**	**0.026**	286.7 (-2.3, 1428.8)	0.2 (-0.0, 1.1)	-0.87 (-1.01, -0.71)	-0.44 (-1.03, -0.30)	**-0.022 (-0.040, -0.004)**	**0.021**
United Republic of Tanzania	44.2 (-27.2, 117.5)	0.2 (-0.1, 0.5)	-1.78 (-33.85, 13.64)	-0.12 (-2.78, 1.55)	**0.016 (0.014, 0.018)**	**<0.001**	1236.0 (-518.1, 3069.2)	4.2 (-2.5, 11.1)	-1.88 (-34.25, 30.57)	-0.13 (-2.74, 1.38)	**0.325 (0.284, 0.366)**	**<0.001**
United States of America	7196.9 (2193.8, 16198.1)	1.2 (0.4, 2.6)	-0.19 (-0.45, 0.63)	-0.12 (-0.18, -0.09)	-0.003 (-0.013, 0.006)	0.455	128610.2 (40437.2, 282975.4)	23.1 (7.3, 50.0)	-0.19 (-0.43, 0.51)	-0.14 (-0.20, -0.10)	-0.054 (-0.237, 0.130)	0.556
United States Virgin Islands	0.6 (0.4, 0.9)	0.4 (0.2, 0.5)	0.67 (-1.55, 0.13)	-0.39 (-0.46, -0.34)	**0.010 (0.001, 0.019)**	**0.032**	12.4 (8.5, 16.7)	7.3 (4.9, 10.0)	0.67 (-1.57, 0.18)	-0.39 (-0.46, -0.34)	**0.201 (0.023, 0.379)**	**0.028**
Uruguay	6.6 (0.7, 19.2)	0.1 (0.0, 0.3)	-0.78 (-0.94, -0.69)	-0.41 (-0.71, -0.30)	**-0.003 (-0.007, 0.000)**	**0.033**	114.1 (13.3, 323.8)	2.1 (0.3, 5.9)	-0.78 (-0.93, -0.70)	-0.41 (-0.67, -0.31)	**-0.074 (-0.136, -0.012)**	**0.020**
Uzbekistan	1710.9 (705.8, 3688.3)	8.2 (3.3, 18.3)	0.79 (0.47, 1.93)	0.15 (0.03, 0.27)	**0.112 (0.075, 0.148)**	**<0.001**	39353.6 (17041.8, 81040.0)	158.8 (66.1, 337.4)	0.73 (0.40, 1.72)	0.15 (0.02, 0.27)	**1.934 (1.200, 2.669)**	**<0.001**
Vanuatu	0.6 (-0.4, 0.9)	0.4 (-0.3, 0.6)	-3.80 (-14.86, 21.63)	0.08 (-0.14, 0.36)	**0.021 (0.013, 0.030)**	**<0.001**	17.6 (-11.1, 25.6)	9.2 (-6.1, 13.3)	-4.37 (-2.84, 20.19)	0.08 (-0.13, 0.34)	**0.487 (0.293, 0.680)**	**<0.001**
Venezuela (Bolivarian Republic of)	361.7 (193.9, 469.3)	1.3 (0.7, 1.6)	1.00 (-27.25, 13.68)	-0.50 (-0.63, -0.31)	0.100 (-0.015, 0.215)	0.086	7889.1 (4314.8, 10355.4)	26.4 (14.4, 34.6)	0.95 (-27.23, 19.24)	-0.49 (-0.63, -0.31)	2.052 (-0.361, 4.465)	0.093
Viet Nam	5976.3 (2306.1, 9920.2)	7.0 (2.7, 11.6)	0.73 (0.23, 3.49)	-0.19 (-0.29, -0.15)	**0.139 (0.090, 0.188)**	**<0.001**	127102.5 (49535.4, 211125.2)	133.8 (51.9, 221.7)	0.63 (0.13, 2.99)	-0.20 (-0.29, -0.15)	**2.535 (1.597, 3.473)**	**<0.001**
Yemen	928.0 (582.4, 1384.8)	8.2 (5.2, 12.1)	-0.10 (-0.34, 0.26)	-0.09 (-0.14, -0.01)	**0.046 (0.016, 0.076)**	**0.004**	23336.9 (14339.5, 35185.8)	162.1 (101.3, 242.2)	-0.17 (-0.41, 0.16)	-0.09 (-0.15, -0.02)	0.489 (-0.130, 1.109)	0.117
Zambia	122.0 (-12.1, 281.0)	2.1 (-0.3, 5.0)	2.09 (-13.62, 13.48)	0.13 (-0.25, 1.07)	**0.022 (0.003, 0.041)**	**0.022**	3510.8 (-127.7, 7607.8)	44.4 (-4.6, 102.7)	1.95 (-13.74, 17.56)	0.14 (-0.10, 0.87)	**0.443 (0.034, 0.852)**	**0.035**
Zimbabwe	104.8 (-6.2, 242.3)	1.8 (-0.2, 4.3)	2.11 (-9.71, 11.74)	-0.35 (-0.48, -0.17)	**0.044 (0.024, 0.064)**	**<0.001**	2880.8 (-131.0, 6367.5)	39.6 (-2.5, 91.3)	2.38 (-9.26, 12.60)	-0.33 (-0.45, -0.09)	**0.964 (0.551, 1.377)**	**<0.001**

ASMR, age-standardized mortality rates; ASRDALYs, age-standardized rates of DALYs; DALYs, disability-adjusted life-years.

Table S10. Deaths and DALYs attributable to non-communicable diseases associated with low temperature in 204 countries and territories in 2021 and relative change from 1990 to 2021 and 2019 to 2021.

**location**	**Number of cases**	**ASMR**	**Relative change in ASMR 1990-2021**	**Relative change in ASMR 2019-2021**	**Trends of age-standardized rate from 1990 to 2021 (β)**	***P* value**	**Number of cases**	**ASRDALYs**	**Relative change in ASRDALYs 1990-2021**	**Relative change in ASRDALYs 2019-2021**	**Trends of age-standardized rate from 1990 to 2021 (β)**	***P* value**
Afghanistan	4292.6 (3458.8, 5186.5)	52.1 (42.4, 61.7)	-0.29 (-0.43, -0.12)	-0.08 (-0.12, -0.03)	**-0.850 (-0.960, -0.740)**	**<0.001**	122569.4 (96929.3, 151262.9)	1107.0 (892.2, 1335.0)	-0.32 (-0.46, -0.13)	-0.07 (-0.12, -0.02)	**-21.235 (-23.651, -18.819)**	**<0.001**
Albania	1261.3 (1076.9, 1465.3)	30.9 (26.6, 36.0)	-0.23 (-0.33, -0.13)	0.04 (-0.07, 0.17)	**-0.306 (-0.400, -0.213)**	**<0.001**	19794.7 (16666.7, 22938.4)	473.6 (399.7, 548.6)	-0.31 (-0.41, -0.21)	0.02 (-0.09, 0.16)	**-7.366 (-8.933, -5.799)**	**<0.001**
Algeria	8914.5 (7604.9, 10473.4)	35.6 (30.7, 41.3)	-0.33 (-0.42, -0.21)	-0.11 (-0.16, -0.07)	**-0.520 (-0.616, -0.424)**	**<0.001**	175579.4 (148191.6, 209445.4)	564.7 (483.6, 666.7)	-0.38 (-0.48, -0.26)	-0.10 (-0.16, -0.05)	**-12.159 (-13.726, -10.591)**	**<0.001**
American Samoa	0.4 (0.2, 0.6)	0.9 (0.6, 1.4)	0.09 (-0.23, 2.99)	1.08 (0.88, 1.36)	**-0.013 (-0.027, 0.000)**	**0.048**	10.1 (6.0, 14.9)	21.1 (12.8, 30.9)	0.12 (-0.22, 3.09)	1.08 (0.87, 1.37)	-0.275 (-0.567, 0.016)	0.063
Andorra	16.8 (12.1, 21.3)	9.6 (6.9, 12.2)	-0.42 (-0.59, -0.21)	-0.11 (-0.23, -0.04)	**-0.256 (-0.295, -0.217)**	**<0.001**	245.0 (177.3, 315.2)	151.5 (109.7, 196.0)	-0.46 (-0.63, -0.24)	-0.12 (-0.26, -0.03)	**-4.773 (-5.455, -4.092)**	**<0.001**
Angola	848.3 (659.5, 1068.2)	9.7 (7.7, 12.0)	-0.28 (-0.47, -0.02)	0.15 (0.06, 0.27)	**-0.162 (-0.203, -0.121)**	**<0.001**	22786.5 (17445.4, 28841.2)	192.3 (150.2, 241.9)	-0.33 (-0.52, -0.04)	0.15 (0.06, 0.27)	**-3.919 (-4.778, -3.061)**	**<0.001**
Antigua and Barbuda	0.4 (0.2, 0.5)	0.4 (0.3, 0.6)	-0.66 (-0.73, -0.61)	-0.30 (-0.39, -0.20)	**-0.021 (-0.031, -0.010)**	**<0.001**	7.9 (5.0, 10.8)	7.8 (4.9, 10.6)	-0.69 (-0.75, -0.63)	-0.29 (-0.39, -0.19)	**-0.418 (-0.610, -0.226)**	**<0.001**
Argentina	8449.6 (7663.4, 8959.1)	14.6 (13.2, 15.5)	-0.53 (-0.55, -0.51)	-0.14 (-0.16, -0.12)	**-0.489 (-0.538, -0.440)**	**<0.001**	152978.3 (142238.5, 160691.5)	273.6 (255.1, 287.2)	-0.56 (-0.58, -0.54)	-0.13 (-0.15, -0.11)	**-10.560 (-11.506, -9.614)**	**<0.001**
Armenia	1017.0 (905.8, 1137.5)	23.7 (21.0, 26.5)	-0.42 (-0.47, -0.37)	-0.07 (-0.14, 0.01)	**-0.895 (-1.033, -0.757)**	**<0.001**	18033.2 (16129.3, 20207.6)	423.4 (378.6, 474.2)	-0.44 (-0.49, -0.39)	-0.07 (-0.14, 0.02)	**-17.029 (-19.457, -14.601)**	**<0.001**
Australia	4143.2 (3492.4, 4562.5)	7.9 (6.8, 8.7)	-0.63 (-0.66, -0.62)	0.05 (0.03, 0.06)	**-0.473 (-0.519, -0.428)**	**<0.001**	60325.2 (52784.2, 65589.6)	128.6 (114.3, 139.2)	-0.66 (-0.68, -0.64)	0.04 (0.03, 0.05)	**-8.439 (-9.320, -7.558)**	**<0.001**
Austria	2772.6 (2333.9, 3031.3)	12.3 (10.6, 13.4)	-0.56 (-0.59, -0.54)	0.03 (0.00, 0.05)	**-0.568 (-0.625, -0.512)**	**<0.001**	38133.1 (33607.7, 41196.7)	191.6 (172.7, 205.3)	-0.59 (-0.62, -0.58)	0.02 (-0.01, 0.04)	**-10.170 (-11.212, -9.128)**	**<0.001**
Azerbaijan	3250.4 (2895.5, 3664.1)	40.5 (36.1, 45.5)	-0.20 (-0.28, -0.12)	-0.07 (-0.15, 0.01)	**-0.369 (-0.511, -0.226)**	**<0.001**	70849.3 (62510.7, 80097.6)	753.8 (670.6, 848.8)	-0.26 (-0.34, -0.18)	-0.06 (-0.16, 0.02)	**-12.742 (-15.686, -9.798)**	**<0.001**
Bahamas	9.7 (7.7, 12.2)	2.6 (2.1, 3.3)	-0.10 (-0.31, 0.25)	2.65 (0.84, 53.88)	**-0.059 (-0.107, -0.012)**	**0.016**	232.7 (180.8, 293.2)	56.8 (44.4, 71.2)	-0.14 (-0.35, 0.21)	2.67 (0.84, 53.97)	**-1.319 (-2.325, -0.313)**	**0.012**
Bahrain	101.3 (75.9, 131.2)	20.4 (15.4, 25.9)	-0.57 (-0.64, -0.49)	-0.13 (-0.18, -0.08)	**-1.107 (-1.336, -0.878)**	**<0.001**	2588.6 (1943.7, 3365.5)	339.8 (255.7, 436.4)	-0.61 (-0.68, -0.54)	-0.14 (-0.19, -0.08)	**-22.665 (-26.443, -18.888)**	**<0.001**

Bangladesh	13032.9 (9502.8, 17428.2)	10.8 (8.0, 14.4)	-0.11 (-0.32, 0.24)	-0.05 (-0.10, 0.00)	-0.025 (-0.090, 0.039)	0.431	288370.5 (210386.9, 384089.4)	212.8 (156.4, 283.1)	-0.23 (-0.42, 0.09)	-0.05 (-0.11, 0.01)	**-1.353 (-2.648, -0.057)**	**0.041**
Barbados	0.5 (0.2, 0.8)	0.1 (0.0, 0.1)	-0.83 (-0.90, -0.78)	-0.50 (-0.60, -0.44)	**-0.008 (-0.012, -0.004)**	**<0.001**	8.2 (2.9, 13.9)	1.7 (0.6, 2.8)	-0.83 (-0.89, -0.77)	-0.51 (-0.61, -0.44)	**-0.151 (-0.221, -0.080)**	**<0.001**
Belarus	5498.7 (4624.2, 6521.1)	33.8 (28.4, 40.1)	-0.18 (-0.31, -0.02)	0.12 (-0.05, 0.30)	**-0.643 (-0.800, -0.487)**	**<0.001**	100895.8 (84508.8, 120197.7)	641.1 (537.1, 761.6)	-0.18 (-0.33, -0.02)	0.12 (-0.06, 0.32)	**-13.516 (-17.262, -9.770)**	**<0.001**
Belgium	2675.1 (2217.8, 2993.3)	9.3 (7.9, 10.3)	-0.59 (-0.62, -0.57)	-0.02 (-0.03, 0.00)	**-0.492 (-0.532, -0.452)**	**<0.001**	38703.6 (33489.3, 42507.4)	155.5 (137.9, 170.2)	-0.60 (-0.62, -0.58)	-0.00 (-0.02, 0.02)	**-8.634 (-9.357, -7.911)**	**<0.001**
Belize	3.6 (2.9, 4.6)	1.3 (1.0, 1.7)	-0.57 (-0.64, -0.49)	-0.08 (-0.16, 0.05)	**-0.071 (-0.105, -0.038)**	**<0.001**	86.8 (68.4, 112.1)	27.9 (22.0, 35.8)	-0.58 (-0.65, -0.50)	-0.08 (-0.17, 0.05)	**-1.513 (-2.206, -0.819)**	**<0.001**
Benin	24.4 (9.4, 38.7)	0.6 (0.2, 0.9)	-0.63 (-0.75, -0.13)	-0.30 (-0.37, 0.09)	**-0.037 (-0.051, -0.022)**	**<0.001**	622.4 (234.6, 996.4)	11.2 (4.3, 17.7)	-0.64 (-0.77, -0.16)	-0.31 (-0.37, 0.08)	**-0.798 (-1.096, -0.500)**	**<0.001**
Bermuda	6.9 (5.8, 8.4)	4.6 (3.9, 5.6)	-0.65 (-0.70, -0.58)	0.04 (-0.01, 0.10)	**-0.297 (-0.332, -0.262)**	**<0.001**	113.2 (95.6, 137.8)	83.3 (70.8, 101.5)	-0.67 (-0.72, -0.60)	0.04 (-0.01, 0.11)	**-5.594 (-6.300, -4.888)**	**<0.001**
Bhutan	162.0 (133.5, 192.2)	29.6 (24.6, 34.7)	-0.20 (-0.38, 0.06)	-0.02 (-0.07, 0.02)	**-0.279 (-0.334, -0.224)**	**<0.001**	3220.1 (2555.9, 3933.4)	543.3 (435.5, 659.6)	-0.29 (-0.45, -0.03)	-0.03 (-0.07, 0.02)	**-8.062 (-9.222, -6.903)**	**<0.001**
Bolivia (Plurinational State of)	1498.4 (1219.0, 1931.3)	19.2 (15.7, 24.6)	-0.33 (-0.44, -0.15)	0.02 (-0.05, 0.08)	**-0.309 (-0.351, -0.267)**	**<0.001**	33392.3 (26443.0, 43514.3)	373.2 (300.4, 483.3)	-0.39 (-0.51, -0.20)	0.03 (-0.04, 0.10)	**-7.939 (-8.897, -6.982)**	**<0.001**
Bosnia and Herzegovina	1777.6 (1443.7, 2064.8)	27.8 (22.5, 32.4)	-0.25 (-0.38, -0.15)	0.03 (-0.12, 0.17)	**-0.547 (-0.657, -0.436)**	**<0.001**	29941.9 (24095.9, 34794.7)	480.0 (385.9, 558.7)	-0.32 (-0.44, -0.21)	0.02 (-0.15, 0.18)	**-11.932 (-13.949, -9.916)**	**<0.001**
Botswana	195.4 (158.8, 236.7)	17.1 (14.3, 20.5)	-0.23 (-0.38, 0.07)	0.27 (0.18, 0.38)	**-0.250 (-0.347, -0.153)**	**<0.001**	4619.0 (3650.9, 5594.5)	329.6 (267.0, 398.3)	-0.25 (-0.43, 0.05)	0.28 (0.18, 0.40)	**-5.647 (-7.634, -3.660)**	**<0.001**
Brazil	15440.6 (13428.5, 17252.3)	6.3 (5.5, 7.1)	-0.52 (-0.55, -0.50)	0.30 (0.25, 0.33)	**-0.262 (-0.294, -0.230)**	**<0.001**	326574.4 (292773.3, 360621.3)	130.7 (116.7, 144.3)	-0.53 (-0.55, -0.51)	0.32 (0.27, 0.36)	**-5.736 (-6.430, -5.042)**	**<0.001**
Brunei Darussalam	0.8 (0.6, 1.1)	0.3 (0.2, 0.4)	-0.66 (-0.74, -0.56)	0.68 (0.39, 1.04)	**-0.026 (-0.034, -0.017)**	**<0.001**	21.8 (16.2, 28.6)	6.3 (4.7, 8.3)	-0.68 (-0.75, -0.57)	0.77 (0.45, 1.16)	**-0.534 (-0.699, -0.368)**	**<0.001**
Bulgaria	6881.4 (6055.9, 7870.1)	48.1 (42.4, 54.8)	-0.26 (-0.34, -0.18)	0.12 (0.01, 0.23)	**-0.899 (-1.063, -0.736)**	**<0.001**	117058.8 (101751.8, 135980.8)	851.0 (738.6, 990.4)	-0.24 (-0.33, -0.14)	0.13 (-0.00, 0.26)	**-16.175 (-19.132, -13.218)**	**<0.001**
Burkina Faso	131.2 (84.6, 209.6)	1.7 (1.1, 2.6)	-0.62 (-0.70, -0.38)	-0.45 (-0.49, -0.40)	**-0.091 (-0.147, -0.034)**	**0.003**	3349.5 (2045.1, 5318.8)	33.7 (21.7, 53.8)	-0.63 (-0.72, -0.41)	-0.46 (-0.50, -0.41)	**-2.052 (-3.219, -0.885)**	**0.001**
Burundi	505.5 (395.1, 652.4)	13.0 (10.1, 16.9)	-0.46 (-0.58, -0.29)	0.02 (-0.03, 0.10)	**-0.494 (-0.561, -0.426)**	**<0.001**	13595.9 (10589.1, 17367.2)	264.4 (206.4, 341.8)	-0.50 (-0.61, -0.31)	0.02 (-0.04, 0.09)	**-11.403 (-12.909, -9.897)**	**<0.001**

Cabo Verde	43.9 (35.8, 53.5)	10.6 (8.6, 12.8)	0.30 (0.04, 0.66)	-0.08 (-0.13, -0.04)	0.027 (-0.042, 0.096)	0.433	861.3 (682.0, 1058.3)	195.5 (155.6, 237.7)	0.18 (-0.08, 0.53)	-0.08 (-0.13, -0.03)	-0.119 (-1.416, 1.178)	0.852
Cambodia	339.4 (195.9, 485.7)	3.4 (2.0, 4.9)	0.46 (0.00, 1.10)	0.83 (0.71, 1.27)	-0.045 (-0.091, 0.001)	0.055	8205.0 (4791.7, 11920.6)	68.1 (39.5, 97.8)	0.32 (-0.11, 0.90)	0.83 (0.70, 1.26)	**-1.152 (-2.136, -0.167)**	**0.023**
Cameroon	543.6 (406.6, 712.5)	5.3 (4.1, 6.9)	-0.28 (-0.42, -0.03)	-0.16 (-0.22, -0.09)	**-0.095 (-0.149, -0.042)**	**<0.001**	14641.8 (10653.3, 19377.9)	108.1 (80.6, 142.0)	-0.29 (-0.45, -0.03)	-0.17 (-0.23, -0.08)	**-2.094 (-3.251, -0.936)**	**<0.001**
Canada	7156.1 (6120.6, 7744.3)	8.8 (7.6, 9.5)	-0.55 (-0.57, -0.52)	-0.14 (-0.16, -0.13)	**-0.386 (-0.419, -0.353)**	**<0.001**	108992.8 (97168.1, 116051.0)	149.5 (135.5, 158.7)	-0.56 (-0.59, -0.54)	-0.14 (-0.16, -0.13)	**-6.842 (-7.462, -6.222)**	**<0.001**
Central African Republic	26.9 (12.7, 44.5)	1.6 (0.8, 2.6)	-0.44 (-0.63, -0.04)	-0.21 (-0.32, 0.00)	**-0.077 (-0.102, -0.052)**	**<0.001**	804.9 (375.1, 1346.6)	34.3 (16.5, 56.0)	-0.45 (-0.64, -0.06)	-0.21 (-0.32, 0.01)	**-1.738 (-2.294, -1.182)**	**<0.001**
Chad	206.9 (131.5, 303.2)	4.2 (2.7, 6.0)	-0.11 (-0.28, 0.12)	-0.25 (-0.30, -0.19)	**-0.112 (-0.188, -0.036)**	**0.005**	5747.1 (3584.1, 8521.1)	87.6 (55.8, 128.5)	-0.11 (-0.31, 0.14)	-0.25 (-0.31, -0.19)	**-2.411 (-4.020, -0.801)**	**0.005**
Chile	3395.4 (2983.9, 3773.6)	12.9 (11.4, 14.4)	-0.52 (-0.55, -0.49)	-0.02 (-0.04, -0.00)	**-0.356 (-0.403, -0.309)**	**<0.001**	57616.0 (52832.3, 63367.8)	225.6 (207.2, 248.1)	-0.55 (-0.57, -0.52)	-0.02 (-0.03, 0.00)	**-7.283 (-8.083, -6.484)**	**<0.001**
China	549968.4 (463887.6, 649295.2)	30.3 (25.6, 35.7)	-0.50 (-0.58, -0.41)	-0.02 (-0.18, 0.17)	**-1.101 (-1.173, -1.029)**	**<0.001**	9641543.0 (7981159.0, 11429410.1)	493.4 (412.7, 584.5)	-0.54 (-0.62, -0.44)	-0.02 (-0.20, 0.19)	**-20.427 (-21.624, -19.229)**	**<0.001**
Colombia	4982.2 (4093.1, 5976.5)	8.8 (7.3, 10.5)	-0.49 (-0.56, -0.38)	0.16 (0.00, 0.35)	**-0.357 (-0.399, -0.316)**	**<0.001**	88508.1 (73584.6, 106738.3)	159.6 (132.6, 192.7)	-0.53 (-0.61, -0.43)	0.17 (-0.00, 0.38)	**-7.593 (-8.450, -6.736)**	**<0.001**
Comoros	11.3 (7.2, 16.9)	2.8 (1.8, 4.1)	-0.27 (-0.51, 0.13)	0.39 (0.24, 0.94)	**-0.062 (-0.078, -0.047)**	**<0.001**	271.1 (169.7, 400.2)	55.3 (34.9, 82.0)	-0.31 (-0.55, 0.08)	0.40 (0.24, 0.94)	**-1.444 (-1.776, -1.111)**	**<0.001**
Congo	48.5 (32.2, 71.3)	2.4 (1.6, 3.5)	-0.64 (-0.74, -0.47)	0.00 (-0.08, 0.34)	**-0.145 (-0.175, -0.115)**	**<0.001**	1285.9 (850.2, 1907.2)	47.7 (31.8, 70.2)	-0.66 (-0.76, -0.50)	0.00 (-0.08, 0.33)	**-3.206 (-3.844, -2.569)**	**<0.001**
Cook Islands	0.0 (0.0, 0.0)	0.1 (0.0, 0.1)	-0.14 (-0.41, 0.34)	5.93 (2.26, 31.49)	-0.003 (-0.008, 0.002)	0.189	0.3 (0.1, 0.5)	1.2 (0.6, 2.0)	-0.15 (-0.44, 0.36)	5.93 (2.22, 31.61)	-0.068 (-0.171, 0.035)	0.188
Costa Rica	308.5 (258.0, 358.4)	5.5 (4.6, 6.4)	-0.30 (-0.39, -0.22)	0.18 (0.07, 0.28)	**-0.161 (-0.200, -0.122)**	**<0.001**	5754.8 (4965.8, 6739.3)	104.3 (90.2, 122.1)	-0.29 (-0.37, -0.20)	0.22 (0.10, 0.34)	**-2.949 (-3.663, -2.234)**	**<0.001**
Croatia	2297.1 (2000.4, 2602.6)	23.1 (20.1, 26.2)	-0.50 (-0.54, -0.46)	0.09 (0.00, 0.16)	**-0.937 (-1.025, -0.848)**	**<0.001**	34012.6 (29671.0, 38663.9)	365.4 (319.2, 415.9)	-0.53 (-0.58, -0.49)	0.08 (-0.02, 0.16)	**-16.643 (-18.102, -15.184)**	**<0.001**
Cte d'Ivoire	64.1 (27.6, 103.8)	0.7 (0.3, 1.2)	-0.63 (-0.76, 0.32)	-0.20 (-0.27, 0.12)	**-0.041 (-0.053, -0.029)**	**<0.001**	1772.4 (766.6, 2953.1)	14.6 (6.3, 23.7)	-0.64 (-0.77, 0.27)	-0.20 (-0.28, 0.11)	**-0.888 (-1.146, -0.631)**	**<0.001**
Cuba	453.9 (369.5, 541.0)	2.2 (1.8, 2.6)	-0.40 (-0.48, -0.32)	0.13 (0.00, 0.26)	**-0.078 (-0.115, -0.042)**	**<0.001**	8196.4 (6705.4, 9736.4)	42.1 (34.4, 49.9)	-0.39 (-0.47, -0.30)	0.11 (-0.02, 0.25)	**-1.434 (-2.122, -0.747)**	**<0.001**

Cyprus	250.7 (212.6, 288.5)	15.1 (13.0, 17.0)	-0.70 (-0.73, -0.67)	-0.14 (-0.18, -0.09)	**-1.303 (-1.469, -1.137)**	**<0.001**	3895.3 (3331.7, 4480.9)	212.7 (182.1, 242.9)	-0.71 (-0.74, -0.67)	-0.15 (-0.19, -0.09)	**-18.646 (-20.647, -16.644)**	**<0.001**
Czechia	4177.1 (3651.3, 4627.7)	17.9 (15.7, 19.8)	-0.59 (-0.63, -0.55)	0.09 (0.01, 0.17)	**-0.898 (-0.970, -0.826)**	**<0.001**	65206.8 (56903.8, 72520.3)	295.8 (259.1, 329.6)	-0.63 (-0.67, -0.59)	0.09 (-0.00, 0.19)	**-16.960 (-18.525, -15.396)**	**<0.001**
Democratic People's Republic of Korea	12210.5 (10064.6, 14673.0)	41.6 (34.4, 49.7)	-0.19 (-0.34, 0.01)	-0.04 (-0.09, 0.01)	**-0.327 (-0.423, -0.231)**	**<0.001**	256620.6 (206757.7, 309799.2)	808.3 (654.0, 976.4)	-0.15 (-0.34, 0.09)	-0.03 (-0.09, 0.02)	**-5.163 (-7.002, -3.323)**	**<0.001**
Democratic Republic of the Congo	1885.2 (1428.9, 2354.8)	6.8 (5.3, 8.6)	-0.24 (-0.43, 0.06)	0.02 (-0.05, 0.14)	**-0.111 (-0.138, -0.084)**	**<0.001**	48908.3 (36505.4, 61122.9)	135.5 (102.9, 169.2)	-0.27 (-0.46, 0.02)	0.02 (-0.05, 0.14)	**-2.475 (-3.031, -1.918)**	**<0.001**
Denmark	1691.0 (1471.2, 1886.7)	12.4 (10.9, 13.8)	-0.50 (-0.54, -0.46)	0.02 (-0.01, 0.04)	**-0.561 (-0.616, -0.506)**	**<0.001**	24687.9 (21954.4, 27400.3)	196.9 (177.1, 216.9)	-0.56 (-0.60, -0.53)	0.00 (-0.02, 0.03)	**-11.027 (-12.022, -10.032)**	**<0.001**
Djibouti	53.5 (34.9, 80.1)	11.2 (7.5, 16.2)	0.08 (-0.20, 0.49)	0.28 (0.17, 0.44)	-0.031 (-0.106, 0.045)	0.409	1479.0 (939.6, 2244.9)	226.5 (147.6, 336.9)	0.05 (-0.25, 0.49)	0.27 (0.17, 0.43)	-0.880 (-2.482, 0.723)	0.271
Dominica	0.5 (0.3, 0.7)	0.7 (0.4, 0.9)	-0.66 (-0.72, -0.59)	-0.17 (-0.22, -0.12)	**-0.036 (-0.049, -0.022)**	**<0.001**	10.8 (7.2, 15.0)	13.5 (9.0, 18.6)	-0.64 (-0.70, -0.56)	-0.17 (-0.22, -0.12)	**-0.631 (-0.886, -0.375)**	**<0.001**
Dominican Republic	353.6 (283.2, 439.7)	3.6 (2.9, 4.5)	-0.23 (-0.42, -0.00)	0.17 (-0.04, 0.44)	-0.018 (-0.042, 0.007)	0.145	7827.7 (6262.2, 9610.7)	77.2 (61.7, 95.1)	-0.18 (-0.38, 0.06)	0.14 (-0.06, 0.40)	-0.277 (-0.733, 0.180)	0.225
Ecuador	1356.1 (1073.1, 1683.5)	9.1 (7.3, 11.2)	-0.27 (-0.40, -0.11)	-0.00 (-0.15, 0.15)	**-0.131 (-0.179, -0.084)**	**<0.001**	26864.5 (20778.0, 34107.8)	167.6 (130.5, 211.8)	-0.31 (-0.46, -0.13)	0.01 (-0.16, 0.21)	**-3.198 (-4.189, -2.208)**	**<0.001**
Egypt	15406.1 (12661.2, 18929.4)	34.0 (28.2, 41.1)	-0.28 (-0.38, -0.16)	-0.12 (-0.24, -0.00)	**-0.345 (-0.469, -0.221)**	**<0.001**	389964.2 (316705.3, 482048.3)	653.3 (539.3, 799.9)	-0.29 (-0.40, -0.17)	-0.12 (-0.25, 0.03)	**-8.166 (-10.520, -5.811)**	**<0.001**
El Salvador	29.8 (22.4, 43.0)	0.4 (0.3, 0.6)	-0.47 (-0.66, -0.18)	-0.01 (-0.20, 0.25)	-0.016 (-0.033, 0.001)	0.064	598.0 (456.4, 850.6)	9.5 (7.2, 13.5)	-0.48 (-0.67, -0.18)	-0.01 (-0.21, 0.26)	-0.341 (-0.697, 0.015)	0.060
Equatorial Guinea	12.4 (8.3, 17.3)	3.2 (2.2, 4.3)	-0.61 (-0.74, -0.44)	0.05 (-0.05, 0.15)	**-0.151 (-0.179, -0.123)**	**<0.001**	321.8 (206.4, 463.1)	61.3 (40.6, 85.2)	-0.65 (-0.77, -0.48)	0.05 (-0.04, 0.16)	**-3.517 (-4.132, -2.902)**	**<0.001**
Eritrea	253.3 (196.1, 312.8)	12.1 (9.8, 14.5)	-0.21 (-0.35, -0.02)	0.04 (-0.00, 0.09)	**-0.203 (-0.265, -0.141)**	**<0.001**	7193.1 (5472.5, 9111.8)	248.5 (193.3, 304.1)	-0.27 (-0.41, -0.08)	0.04 (-0.00, 0.10)	**-5.203 (-6.562, -3.844)**	**<0.001**
Estonia	559.6 (475.7, 642.6)	17.3 (14.9, 19.9)	-0.58 (-0.64, -0.52)	0.00 (-0.09, 0.09)	**-1.035 (-1.117, -0.952)**	**<0.001**	8249.3 (7116.2, 9426.9)	291.9 (252.9, 332.6)	-0.62 (-0.67, -0.56)	-0.01 (-0.11, 0.08)	**-20.890 (-22.705, -19.074)**	**<0.001**
Eswatini	163.5 (126.5, 211.6)	35.6 (28.3, 44.6)	-0.03 (-0.24, 0.30)	0.05 (-0.01, 0.12)	0.068 (-0.169, 0.304)	0.563	4355.1 (3259.0, 5783.8)	758.6 (587.1, 980.8)	0.02 (-0.22, 0.39)	0.09 (0.03, 0.17)	2.385 (-3.264, 8.034)	0.395

Ethiopia	5687.0<br>(4692.7, 6764.7)	15.5<br>(12.7, 18.3)	-0.50<br>(-0.59, -0.41)	0.09<br>(-0.04, 0.24)	**-0.610<br>(-0.681, -0.539)**	**<0.001**	139111.8<br>(115156.4, 166226.9)	304.7<br>(251.2, 363.6)	-0.56<br>(-0.64, -0.47)	0.09<br>(-0.06, 0.27)	**-14.877<br>(-16.508, -13.246)**	**<0.001**
Fiji	43.7<br>(32.8, 62.8)	6.9<br>(5.3, 9.5)	-0.43<br>(-0.56, -0.22)	-0.16<br>(-0.23, -0.08)	**-0.251<br>(-0.347, -0.155)**	**<0.001**	1159.7<br>(856.0, 1703.5)	150.0<br>(112.7, 212.9)	-0.46<br>(-0.59, -0.22)	-0.16<br>(-0.24, -0.07)	**-5.772<br>(-7.878, -3.666)**	**<0.001**
Finland	1727.0<br>(1419.9, 1937.7)	11.0<br>(9.2, 12.3)	-0.57<br>(-0.60, -0.54)	-0.05<br>(-0.08, -0.01)	**-0.487<br>(-0.529, -0.446)**	**<0.001**	24162.9<br>(20695.8, 26911.2)	178.1<br>(157.5, 197.0)	-0.63<br>(-0.65, -0.60)	-0.05<br>(-0.08, -0.01)	**-9.948<br>(-10.810, -9.086)**	**<0.001**
France	13705.5<br>(11276.5, 15229.5)	7.1<br>(6.0, 7.8)	-0.56<br>(-0.59, -0.54)	-0.01<br>(-0.02, 0.01)	**-0.350<br>(-0.378, -0.322)**	**<0.001**	178121.2<br>(152289.0, 196338.1)	112.6<br>(99.5, 122.6)	-0.57<br>(-0.59, -0.55)	-0.01<br>(-0.02, 0.01)	**-5.774<br>(-6.237, -5.311)**	**<0.001**
Gabon	21.3<br>(14.2, 29.2)	2.6<br>(1.8, 3.6)	-0.62<br>(-0.71, -0.49)	0.09<br>(-0.01, 0.31)	**-0.155<br>(-0.183, -0.127)**	**<0.001**	512.8<br>(336.2, 715.5)	50.7<br>(33.5, 69.3)	-0.64<br>(-0.72, -0.51)	0.08<br>(-0.02, 0.30)	**-3.212<br>(-3.767, -2.657)**	**<0.001**
Gambia	19.3<br>(12.5, 26.1)	2.3<br>(1.5, 3.1)	-0.37<br>(-0.55, -0.13)	-0.46<br>(-0.51, -0.39)	0.007<br>(-0.033, 0.047)	0.717	481.2<br>(312.8, 650.6)	46.5<br>(30.1, 63.1)	-0.39<br>(-0.57, -0.14)	-0.47<br>(-0.52, -0.39)	0.032<br>(-0.791, 0.855)	0.938
Georgia	1980.0<br>(1770.2, 2300.8)	30.9<br>(27.8, 35.7)	-0.36<br>(-0.42, -0.29)	0.00<br>(-0.08, 0.08)	**-0.691<br>(-0.807, -0.575)**	**<0.001**	34849.9<br>(31505.1, 39614.9)	586.3<br>(531.5, 661.2)	-0.37<br>(-0.44, -0.30)	-0.01<br>(-0.09, 0.07)	**-16.541<br>(-19.342, -13.741)**	**<0.001**
Germany	30079.7<br>(25474.7, 33321.8)	12.6<br>(11.0, 13.9)	-0.53<br>(-0.56, -0.50)	0.04<br>(0.03, 0.06)	**-0.520<br>(-0.582, -0.458)**	**<0.001**	426188.4<br>(376689.9, 467451.0)	205.8<br>(185.6, 222.9)	-0.56<br>(-0.58, -0.54)	0.04<br>(0.03, 0.05)	**-9.516<br>(-10.649, -8.382)**	**<0.001**
Ghana	83.7<br>(37.7, 129.4)	0.6<br>(0.3, 0.9)	-0.79<br>(-0.87, -0.71)	-0.13<br>(-0.21, 0.13)	**-0.091<br>(-0.111, -0.072)**	**<0.001**	2230.2<br>(988.8, 3466.5)	12.6<br>(5.6, 19.4)	-0.80<br>(-0.87, -0.72)	-0.14<br>(-0.22, 0.11)	**-1.969<br>(-2.375, -1.563)**	**<0.001**
Greece	4788.3<br>(4094.3, 5191.2)	15.1<br>(13.3, 16.3)	-0.49<br>(-0.51, -0.47)	0.05<br>(0.03, 0.06)	**-0.597<br>(-0.644, -0.549)**	**<0.001**	65994.9<br>(58403.3, 70909.7)	255.8<br>(233.7, 273.1)	-0.47<br>(-0.49, -0.45)	0.04<br>(0.02, 0.06)	**-9.256<br>(-9.986, -8.525)**	**<0.001**
Greenland	10.7<br>(8.4, 13.2)	19.5<br>(15.0, 24.6)	-0.51<br>(-0.60, -0.32)	-0.11<br>(-0.17, 0.00)	**-0.665<br>(-0.774, -0.556)**	**<0.001**	240.1<br>(189.3, 292.7)	369.6<br>(288.5, 448.8)	-0.52<br>(-0.62, -0.35)	-0.10<br>(-0.17, -0.00)	**-13.272<br>(-15.309, -11.236)**	**<0.001**
Grenada	0.1<br>(0.1, 0.2)	0.1<br>(0.1, 0.2)	-0.79<br>(-0.85, -0.74)	-0.17<br>(-0.28, -0.03)	**-0.011<br>(-0.016, -0.007)**	**<0.001**	2.8<br>(1.4, 4.5)	2.5<br>(1.3, 4.0)	-0.81<br>(-0.86, -0.76)	-0.17<br>(-0.28, -0.03)	**-0.244<br>(-0.339, -0.149)**	**<0.001**
Guam	0.0<br>(0.0, 0.1)	0.0<br>(0.0, 0.0)	-0.78<br>(-0.94, -0.28)	0.39<br>(-0.65, 6.37)	**-0.003<br>(-0.004, -0.002)**	**<0.001**	0.7<br>(0.1, 2.6)	0.4<br>(0.1, 1.3)	-0.69<br>(-0.92, 0.04)	0.46<br>(-0.64, 6.94)	**-0.052<br>(-0.074, -0.030)**	**<0.001**
Guatemala	968.6<br>(822.9, 1201.2)	9.9<br>(8.4, 12.2)	-0.36<br>(-0.45, -0.22)	-0.07<br>(-0.18, 0.04)	**-0.200<br>(-0.241, -0.159)**	**<0.001**	22320.8<br>(18782.4, 27983.1)	199.1<br>(169.0, 248.5)	-0.33<br>(-0.44, -0.19)	-0.03<br>(-0.15, 0.12)	**-3.724<br>(-4.613, -2.836)**	**<0.001**
Guinea	90.9<br>(57.6, 125.4)	1.8<br>(1.2, 2.5)	-0.35<br>(-0.54, 0.02)	-0.08<br>(-0.15, 0.10)	**-0.024<br>(-0.045, -0.004)**	**0.023**	2296.0<br>(1424.2, 3210.1)	37.9<br>(23.9, 52.4)	-0.37<br>(-0.56, -0.02)	-0.08<br>(-0.15, 0.10)	**-0.568<br>(-1.003, -0.134)**	**0.012**
Guinea-Bissau	5.0<br>(2.8, 10.7)	0.9<br>(0.5, 1.8)	-0.67<br>(-0.77, -0.39)	-0.62<br>(-0.69, -0.53)	0.000<br>(-0.043, 0.043)	0.993	147.1<br>(79.4, 314.2)	18.4<br>(10.1, 39.1)	-0.69<br>(-0.79, -0.42)	-0.62<br>(-0.69, -0.53)	-0.132<br>(-1.070, 0.805)	0.775

Guyana	4.9<br>(2.3, 7.9)	0.9<br>(0.4, 1.4)	-0.63<br>(-0.75, -0.34)	0.31<br>(0.12, 1.29)	**-0.046<br>(-0.061, -0.031)**	**<0.001**	123.3<br>(56.2, 202.5)	18.8<br>(8.6, 30.8)	-0.63<br>(-0.76, -0.34)	0.31<br>(0.12, 1.32)	**-1.006<br>(-1.342, -0.670)**	**<0.001**
Haiti	146.2<br>(101.0, 202.8)	2.5<br>(1.8, 3.4)	-0.36<br>(-0.55, 0.10)	0.18<br>(0.09, 0.33)	**-0.084<br>(-0.130, -0.039)**	**<0.001**	3916.8<br>(2694.0, 5500.9)	52.3<br>(36.2, 72.6)	-0.38<br>(-0.57, 0.07)	0.19<br>(0.09, 0.33)	**-1.862<br>(-2.828, -0.897)**	**<0.001**
Honduras	425.9<br>(362.8, 525.1)	8.3<br>(7.0, 10.1)	0.25<br>(0.01, 0.64)	0.03<br>(-0.03, 0.10)	0.077<br>(-0.014, 0.167)	0.093	9125.0<br>(7658.5, 11475.7)	151.7<br>(129.0, 188.3)	0.13<br>(-0.10, 0.51)	0.04<br>(-0.03, 0.11)	0.994<br>(-0.668, 2.655)	0.231
Hungary	5505.9<br>(4783.4, 6356.7)	26.0<br>(22.6, 30.1)	-0.42<br>(-0.48, -0.36)	0.09<br>(0.00, 0.17)	**-0.778<br>(-0.865, -0.691)**	**<0.001**	93439.5<br>(81543.9, 107839.9)	480.5<br>(417.7, 554.1)	-0.44<br>(-0.50, -0.38)	0.11<br>(0.01, 0.21)	**-15.935<br>(-17.697, -14.173)**	**<0.001**
Iceland	72.1<br>(58.8, 82.0)	10.5<br>(8.7, 11.8)	-0.57<br>(-0.60, -0.52)	-0.02<br>(-0.05, 0.01)	**-0.463<br>(-0.495, -0.431)**	**<0.001**	1011.0<br>(862.8, 1134.9)	163.5<br>(142.1, 183.9)	-0.61<br>(-0.65, -0.57)	-0.01<br>(-0.05, 0.03)	**-8.684<br>(-9.379, -7.988)**	**<0.001**
India	155236.9<br>(115658.0, 195460.1)	14.7<br>(10.9, 18.6)	-0.03<br>(-0.16, 0.13)	-0.14<br>(-0.21, -0.04)	0.001<br>(-0.060, 0.062)	0.967	3650036.4<br>(2790232.4, 4607706.8)	307.2<br>(232.7, 388.7)	-0.07<br>(-0.20, 0.08)	-0.13<br>(-0.22, -0.03)	-0.812<br>(-1.988, 0.364)	0.169
Indonesia	5915.6<br>(4730.6, 7343.1)	3.1<br>(2.5, 3.8)	-0.17<br>(-0.37, 0.17)	-0.01<br>(-0.20, 0.27)	**-0.031<br>(-0.051, -0.011)**	**0.004**	149470.6<br>(120569.6, 188374.2)	63.7<br>(51.2, 79.3)	-0.23<br>(-0.40, 0.09)	-0.00<br>(-0.21, 0.29)	**-0.808<br>(-1.221, -0.395)**	**<0.001**
Iran (Islamic Republic of)	15256.9<br>(13439.3, 16886.3)	22.9<br>(20.0, 25.4)	-0.43<br>(-0.47, -0.38)	-0.17<br>(-0.20, -0.14)	**-0.628<br>(-0.708, -0.547)**	**<0.001**	303839.3<br>(275693.7, 336873.1)	409.9<br>(367.2, 453.6)	-0.47<br>(-0.50, -0.42)	-0.18<br>(-0.21, -0.14)	**-12.869<br>(-14.387, -11.350)**	**<0.001**
Iraq	8822.2<br>(6333.4, 12049.6)	47.6<br>(34.8, 64.1)	-0.20<br>(-0.37, -0.03)	-0.09<br>(-0.17, -0.04)	**-0.534<br>(-0.742, -0.326)**	**<0.001**	208427.4<br>(147176.9, 293383.7)	905.3<br>(647.8, 1239.9)	-0.31<br>(-0.47, -0.14)	-0.12<br>(-0.18, -0.07)	**-16.280<br>(-20.567, -11.992)**	**<0.001**
Ireland	817.1<br>(691.7, 918.8)	9.6<br>(8.1, 10.7)	-0.69<br>(-0.71, -0.66)	-0.16<br>(-0.19, -0.14)	**-0.727<br>(-0.796, -0.659)**	**<0.001**	12076.3<br>(10572.4, 13445.6)	148.7<br>(131.0, 164.7)	-0.72<br>(-0.74, -0.70)	-0.17<br>(-0.19, -0.14)	**-13.465<br>(-14.715, -12.214)**	**<0.001**
Israel	677.2<br>(549.4, 766.4)	4.9<br>(4.0, 5.5)	-0.75<br>(-0.77, -0.73)	-0.20<br>(-0.22, -0.17)	**-0.517<br>(-0.575, -0.458)**	**<0.001**	9982.4<br>(8498.7, 11165.6)	77.4<br>(66.5, 86.3)	-0.77<br>(-0.79, -0.75)	-0.21<br>(-0.24, -0.18)	**-9.158<br>(-10.249, -8.068)**	**<0.001**
Italy	22953.0<br>(18422.1, 25543.0)	11.5<br>(9.5, 12.6)	-0.55<br>(-0.58, -0.53)	0.01<br>(-0.00, 0.02)	**-0.500<br>(-0.546, -0.454)**	**<0.001**	288330.5<br>(240956.5, 315128.7)	169.0<br>(146.3, 182.7)	-0.60<br>(-0.62, -0.58)	0.00<br>(-0.01, 0.01)	**-8.940<br>(-9.768, -8.111)**	**<0.001**
Jamaica	21.6<br>(14.7, 29.9)	0.6<br>(0.4, 0.9)	-0.60<br>(-0.69, -0.50)	0.34<br>(0.12, 0.57)	**-0.041<br>(-0.055, -0.026)**	**<0.001**	413.4<br>(280.7, 579.7)	13.1<br>(8.9, 18.5)	-0.58<br>(-0.68, -0.46)	0.34<br>(0.11, 0.59)	**-0.784<br>(-1.081, -0.487)**	**<0.001**
Japan	33149.4<br>(26110.9, 37338.3)	6.4<br>(5.4, 7.0)	-0.63<br>(-0.65, -0.62)	-0.04<br>(-0.05, -0.03)	**-0.360<br>(-0.403, -0.318)**	**<0.001**	437070.1<br>(364447.9, 481159.4)	113.5<br>(101.3, 122.0)	-0.61<br>(-0.62, -0.60)	-0.04<br>(-0.05, -0.03)	**-5.937<br>(-6.531, -5.343)**	**<0.001**
Jordan	960.8<br>(780.0, 1176.3)	17.4<br>(14.3, 21.0)	-0.54<br>(-0.63, -0.43)	-0.08<br>(-0.14, -0.01)	**-0.799<br>(-0.904, -0.694)**	**<0.001**	22530.5<br>(18106.7, 27847.3)	316.8<br>(257.7, 387.8)	-0.58<br>(-0.67, -0.47)	-0.08<br>(-0.15, -0.01)	**-17.476<br>(-19.468, -15.483)**	**<0.001**
Kazakhstan	5954.6<br>(5381.2, 6636.6)	41.7<br>(37.9, 46.3)	-0.05<br>(-0.15, 0.05)	-0.02<br>(-0.10, 0.05)	**-0.539<br>(-0.758, -0.319)**	**<0.001**	126538.6<br>(113821.0, 142225.9)	760.5<br>(686.7, 850.1)	-0.12<br>(-0.22, -0.02)	-0.02<br>(-0.11, 0.06)	**-13.918<br>(-19.240, -8.596)**	**<0.001**

Kenya	2459.3<br>(2015.4, 2959.0)	13.9<br>(11.3, 16.8)	-0.05<br>(-0.21, 0.15)	0.09<br>(-0.11, 0.36)	0.014<br>(-0.019, 0.048)	0.391	60914.9<br>(50027.3, 73165.4)	268.9<br>(220.1, 323.8)	-0.05<br>(-0.22, 0.17)	0.09<br>(-0.11, 0.36)	0.339<br>(-0.396, 1.074)	0.354
Kiribati	0.3<br>(0.1, 0.5)	0.6<br>(0.2, 0.9)	8.03<br>(3.62, 11.51)	43.42<br>(24.97, 52.56)	-0.005<br>(-0.029, 0.018)	0.646	10.6<br>(3.3, 16.7)	13.1<br>(4.2, 20.9)	7.83<br>(3.58, 11.60)	43.12<br>(24.91, 52.37)	-0.130<br>(-0.687, 0.427)	0.636
Kuwait	425.2<br>(293.2, 577.7)	17.1<br>(12.0, 23.1)	-0.54<br>(-0.62, -0.45)	-0.12<br>(-0.25, 0.02)	**-0.646<br>(-0.793, -0.499)**	**<0.001**	10638.7<br>(7345.5, 14493.7)	328.1<br>(225.3, 444.9)	-0.57<br>(-0.65, -0.48)	-0.13<br>(-0.27, 0.03)	**-14.267<br>(-17.322, -11.213)**	**<0.001**
Kyrgyzstan	1391.2<br>(1188.2, 1605.9)	35.5<br>(30.3, 41.1)	-0.33<br>(-0.43, -0.23)	-0.04<br>(-0.13, 0.05)	**-0.846<br>(-1.025, -0.666)**	**<0.001**	31562.2<br>(26795.5, 36722.5)	681.7<br>(580.6, 790.1)	-0.36<br>(-0.46, -0.25)	-0.03<br>(-0.13, 0.08)	**-20.441<br>(-24.933, -15.949)**	**<0.001**
Lao People's Democratic Republic	489.3<br>(385.5, 611.0)	12.8<br>(10.2, 15.8)	-0.27<br>(-0.43, -0.05)	0.41<br>(0.31, 0.50)	**-0.283<br>(-0.366, -0.201)**	**<0.001**	12499.7<br>(9631.1, 15926.0)	264.5<br>(207.2, 331.3)	-0.34<br>(-0.49, -0.11)	0.41<br>(0.30, 0.51)	**-7.204<br>(-9.031, -5.377)**	**<0.001**
Latvia	1215.4<br>(1061.3, 1357.8)	26.9<br>(23.6, 30.0)	-0.38<br>(-0.45, -0.31)	0.05<br>(-0.04, 0.14)	**-0.843<br>(-0.964, -0.722)**	**<0.001**	19735.4<br>(17372.0, 22098.7)	503.8<br>(447.1, 565.9)	-0.38<br>(-0.46, -0.31)	0.07<br>(-0.04, 0.17)	**-17.448<br>(-20.387, -14.509)**	**<0.001**
Lebanon	1033.0<br>(859.3, 1199.7)	15.7<br>(13.1, 18.2)	-0.63<br>(-0.70, -0.56)	-0.10<br>(-0.16, -0.02)	**-0.879<br>(-0.994, -0.763)**	**<0.001**	17180.9<br>(14459.6, 20097.1)	275.0<br>(231.6, 322.5)	-0.68<br>(-0.74, -0.60)	-0.09<br>(-0.16, -0.02)	**-18.351<br>(-20.976, -15.726)**	**<0.001**
Lesotho	493.8<br>(391.6, 616.2)	54.6<br>(43.8, 67.0)	0.38<br>(0.06, 0.78)	0.13<br>(0.06, 0.20)	**0.735<br>(0.492, 0.978)**	**<0.001**	12319.4<br>(9633.1, 15630.9)	1146.8<br>(903.8, 1437.4)	0.48<br>(0.12, 0.97)	0.17<br>(0.09, 0.25)	**16.825<br>(11.587, 22.064)**	**<0.001**
Liberia	20.1<br>(11.6, 31.7)	1.2<br>(0.7, 1.8)	-0.50<br>(-0.68, -0.15)	-0.07<br>(-0.16, 0.47)	**-0.034<br>(-0.049, -0.019)**	**<0.001**	546.6<br>(309.7, 872.1)	23.5<br>(13.6, 37.1)	-0.52<br>(-0.70, -0.17)	-0.06<br>(-0.16, 0.47)	**-0.752<br>(-1.060, -0.444)**	**<0.001**
Libya	924.7<br>(709.5, 1182.4)	20.6<br>(16.0, 26.3)	0.01<br>(-0.21, 0.31)	-0.14<br>(-0.20, -0.08)	**0.112<br>(0.048, 0.175)**	**0.001**	22724.9<br>(17340.4, 29350.5)	423.3<br>(325.7, 540.3)	-0.00<br>(-0.23, 0.32)	-0.15<br>(-0.21, -0.08)	**1.631<br>(0.379, 2.884)**	**0.012**
Lithuania	1579.1<br>(1393.6, 1739.9)	23.8<br>(21.2, 26.1)	-0.33<br>(-0.40, -0.26)	0.08<br>(-0.02, 0.17)	**-0.582<br>(-0.680, -0.484)**	**<0.001**	24836.2<br>(22135.0, 27291.6)	426.5<br>(380.2, 468.9)	-0.36<br>(-0.43, -0.28)	0.08<br>(-0.03, 0.18)	**-11.850<br>(-13.993, -9.706)**	**<0.001**
Luxembourg	124.7<br>(107.9, 139.7)	10.2<br>(8.9, 11.4)	-0.63<br>(-0.67, -0.59)	-0.02<br>(-0.07, 0.02)	**-0.600<br>(-0.659, -0.541)**	**<0.001**	1770.8<br>(1569.9, 1974.4)	156.9<br>(139.4, 175.4)	-0.66<br>(-0.70, -0.63)	-0.02<br>(-0.07, 0.03)	**-10.814<br>(-11.845, -9.784)**	**<0.001**
Madagascar	1875.4<br>(1431.8, 2380.4)	21.6<br>(16.8, 27.0)	-0.14<br>(-0.33, 0.09)	0.12<br>(0.05, 0.20)	**-0.204<br>(-0.245, -0.164)**	**<0.001**	54072.3<br>(41256.0, 69262.0)	449.1<br>(343.7, 567.1)	-0.17<br>(-0.36, 0.07)	0.11<br>(0.04, 0.19)	**-4.949<br>(-5.818, -4.080)**	**<0.001**
Malawi	860.4<br>(718.9, 1036.4)	14.0<br>(11.8, 16.6)	0.16<br>(-0.03, 0.55)	0.13<br>(0.04, 0.20)	-0.033<br>(-0.097, 0.030)	0.296	23265.6<br>(19072.0, 28297.7)	291.1<br>(243.8, 349.9)	0.14<br>(-0.05, 0.55)	0.13<br>(0.05, 0.21)	-0.975<br>(-2.407, 0.457)	0.175
Malaysia	175.7<br>(143.5, 218.1)	0.7<br>(0.6, 0.9)	-0.49<br>(-0.54, -0.31)	0.76<br>(0.63, 0.89)	**-0.038<br>(-0.046, -0.029)**	**<0.001**	4053.1<br>(3327.4, 5047.0)	14.4<br>(11.8, 17.9)	-0.51<br>(-0.56, -0.34)	0.75<br>(0.63, 0.87)	**-0.801<br>(-0.973, -0.628)**	**<0.001**
Maldives	0.1<br>(0.0, 0.2)	0.0<br>(0.0, 0.1)	-0.89<br>(-0.97, -0.76)	-0.15<br>(-0.35, -0.02)	**-0.010<br>(-0.012, -0.008)**	**<0.001**	1.5<br>(0.0, 4.0)	0.4<br>(-0.0, 1.1)	-0.91<br>(-0.99, -0.81)	-0.16<br>(-0.38, 0.04)	**-0.214<br>(-0.261, -0.167)**	**<0.001**

Mali	142.1<br>(88.4, 224.9)	1.9<br>(1.2, 3.0)	-0.73<br>(-0.78, -0.65)	-0.62<br>(-0.65, -0.54)	**-0.086<br>(-0.157, -0.014)**	**0.021**	3887.2<br>(2400.0, 6204.0)	39.9<br>(24.9, 63.1)	-0.74<br>(-0.80, -0.66)	-0.62<br>(-0.65, -0.54)	**-2.006<br>(-3.514, -0.499)**	**0.011**
Malta	95.0<br>(78.1, 110.8)	8.5<br>(7.1, 10.0)	-0.64<br>(-0.68, -0.61)	-0.09<br>(-0.13, -0.07)	**-0.529<br>(-0.564, -0.493)**	**<0.001**	1423.0<br>(1210.1, 1646.6)	143.2<br>(124.0, 164.8)	-0.66<br>(-0.69, -0.63)	-0.10<br>(-0.14, -0.07)	**-9.536<br>(-10.182, -8.890)**	**<0.001**
Marshall Islands	0.0<br>(0.0, 0.0)	0.0<br>(0.0, 0.0)	-0.84<br>(-0.88, -0.79)	0.37<br>(0.10, 0.73)	**-0.005<br>(-0.008, -0.003)**	**<0.001**	0.2<br>(0.1, 0.2)	0.4<br>(0.3, 0.6)	-0.84<br>(-0.88, -0.78)	0.37<br>(0.10, 0.73)	**-0.126<br>(-0.179, -0.073)**	**<0.001**
Mauritania	94.7<br>(62.4, 133.6)	5.2<br>(3.4, 7.2)	-0.62<br>(-0.70, -0.51)	-0.49<br>(-0.53, -0.44)	**-0.137<br>(-0.237, -0.037)**	**0.009**	2155.7<br>(1424.2, 3049.1)	99.4<br>(65.5, 140.6)	-0.65<br>(-0.73, -0.54)	-0.49<br>(-0.53, -0.44)	**-3.240<br>(-5.241, -1.239)**	**0.002**
Mauritius	125.4<br>(106.1, 150.1)	7.3<br>(6.2, 8.6)	-0.50<br>(-0.59, -0.43)	0.21<br>(0.14, 0.26)	**-0.338<br>(-0.386, -0.289)**	**<0.001**	2759.6<br>(2336.2, 3331.5)	156.7<br>(133.5, 188.4)	-0.49<br>(-0.58, -0.41)	0.20<br>(0.11, 0.26)	**-7.002<br>(-8.046, -5.959)**	**<0.001**
Mexico	19685.6<br>(17453.3, 22319.8)	16.8<br>(14.9, 19.0)	-0.24<br>(-0.31, -0.15)	0.06<br>(-0.05, 0.16)	**-0.168<br>(-0.207, -0.130)**	**<0.001**	425330.2<br>(375647.7, 488009.1)	338.0<br>(298.3, 385.9)	-0.17<br>(-0.26, -0.07)	0.07<br>(-0.04, 0.19)	**-2.460<br>(-3.171, -1.750)**	**<0.001**
Micronesia (Federated States of)	0.0<br>(0.0, 0.0)	0.0<br>(0.0, 0.0)	-0.72<br>(-0.82, -0.62)	0.71<br>(0.24, 1.32)	**-0.004<br>(-0.006, -0.003)**	**<0.001**	0.4<br>(0.3, 0.6)	0.5<br>(0.3, 0.8)	-0.72<br>(-0.83, -0.61)	0.71<br>(0.24, 1.32)	**-0.100<br>(-0.137, -0.064)**	**<0.001**
Monaco	13.8<br>(11.0, 16.5)	11.3<br>(9.1, 13.4)	-0.48<br>(-0.58, -0.34)	-0.01<br>(-0.05, 0.03)	**-0.400<br>(-0.436, -0.365)**	**<0.001**	189.3<br>(153.4, 225.6)	183.0<br>(149.8, 221.0)	-0.51<br>(-0.61, -0.35)	-0.01<br>(-0.05, 0.04)	**-7.086<br>(-7.695, -6.477)**	**<0.001**
Mongolia	643.9<br>(552.8, 748.3)	36.3<br>(31.3, 42.1)	-0.34<br>(-0.42, -0.25)	0.01<br>(-0.10, 0.11)	**-0.966<br>(-1.107, -0.826)**	**<0.001**	15739.6<br>(13439.0, 18337.5)	702.1<br>(603.1, 816.6)	-0.37<br>(-0.44, -0.27)	-0.01<br>(-0.12, 0.10)	**-20.233<br>(-23.261, -17.205)**	**<0.001**
Montenegro	380.0<br>(341.4, 428.6)	44.8<br>(40.3, 50.2)	0.24<br>(0.12, 0.38)	0.06<br>(-0.00, 0.12)	**0.154<br>(0.053, 0.254)**	**0.004**	6443.7<br>(5736.3, 7300.6)	710.2<br>(635.2, 802.9)	0.05<br>(-0.07, 0.18)	0.03<br>(-0.03, 0.11)	**-1.965<br>(-3.478, -0.452)**	**0.013**
Morocco	11556.6<br>(9227.7, 13411.7)	39.6<br>(31.8, 45.5)	-0.11<br>(-0.29, 0.03)	-0.03<br>(-0.09, 0.06)	**-0.204<br>(-0.319, -0.089)**	**0.001**	246506.4<br>(194403.6, 290279.4)	747.2<br>(595.2, 870.4)	-0.19<br>(-0.37, -0.04)	-0.03<br>(-0.09, 0.06)	**-6.897<br>(-9.180, -4.614)**	**<0.001**
Mozambique	824.6<br>(615.3, 1055.9)	9.1<br>(6.9, 11.6)	0.18<br>(-0.10, 0.48)	0.17<br>(0.07, 0.32)	**0.041<br>(0.013, 0.070)**	**0.006**	22772.2<br>(16861.9, 29814.5)	190.8<br>(142.4, 245.0)	0.20<br>(-0.09, 0.50)	0.17<br>(0.07, 0.33)	**1.002<br>(0.372, 1.632)**	**0.003**
Myanmar	3095.0<br>(2461.7, 3933.2)	7.5<br>(6.0, 9.6)	-0.53<br>(-0.64, -0.37)	-0.24<br>(-0.29, -0.18)	**-0.299<br>(-0.387, -0.211)**	**<0.001**	70965.6<br>(55257.0, 90156.3)	149.7<br>(117.6, 190.1)	-0.58<br>(-0.68, -0.43)	-0.25<br>(-0.30, -0.19)	**-7.408<br>(-9.329, -5.487)**	**<0.001**
Namibia	209.0<br>(164.6, 257.1)	19.1<br>(15.3, 23.0)	-0.07<br>(-0.26, 0.16)	0.55<br>(0.47, 0.66)	**-0.212<br>(-0.343, -0.082)**	**0.002**	4893.5<br>(3739.9, 6195.9)	370.5<br>(289.4, 459.7)	-0.09<br>(-0.29, 0.15)	0.58<br>(0.48, 0.69)	**-4.606<br>(-7.269, -1.943)**	**0.001**
Nauru	0.0<br>(0.0, 0.0)	0.1<br>(0.0, 0.1)	-0.71<br>(-0.77, -0.64)	0.04<br>(-0.03, 0.15)	**-0.009<br>(-0.013, -0.006)**	**<0.001**	0.1<br>(0.1, 0.1)	1.3<br>(0.8, 1.7)	-0.72<br>(-0.78, -0.63)	0.04<br>(-0.04, 0.14)	**-0.229<br>(-0.312, -0.147)**	**<0.001**
Nepal	5300.8<br>(4338.9, 6473.2)	27.1<br>(22.3, 33.0)	-0.13<br>(-0.31, 0.14)	-0.06<br>(-0.12, 0.01)	-0.045<br>(-0.137, 0.048)	0.334	116430.1<br>(94043.8, 145087.5)	519.2<br>(423.5, 642.5)	-0.19<br>(-0.39, 0.07)	-0.06<br>(-0.12, 0.00)	**-2.391<br>(-4.292, -0.490)**	**0.015**

Netherlands	4160.2<br>(3531.4, 4682.1)	10.4<br>(8.9, 11.7)	-0.50<br>(-0.54, -0.47)	0.05<br>(0.03, 0.06)	**-0.461<br>(-0.511, -0.411)**	**<0.001**	58412.9<br>(51227.0, 65162.9)	157.7<br>(139.8, 174.9)	-0.58<br>(-0.61, -0.54)	0.02<br>(0.01, 0.04)	**-8.946<br>(-9.844, -8.049)**	**<0.001**
New Zealand	1026.7<br>(885.3, 1180.4)	11.2<br>(9.7, 12.8)	-0.57<br>(-0.60, -0.54)	-0.06<br>(-0.08, -0.04)	**-0.592<br>(-0.645, -0.539)**	**<0.001**	15913.7<br>(14200.9, 18170.4)	187.1<br>(168.3, 213.2)	-0.61<br>(-0.64, -0.59)	-0.06<br>(-0.08, -0.04)	**-11.660<br>(-12.813, -10.508)**	**<0.001**
Nicaragua	67.7<br>(55.2, 82.0)	1.6<br>(1.3, 1.9)	0.16<br>(-0.15, 0.63)	-0.07<br>(-0.18, 0.07)	0.010<br>(-0.015, 0.035)	0.430	1508.1<br>(1220.2, 1850.5)	30.7<br>(25.0, 37.6)	0.13<br>(-0.19, 0.61)	-0.06<br>(-0.18, 0.10)	0.131<br>(-0.368, 0.631)	0.595
Niger	453.9<br>(279.4, 676.0)	6.9<br>(4.3, 10.0)	-0.08<br>(-0.31, 0.42)	-0.28<br>(-0.32, -0.24)	**-0.111<br>(-0.194, -0.027)**	**0.011**	12212.7<br>(7345.0, 18680.6)	137.1<br>(84.6, 203.7)	-0.14<br>(-0.36, 0.36)	-0.28<br>(-0.32, -0.24)	**-2.754<br>(-4.499, -1.008)**	**0.003**
Nigeria	1544.6<br>(1018.6, 2132.7)	2.0<br>(1.3, 2.7)	-0.46<br>(-0.57, -0.29)	-0.22<br>(-0.36, -0.04)	**-0.094<br>(-0.122, -0.065)**	**<0.001**	42224.4<br>(26964.4, 59542.7)	40.6<br>(26.7, 56.5)	-0.47<br>(-0.59, -0.27)	-0.22<br>(-0.38, -0.01)	**-2.101<br>(-2.729, -1.473)**	**<0.001**
Niue	0.1<br>(0.1, 0.1)	5.0<br>(4.1, 6.0)	-0.29<br>(-0.41, -0.12)	-0.04<br>(-0.07, -0.01)	**-0.114<br>(-0.169, -0.060)**	**<0.001**	2.2<br>(1.8, 2.7)	107.4<br>(88.2, 131.5)	-0.28<br>(-0.42, -0.08)	-0.02<br>(-0.06, 0.02)	**-2.517<br>(-3.680, -1.355)**	**<0.001**
North Macedonia	1284.4<br>(1083.7, 1505.5)	53.1<br>(46.2, 61.1)	0.01<br>(-0.12, 0.14)	0.00<br>(-0.11, 0.13)	**-0.426<br>(-0.738, -0.114)**	**0.009**	23011.8<br>(19078.3, 27533.2)	813.1<br>(687.8, 955.2)	-0.17<br>(-0.30, -0.05)	0.03<br>(-0.11, 0.19)	**-12.819<br>(-16.745, -8.893)**	**<0.001**
Northern Mariana Islands	0.8<br>(0.6, 0.9)	1.8<br>(1.5, 2.1)	-0.37<br>(-0.50, -0.17)	-0.15<br>(-0.26, -0.02)	-0.016<br>(-0.036, 0.005)	0.133	19.8<br>(16.6, 23.1)	38.3<br>(32.2, 44.6)	-0.38<br>(-0.51, -0.18)	-0.15<br>(-0.26, -0.02)	-0.330<br>(-0.767, 0.106)	0.132
Norway	1125.6<br>(955.4, 1229.4)	9.5<br>(8.1, 10.3)	-0.56<br>(-0.60, -0.53)	-0.05<br>(-0.07, -0.03)	**-0.463<br>(-0.492, -0.435)**	**<0.001**	15923.4<br>(14041.0, 17133.7)	147.8<br>(132.2, 158.1)	-0.62<br>(-0.65, -0.59)	-0.06<br>(-0.08, -0.04)	**-8.842<br>(-9.373, -8.311)**	**<0.001**
Oman	326.1<br>(230.8, 439.3)	22.8<br>(16.1, 30.6)	-0.49<br>(-0.61, -0.31)	-0.29<br>(-0.34, -0.24)	**-0.548<br>(-0.737, -0.360)**	**<0.001**	8306.5<br>(5871.8, 11325.8)	421.8<br>(298.0, 567.0)	-0.55<br>(-0.67, -0.38)	-0.31<br>(-0.36, -0.26)	**-14.796<br>(-18.270, -11.322)**	**<0.001**
Pakistan	34908.8<br>(26879.4, 46271.9)	34.3<br>(26.5, 45.0)	0.09<br>(-0.10, 0.38)	-0.17<br>(-0.33, 0.05)	0.066<br>(-0.048, 0.181)	0.246	918397.6<br>(691201.7, 1217951.0)	715.4<br>(548.1, 949.2)	0.08<br>(-0.12, 0.38)	-0.17<br>(-0.35, 0.07)	1.101<br>(-1.377, 3.578)	0.372
Palau	0.0<br>(0.0, 0.0)	0.2<br>(0.1, 0.2)	-0.54<br>(-0.66, -0.16)	0.38<br>(0.18, 0.70)	**-0.012<br>(-0.016, -0.007)**	**<0.001**	0.7<br>(0.4, 1.0)	3.3<br>(1.9, 4.9)	-0.55<br>(-0.67, -0.17)	0.38<br>(0.19, 0.68)	**-0.260<br>(-0.355, -0.164)**	**<0.001**
Palestine	423.1<br>(363.3, 494.4)	23.0<br>(19.7, 26.6)	-0.47<br>(-0.56, -0.35)	-0.12<br>(-0.17, -0.06)	**-0.681<br>(-0.804, -0.559)**	**<0.001**	9493.7<br>(8133.1, 11080.1)	402.7<br>(346.1, 470.1)	-0.49<br>(-0.59, -0.36)	-0.13<br>(-0.18, -0.07)	**-13.243<br>(-15.279, -11.207)**	**<0.001**
Panama	43.9<br>(33.0, 53.7)	1.0<br>(0.7, 1.2)	-0.25<br>(-0.43, -0.07)	0.25<br>(0.02, 0.46)	**-0.022<br>(-0.029, -0.015)**	**<0.001**	827.5<br>(626.6, 1014.7)	18.6<br>(14.1, 22.8)	-0.24<br>(-0.43, -0.04)	0.27<br>(0.03, 0.50)	**-0.418<br>(-0.554, -0.281)**	**<0.001**
Papua New Guinea	976.6<br>(753.6, 1216.1)	24.9<br>(19.5, 30.5)	-0.23<br>(-0.42, 0.01)	-0.08<br>(-0.17, -0.02)	**-0.235<br>(-0.292, -0.179)**	**<0.001**	27202.9<br>(20873.7, 33959.2)	518.6<br>(401.6, 644.0)	-0.25<br>(-0.45, 0.01)	-0.09<br>(-0.17, -0.02)	**-5.535<br>(-6.736, -4.335)**	**<0.001**
Paraguay	568.2<br>(427.6, 738.6)	10.4<br>(7.9, 13.5)	-0.26<br>(-0.42, -0.05)	0.04<br>(-0.16, 0.30)	-0.050<br>(-0.106, 0.006)	0.078	12030.8<br>(9043.4, 15654.6)	206.7<br>(155.3, 268.9)	-0.26<br>(-0.43, -0.04)	0.07<br>(-0.16, 0.36)	**-1.222<br>(-2.323, -0.120)**	**0.031**

Peru	3005.2 (2381.6, 3687.2)	8.9 (7.1, 11.0)	-0.33 (-0.48, -0.15)	-0.05 (-0.22, 0.13)	**-0.164 (-0.203, -0.125)**	**<0.001**	61843.9 (49063.9, 76394.2)	181.0 (143.6, 223.7)	-0.36 (-0.51, -0.18)	-0.04 (-0.22, 0.16)	**-3.656 (-4.498, -2.814)**	**<0.001**
Philippines	854.7 (663.0, 1065.3)	1.2 (0.9, 1.5)	-0.27 (-0.41, 0.06)	0.14 (-0.03, 0.31)	**-0.035 (-0.053, -0.016)**	**<0.001**	22109.1 (16878.6, 27933.2)	26.0 (20.0, 32.5)	-0.21 (-0.37, 0.20)	0.14 (-0.03, 0.34)	**-0.662 (-1.057, -0.268)**	**0.002**
Poland	14525.8 (12790.0, 15922.7)	18.8 (16.6, 20.6)	-0.54 (-0.58, -0.50)	0.12 (0.04, 0.20)	**-0.921 (-1.000, -0.841)**	**<0.001**	236628.6 (212624.8, 260299.2)	328.2 (295.7, 361.0)	-0.58 (-0.61, -0.54)	0.11 (0.03, 0.20)	**-18.227 (-19.880, -16.575)**	**<0.001**
Portugal	3921.9 (3297.1, 4345.6)	12.5 (10.7, 13.8)	-0.67 (-0.69, -0.65)	-0.05 (-0.07, -0.04)	**-0.969 (-1.055, -0.883)**	**<0.001**	53118.5 (46029.4, 58175.6)	197.8 (175.5, 215.4)	-0.68 (-0.70, -0.66)	-0.06 (-0.08, -0.05)	**-16.087 (-17.573, -14.601)**	**<0.001**
Puerto Rico	15.5 (10.0, 22.5)	0.2 (0.1, 0.3)	-0.84 (-0.88, -0.80)	-0.13 (-0.26, 0.01)	**-0.029 (-0.037, -0.022)**	**<0.001**	250.9 (154.9, 364.7)	3.7 (2.2, 5.3)	-0.83 (-0.88, -0.78)	-0.13 (-0.27, 0.02)	**-0.551 (-0.693, -0.408)**	**<0.001**
Qatar	102.0 (69.7, 145.4)	20.3 (13.9, 28.0)	-0.73 (-0.79, -0.65)	-0.00 (-0.07, 0.08)	**-2.006 (-2.311, -1.702)**	**<0.001**	2978.3 (2018.5, 4282.5)	338.8 (233.5, 477.1)	-0.74 (-0.81, -0.66)	-0.07 (-0.15, 0.01)	**-36.513 (-41.481, -31.545)**	**<0.001**
Republic of Korea	8474.9 (6977.2, 9757.8)	9.4 (7.7, 10.8)	-0.72 (-0.76, -0.69)	-0.03 (-0.05, -0.01)	**-0.875 (-0.932, -0.818)**	**<0.001**	130681.3 (111745.2, 148960.7)	145.2 (124.2, 165.7)	-0.76 (-0.79, -0.73)	-0.04 (-0.06, -0.02)	**-16.252 (-17.404, -15.100)**	**<0.001**
Republic of Moldova	1796.8 (1555.3, 2096.0)	29.8 (25.8, 34.8)	-0.34 (-0.40, -0.28)	0.16 (0.07, 0.25)	**-1.117 (-1.287, -0.948)**	**<0.001**	34566.9 (30003.1, 40014.9)	586.2 (508.9, 677.7)	-0.29 (-0.35, -0.22)	0.16 (0.06, 0.26)	**-18.849 (-22.183, -15.516)**	**<0.001**
Romania	13337.1 (11800.8, 14789.8)	32.9 (29.2, 36.5)	-0.41 (-0.46, -0.36)	0.15 (0.04, 0.24)	**-1.143 (-1.266, -1.020)**	**<0.001**	220989.2 (197008.2, 244989.9)	591.9 (526.4, 656.9)	-0.39 (-0.45, -0.34)	0.15 (0.03, 0.25)	**-20.551 (-23.030, -18.071)**	**<0.001**
Russian Federation	73662.8 (66359.7, 82168.9)	30.8 (27.8, 34.3)	-0.37 (-0.42, -0.31)	-0.02 (-0.09, 0.06)	**-0.956 (-1.198, -0.715)**	**<0.001**	1390388.4 (1267076.9, 1551866.0)	600.8 (546.7, 669.4)	-0.33 (-0.40, -0.27)	-0.02 (-0.09, 0.06)	**-17.983 (-23.634, -12.331)**	**<0.001**
Rwanda	866.7 (672.3, 1109.8)	17.9 (14.0, 22.8)	-0.54 (-0.64, -0.41)	0.02 (-0.06, 0.10)	**-1.041 (-1.182, -0.899)**	**<0.001**	21485.1 (16361.7, 27682.4)	344.8 (267.5, 441.8)	-0.60 (-0.69, -0.47)	0.01 (-0.06, 0.09)	**-24.969 (-28.357, -21.580)**	**<0.001**
Saint Kitts and Nevis	0.3 (0.2, 0.4)	0.5 (0.3, 0.7)	-0.75 (-0.80, -0.71)	-0.23 (-0.30, -0.19)	**-0.034 (-0.047, -0.022)**	**<0.001**	6.6 (4.0, 9.1)	10.0 (6.1, 13.9)	-0.76 (-0.81, -0.71)	-0.23 (-0.30, -0.18)	**-0.731 (-0.985, -0.477)**	**<0.001**
Saint Lucia	0.4 (0.2, 0.6)	0.2 (0.1, 0.2)	-0.82 (-0.88, -0.64)	-0.26 (-0.33, -0.08)	**-0.015 (-0.021, -0.009)**	**<0.001**	7.6 (3.1, 11.2)	3.3 (1.4, 4.8)	-0.80 (-0.86, -0.60)	-0.28 (-0.35, -0.10)	**-0.259 (-0.369, -0.150)**	**<0.001**
Saint Vincent and the Grenadines	0.2 (0.1, 0.2)	0.1 (0.1, 0.2)	-0.80 (-0.85, -0.50)	-0.18 (-0.26, -0.06)	**-0.012 (-0.016, -0.007)**	**<0.001**	3.1 (1.5, 4.8)	2.3 (1.2, 3.6)	-0.81 (-0.86, -0.54)	-0.17 (-0.26, -0.03)	**-0.216 (-0.303, -0.128)**	**<0.001**
Samoa	3.5 (2.6, 4.4)	2.7 (2.0, 3.5)	-0.04 (-0.24, 1.23)	0.71 (0.63, 0.79)	**-0.037 (-0.065, -0.010)**	**0.010**	85.0 (62.8, 110.8)	58.2 (43.2, 75.1)	-0.02 (-0.25, 1.28)	0.71 (0.62, 0.81)	**-0.727 (-1.308, -0.147)**	**0.016**
San Marino	6.5 (4.6, 8.6)	6.1 (4.2, 8.3)	-0.64 (-0.74, -0.52)	-0.35 (-0.48, -0.23)	**-0.340 (-0.383, -0.296)**	**<0.001**	84.1 (57.1, 114.1)	97.3 (65.6, 134.8)	-0.64 (-0.75, -0.50)	-0.33 (-0.48, -0.19)	**-5.384 (-6.026, -4.742)**	**<0.001**

Sao Tome and Principe	0.7 (0.5, 0.8)	0.7 (0.5, 0.9)	-0.64 (-0.74, -0.41)	-0.32 (-0.39, -0.25)	-0.006 (-0.031, 0.019)	0.617	15.9 (11.4, 20.2)	14.0 (10.0, 17.6)	-0.65 (-0.75, -0.41)	-0.32 (-0.38, -0.24)	-0.193 (-0.698, 0.312)	0.442
Saudi Arabia	4067.1 (2937.4, 5380.7)	25.8 (19.0, 33.4)	-0.35 (-0.49, -0.15)	-0.14 (-0.18, -0.10)	**-0.559 (-0.697, -0.420)**	**<0.001**	128675.3 (91015.4, 172030.2)	543.1 (396.2, 715.6)	-0.34 (-0.49, -0.10)	-0.14 (-0.18, -0.10)	**-10.436 (-13.365, -7.508)**	**<0.001**
Senegal	155.3 (105.8, 211.4)	2.4 (1.6, 3.2)	-0.57 (-0.67, -0.42)	-0.48 (-0.51, -0.43)	-0.024 (-0.081, 0.034)	0.405	3718.1 (2502.2, 5123.7)	47.0 (32.0, 64.1)	-0.60 (-0.70, -0.45)	-0.48 (-0.51, -0.43)	-0.662 (-1.843, 0.519)	0.261
Serbia	6585.3 (5721.2, 7614.9)	38.0 (33.0, 44.0)	-0.40 (-0.47, -0.32)	0.14 (0.01, 0.27)	**-1.428 (-1.649, -1.207)**	**<0.001**	105590.0 (91596.4, 121909.2)	628.9 (544.5, 725.1)	-0.40 (-0.48, -0.32)	0.14 (0.01, 0.27)	**-23.156 (-26.514, -19.797)**	**<0.001**
Seychelles	1.3 (1.0, 1.6)	1.3 (1.0, 1.6)	-0.47 (-0.52, -0.41)	1.11 (0.94, 1.35)	**-0.058 (-0.074, -0.041)**	**<0.001**	29.4 (22.6, 35.9)	25.9 (19.8, 31.7)	-0.51 (-0.56, -0.45)	1.08 (0.91, 1.32)	**-1.294 (-1.644, -0.944)**	**<0.001**
Sierra Leone	37.7 (21.5, 57.5)	1.2 (0.7, 1.8)	-0.48 (-0.64, -0.28)	-0.08 (-0.16, 0.16)	**-0.036 (-0.051, -0.022)**	**<0.001**	985.6 (543.0, 1526.7)	23.9 (13.5, 36.5)	-0.49 (-0.65, -0.28)	-0.08 (-0.17, 0.16)	**-0.761 (-1.062, -0.461)**	**<0.001**
Singapore	5.2 (2.3, 8.4)	0.1 (0.0, 0.1)	-0.79 (-0.89, -0.70)	0.91 (0.66, 1.47)	**-0.012 (-0.016, -0.009)**	**<0.001**	106.7 (48.5, 167.5)	1.3 (0.6, 2.0)	-0.80 (-0.88, -0.71)	0.88 (0.66, 1.37)	**-0.263 (-0.328, -0.197)**	**<0.001**
Slovakia	2173.6 (1903.5, 2443.4)	22.9 (20.0, 25.7)	-0.43 (-0.49, -0.37)	0.13 (0.02, 0.23)	**-0.653 (-0.710, -0.595)**	**<0.001**	36855.2 (32454.4, 41622.1)	391.6 (344.7, 443.3)	-0.49 (-0.55, -0.43)	0.13 (0.01, 0.25)	**-14.067 (-15.165, -12.969)**	**<0.001**
Slovenia	643.6 (539.7, 729.5)	11.8 (10.0, 13.3)	-0.61 (-0.66, -0.57)	0.04 (-0.05, 0.13)	**-0.728 (-0.789, -0.667)**	**<0.001**	8827.8 (7535.8, 9906.3)	181.0 (154.7, 203.0)	-0.67 (-0.71, -0.63)	0.04 (-0.07, 0.14)	**-14.165 (-15.304, -13.027)**	**<0.001**
Solomon Islands	1.9 (1.1, 3.0)	0.7 (0.4, 1.1)	-0.61 (-0.73, -0.40)	-0.04 (-0.11, 0.24)	**-0.067 (-0.084, -0.050)**	**<0.001**	53.9 (29.4, 86.6)	14.6 (8.1, 23.3)	-0.61 (-0.74, -0.36)	-0.05 (-0.13, 0.23)	**-1.462 (-1.840, -1.084)**	**<0.001**
Somalia	122.3 (86.5, 162.7)	2.5 (1.8, 3.3)	-0.15 (-0.35, 0.14)	0.06 (-0.01, 0.14)	**-0.030 (-0.044, -0.015)**	**<0.001**	3787.1 (2645.0, 5124.7)	53.9 (38.5, 71.3)	-0.16 (-0.37, 0.14)	0.08 (0.01, 0.16)	**-0.714 (-1.033, -0.395)**	**<0.001**
South Africa	10944.8 (10050.2, 11769.9)	27.8 (25.5, 29.9)	0.12 (0.03, 0.20)	0.17 (0.11, 0.22)	0.027 (-0.119, 0.173)	0.711	248236.6 (228410.1, 267626.8)	547.0 (503.2, 588.9)	0.04 (-0.04, 0.12)	0.18 (0.12, 0.25)	-0.447 (-3.360, 2.467)	0.756
South Sudan	83.9 (51.3, 126.4)	2.6 (1.6, 3.9)	-0.24 (-0.44, -0.01)	-0.05 (-0.13, 0.05)	**-0.116 (-0.155, -0.077)**	**<0.001**	2425.5 (1445.9, 3705.1)	55.7 (34.1, 83.9)	-0.24 (-0.45, 0.02)	-0.05 (-0.12, 0.06)	**-2.492 (-3.338, -1.646)**	**<0.001**
Spain	13470.5 (11191.1, 14831.4)	10.3 (8.8, 11.2)	-0.59 (-0.61, -0.57)	-0.04 (-0.06, -0.03)	**-0.521 (-0.572, -0.470)**	**<0.001**	177326.3 (153883.2, 192098.2)	160.3 (143.2, 172.1)	-0.61 (-0.63, -0.60)	-0.04 (-0.06, -0.03)	**-8.993 (-9.874, -8.112)**	**<0.001**
Sri Lanka	578.2 (411.0, 786.6)	2.4 (1.7, 3.2)	-0.41 (-0.58, -0.20)	0.01 (-0.24, 0.23)	**-0.052 (-0.081, -0.023)**	**<0.001**	11634.8 (7980.8, 16142.9)	45.0 (31.5, 61.9)	-0.42 (-0.61, -0.21)	0.01 (-0.26, 0.25)	**-1.160 (-1.711, -0.608)**	**<0.001**
Sudan	2159.1 (1462.1, 3119.7)	13.0 (8.8, 18.5)	-0.38 (-0.51, -0.18)	0.05 (-0.02, 0.12)	**-0.512 (-0.686, -0.338)**	**<0.001**	54988.6 (36307.1, 81547.1)	264.0 (177.4, 383.1)	-0.43 (-0.57, -0.21)	0.04 (-0.03, 0.11)	**-12.002 (-15.768, -8.237)**	**<0.001**

Suriname	1.7<br>(0.5, 2.6)	0.3<br>(0.1, 0.4)	-0.63<br>(-0.76, -0.04)	0.30<br>(0.00, 0.75)	**-0.012**<br>**(-0.018, -0.006)**	**<0.001**	40.1<br>(11.2, 60.2)	6.3<br>(1.8, 9.5)	-0.62<br>(-0.76, -0.02)	0.32<br>(0.03, 0.78)	**-0.266**<br>**(-0.401, -0.130)**	**<0.001**
Sweden	2600.8<br>(2188.6, 2907.1)	9.5<br>(8.1, 10.6)	-0.53<br>(-0.57, -0.48)	-0.04<br>(-0.11, 0.03)	**-0.407**<br>**(-0.434, -0.380)**	**<0.001**	34542.1<br>(29763.3, 38395.7)	143.6<br>(125.4, 159.1)	-0.58<br>(-0.62, -0.54)	-0.07<br>(-0.15, 0.01)	**-7.491**<br>**(-7.949, -7.032)**	**<0.001**
Switzerland	1989.1<br>(1611.8, 2227.4)	8.3<br>(6.9, 9.2)	-0.59<br>(-0.63, -0.57)	0.02<br>(-0.01, 0.04)	**-0.420**<br>**(-0.452, -0.388)**	**<0.001**	25515.1<br>(21575.9, 28220.9)	122.5<br>(106.6, 133.8)	-0.64<br>(-0.67, -0.62)	0.01<br>(-0.01, 0.03)	**-7.703**<br>**(-8.317, -7.090)**	**<0.001**
Syrian Arab Republic	4222.2<br>(3343.7, 5340.7)	42.4<br>(34.4, 52.1)	-0.24<br>(-0.42, -0.01)	-0.11<br>(-0.20, -0.02)	**-0.660**<br>**(-0.791, -0.529)**	**<0.001**	96828.5<br>(74897.5, 125542.7)	788.1<br>(623.9, 1001.9)	-0.33<br>(-0.51, -0.08)	-0.11<br>(-0.21, -0.01)	**-17.241**<br>**(-20.120, -14.361)**	**<0.001**
Taiwan (Province of China)	2358.1<br>(1971.7, 2684.5)	5.3<br>(4.5, 6.1)	-0.59<br>(-0.63, -0.55)	0.07<br>(-0.01, 0.11)	**-0.263**<br>**(-0.302, -0.224)**	**<0.001**	39958.4<br>(34393.9, 45016.2)	96.1<br>(83.4, 108.0)	-0.59<br>(-0.63, -0.56)	0.06<br>(-0.01, 0.11)	**-4.806**<br>**(-5.524, -4.089)**	**<0.001**
Tajikistan	1607.5<br>(1384.3, 1841.7)	38.0<br>(32.8, 43.3)	-0.26<br>(-0.37, -0.16)	-0.01<br>(-0.10, 0.09)	**-0.727**<br>**(-0.928, -0.526)**	**<0.001**	36779.8<br>(31515.7, 42521.0)	695.7<br>(598.9, 798.6)	-0.32<br>(-0.42, -0.22)	-0.00<br>(-0.10, 0.10)	**-16.896**<br>**(-20.338, -13.454)**	**<0.001**
Thailand	2424.0<br>(1570.7, 3442.5)	2.3<br>(1.5, 3.2)	-0.01<br>(-0.26, 0.35)	0.87<br>(0.57, 1.26)	**-0.062**<br>**(-0.095, -0.029)**	**<0.001**	50015.0<br>(32199.2, 71177.5)	48.5<br>(31.2, 69.2)	0.05<br>(-0.23, 0.43)	0.86<br>(0.54, 1.26)	**-1.246**<br>**(-1.939, -0.552)**	**<0.001**
Timor-Leste	37.0<br>(27.9, 47.6)	5.1<br>(3.8, 6.6)	0.05<br>(-0.27, 0.75)	-0.28<br>(-0.33, -0.21)	0.043<br>(-0.002, 0.087)	0.058	853.2<br>(642.5, 1099.4)	100.9<br>(76.2, 129.5)	0.00<br>(-0.31, 0.69)	-0.27<br>(-0.33, -0.20)	0.658<br>(-0.214, 1.530)	0.134
Togo	19.8<br>(8.9, 33.4)	0.7<br>(0.3, 1.1)	-0.68<br>(-0.79, -0.40)	-0.28<br>(-0.37, 0.00)	**-0.053**<br>**(-0.070, -0.037)**	**<0.001**	534.2<br>(235.3, 914.6)	13.5<br>(6.1, 22.7)	-0.69<br>(-0.80, -0.40)	-0.28<br>(-0.37, -0.01)	**-1.136**<br>**(-1.486, -0.787)**	**<0.001**
Tokelau	0.0<br>(0.0, 0.0)	0.1<br>(0.1, 0.2)	-0.30<br>(-0.50, -0.03)	1.32<br>(1.13, 1.96)	**-0.005**<br>**(-0.008, -0.002)**	**0.003**	0.0<br>(0.0, 0.1)	2.6<br>(1.7, 3.7)	-0.28<br>(-0.48, 0.08)	1.45<br>(1.15, 2.25)	**-0.101**<br>**(-0.164, -0.038)**	**0.003**
Tonga	0.1<br>(0.0, 0.2)	0.1<br>(0.0, 0.2)	-0.46<br>(-0.63, 0.80)	0.52<br>(0.29, 0.82)	**-0.012**<br>**(-0.020, -0.004)**	**0.006**	2.3<br>(0.7, 4.1)	2.9<br>(0.9, 5.0)	-0.47<br>(-0.65, 0.74)	0.51<br>(0.29, 0.84)	**-0.251**<br>**(-0.426, -0.077)**	**0.006**
Trinidad and Tobago	4.7<br>(1.0, 7.8)	0.3<br>(0.1, 0.4)	-0.79<br>(-0.91, -0.71)	0.47<br>(0.13, 2.38)	**-0.030**<br>**(-0.038, -0.022)**	**<0.001**	100.2<br>(21.2, 168.7)	5.4<br>(1.1, 9.0)	-0.76<br>(-0.89, -0.67)	0.46<br>(0.11, 2.31)	**-0.582**<br>**(-0.740, -0.423)**	**<0.001**
Tunisia	2801.9<br>(2163.9, 3622.9)	24.0<br>(18.7, 30.7)	-0.25<br>(-0.41, -0.02)	-0.09<br>(-0.15, -0.00)	**-0.373**<br>**(-0.431, -0.316)**	**<0.001**	53897.7<br>(41059.0, 71091.1)	425.8<br>(328.1, 556.8)	-0.28<br>(-0.45, -0.03)	-0.09<br>(-0.16, 0.01)	**-7.502**<br>**(-8.513, -6.490)**	**<0.001**
Turkey	23208.2<br>(19162.5, 27209.1)	27.9<br>(23.0, 32.5)	-0.42<br>(-0.51, -0.32)	-0.01<br>(-0.09, 0.07)	**-0.586**<br>**(-0.739, -0.432)**	**<0.001**	423414.0<br>(349192.4, 501105.1)	475.7<br>(392.6, 561.3)	-0.49<br>(-0.58, -0.39)	-0.01<br>(-0.10, 0.09)	**-15.265**<br>**(-17.982, -12.548)**	**<0.001**
Turkmenistan	1755.1<br>(1412.8, 2198.3)	50.9<br>(41.3, 63.5)	-0.13<br>(-0.29, 0.06)	-0.02<br>(-0.11, 0.09)	**-0.763**<br>**(-0.999, -0.527)**	**<0.001**	42248.7<br>(33514.7, 53190.4)	1059.5<br>(847.9, 1324.1)	-0.12<br>(-0.29, 0.09)	-0.02<br>(-0.12, 0.10)	**-15.021**<br>**(-20.200, -9.843)**	**<0.001**
Tuvalu	0.0<br>(0.0, 0.0)	0.2<br>(0.1, 0.2)	-0.49<br>(-0.60, -0.21)	0.04<br>(-0.01, 0.10)	**-0.016**<br>**(-0.022, -0.009)**	**<0.001**	0.4<br>(0.3, 0.5)	4.1<br>(2.9, 5.2)	-0.50<br>(-0.61, -0.21)	0.04<br>(-0.02, 0.11)	**-0.359**<br>**(-0.505, -0.212)**	**<0.001**

Uganda	737.7 (583.7, 928.9)	6.1 (4.9, 7.6)	-0.45 (-0.58, -0.26)	-0.06 (-0.12, 0.04)	**-0.222 (-0.266, -0.178)**	**<0.001**	19267.7 (14911.8, 24174.3)	121.8 (95.9, 153.5)	-0.46 (-0.60, -0.26)	-0.06 (-0.12, 0.04)	**-4.805 (-5.759, -3.850)**	**<0.001**
Ukraine	30865.5 (24128.2, 37785.6)	39.3 (30.7, 48.1)	-0.18 (-0.36, 0.01)	0.07 (-0.18, 0.39)	**-0.891 (-1.113, -0.669)**	**<0.001**	555956.1 (421858.0, 692596.7)	736.8 (558.2, 920.0)	-0.15 (-0.35, 0.07)	0.07 (-0.22, 0.42)	**-15.683 (-19.934, -11.433)**	**<0.001**
United Arab Emirates	525.1 (343.9, 708.1)	31.1 (21.1, 41.7)	-0.48 (-0.56, -0.40)	-0.43 (-0.53, -0.34)	-0.058 (-0.433, 0.318)	0.756	16134.7 (10550.1, 21882.3)	533.4 (359.4, 713.3)	-0.57 (-0.64, -0.49)	-0.43 (-0.53, -0.33)	**-10.591 (-16.417, -4.766)**	**<0.001**
United Kingdom	15129.1 (13171.6, 16865.4)	10.1 (8.9, 11.2)	-0.59 (-0.62, -0.56)	-0.05 (-0.06, -0.04)	**-0.534 (-0.585, -0.483)**	**<0.001**	229126.3 (205182.2, 252362.5)	172.5 (156.6, 189.3)	-0.61 (-0.64, -0.59)	-0.05 (-0.07, -0.04)	**-10.099 (-11.127, -9.071)**	**<0.001**
United Republic of Tanzania	1741.9 (1420.6, 2191.0)	8.2 (6.7, 10.2)	-0.14 (-0.31, 0.18)	0.17 (0.09, 0.29)	**-0.068 (-0.093, -0.043)**	**<0.001**	43087.3 (34150.6, 54583.5)	162.2 (130.7, 204.7)	-0.18 (-0.36, 0.17)	0.17 (0.08, 0.29)	**-1.705 (-2.214, -1.195)**	**<0.001**
United States of America	91668.4 (78721.9, 99652.0)	14.7 (12.8, 15.9)	-0.33 (-0.37, -0.31)	-0.05 (-0.06, -0.04)	**-0.323 (-0.360, -0.285)**	**<0.001**	1593235.4 (1428744.4, 1705149.2)	280.3 (255.4, 298.6)	-0.34 (-0.37, -0.32)	-0.06 (-0.07, -0.04)	**-6.187 (-6.877, -5.496)**	**<0.001**
United States Virgin Islands	0.7 (0.5, 0.9)	0.4 (0.3, 0.6)	-0.76 (-0.82, -0.50)	-0.35 (-0.39, -0.31)	**-0.032 (-0.043, -0.020)**	**<0.001**	12.5 (8.7, 17.4)	8.0 (5.6, 11.0)	-0.75 (-0.82, -0.49)	-0.35 (-0.40, -0.31)	**-0.608 (-0.830, -0.387)**	**<0.001**
Uruguay	1028.0 (919.7, 1109.3)	16.1 (14.5, 17.3)	-0.46 (-0.48, -0.44)	-0.03 (-0.05, -0.01)	**-0.451 (-0.499, -0.402)**	**<0.001**	16646.2 (15234.3, 17832.5)	295.5 (274.0, 315.6)	-0.48 (-0.50, -0.46)	-0.03 (-0.05, -0.01)	**-9.276 (-10.244, -8.309)**	**<0.001**
Uzbekistan	9141.7 (7778.1, 10779.8)	44.3 (37.8, 52.1)	-0.04 (-0.16, 0.08)	0.00 (-0.10, 0.10)	**-0.202 (-0.377, -0.028)**	**0.025**	208971.1 (178111.1, 246933.4)	847.4 (721.2, 999.9)	-0.09 (-0.22, 0.03)	0.00 (-0.11, 0.11)	**-6.686 (-10.223, -3.149)**	**<0.001**
Vanuatu	6.6 (4.9, 8.3)	4.5 (3.3, 5.7)	-0.16 (-0.35, 0.15)	-0.20 (-0.25, -0.11)	**-0.095 (-0.154, -0.035)**	**0.003**	189.7 (141.2, 241.5)	101.6 (76.0, 128.5)	-0.16 (-0.38, 0.19)	-0.20 (-0.26, -0.10)	**-2.147 (-3.491, -0.802)**	**0.003**
Venezuela (Bolivarian Republic of)	1432.9 (1114.4, 1813.8)	5.1 (4.0, 6.4)	-0.11 (-0.29, 0.11)	0.31 (0.10, 0.58)	**-0.081 (-0.126, -0.036)**	**<0.001**	30398.9 (23288.8, 39043.3)	102.6 (79.2, 131.2)	-0.12 (-0.32, 0.11)	0.33 (0.10, 0.62)	**-1.793 (-2.723, -0.863)**	**<0.001**
Viet Nam	6522.0 (5099.4, 8084.1)	7.8 (6.2, 9.6)	-0.00 (-0.20, 0.24)	0.32 (0.26, 0.39)	-0.026 (-0.077, 0.025)	0.310	136706.3 (104141.3, 173494.0)	144.9 (111.9, 181.2)	-0.05 (-0.26, 0.21)	0.32 (0.25, 0.40)	-0.658 (-1.627, 0.312)	0.176
Yemen	2569.7 (1991.4, 3289.8)	22.7 (17.9, 28.4)	-0.19 (-0.38, 0.05)	0.11 (0.04, 0.18)	**-0.311 (-0.385, -0.236)**	**<0.001**	63928.0 (48580.4, 82850.3)	448.9 (346.0, 576.7)	-0.25 (-0.44, -0.01)	0.10 (0.03, 0.18)	**-7.834 (-9.391, -6.276)**	**<0.001**
Zambia	904.5 (679.3, 1133.3)	16.3 (12.8, 20.1)	0.08 (-0.17, 0.59)	0.16 (0.08, 0.28)	-0.029 (-0.080, 0.022)	0.255	24467.1 (18064.1, 31297.7)	330.8 (249.2, 414.0)	0.05 (-0.22, 0.59)	0.18 (0.09, 0.29)	-1.111 (-2.242, 0.021)	0.054
Zimbabwe	1142.9 (920.2, 1403.2)	21.0 (17.3, 24.9)	0.37 (0.11, 0.75)	0.08 (0.02, 0.14)	**0.245 (0.113, 0.376)**	**<0.001**	30176.4 (23582.7, 38041.0)	433.6 (349.5, 531.4)	0.51 (0.18, 0.99)	0.12 (0.05, 0.18)	**5.828 (2.994, 8.663)**	**<0.001**

ASMR, age-standardized mortality rates; ASRDALYs, age-standardized rates of DALYs; DALYs, disability-adjusted life-years.

Table S11. Decomposition analysis results of non-communicable diseases attributable to high and low temperatures.

Location	Cause	Measure	Overall difference	Aging	Population	Epidemiological change
**Global**	High temperature	Deaths	204289.5	79204.7(38.8%)	71085.4(34.8%)	53999.3(26.4%)
High SDI	High temperature	Deaths	10189.0	6874.8(67.5%)	3237.5(31.8%)	76.8(0.8%)
High-middle SDI	High temperature	Deaths	18586.1	11192.2(60.2%)	3810.3(20.5%)	3583.6(19.3%)
Middle SDI	High temperature	Deaths	68827.3	37375.2(54.3%)	19128.5(27.8%)	12323.6(17.9%)
Low-middle SDI	High temperature	Deaths	88187.7	30936.2(35.1%)	38317.6(43.5%)	18934(21.5%)
Low SDI	High temperature	Deaths	18407.7	794.5(4.3%)	14104.2(76.6%)	3509(19.1%)
**Global**	High temperature	DALYs	4456855.2	1490632.6(33.5%)	1694944.9(38.0%)	1271277.7(28.5%)
High SDI	High temperature	DALYs	250410.8	102948.9(41.1%)	70717.8(28.2%)	76744(30.7%)
High-middle SDI	High temperature	DALYs	306771.9	177902.2(58.0%)	74492.2(24.3%)	54377.5(17.7%)
Middle SDI	High temperature	DALYs	1417649.8	709424.3(50.0%)	440579.6(31.1%)	267646(18.9%)
Low-middle SDI	High temperature	DALYs	2022939.5	637428.5(31.5%)	964475.2(47.7%)	421035.9(20.8%)
Low SDI	High temperature	DALYs	457107.0	10433.9(2.3%)	375340.2(82.1%)	71332.9(15.6%)
**Global**	Low temperature	Deaths	441500.7	650150.7(147.3%)	509028.6(115.3%)	-717678.5(-162.6%)
High SDI	Low temperature	Deaths	17562.0	159303.4(907.1%)	62364.1(355.1%)	-204105.5(-1162.2%)
High-middle SDI	Low temperature	Deaths	95884.1	276342.8(288.2%)	88745.9(92.6%)	-269204.5(-280.8%)
Middle SDI	Low temperature	Deaths	200082.5	297633.7(148.8%)	142421.4(71.2%)	-239972.6(-119.9%)
Low-middle SDI	Low temperature	Deaths	102739.6	55996.5(54.5%)	68280.5(66.5%)	-21537.5(-21.0%)
Low SDI	Low temperature	Deaths	25216.0	2066(8.2%)	36513.9(144.8%)	-13363.9(-53.0%)
**Global**	Low temperature	DALYs	5772599.3	10563548.8(183.0%)	10152563.7(175.9%)	-14943513.2(-258.9%)
High SDI	Low temperature	DALYs	-304548.5	2122533.9(-696.9%)	1051017.6(-345.1%)	-3478099.9(1142.1%)
High-middle SDI	Low temperature	DALYs	572765.8	4276812.2(746.7%)	1654656.2(288.9%)	-5358702.6(-935.6%)
Middle SDI	Low temperature	DALYs	2729214.7	5299753.2(194.2%)	2994931.7(109.7%)	-5565470.1(-203.9%)

Low-middle SDI	Low temperature	DALYs	2224526.9	1139313.9(51.2%)	1680550.7(75.6%)	-595337.8(-26.8%)
Low SDI	Low temperature	DALYs	553358.3	31426.2(5.7%)	952092.3(172.1%)	-430160.1(-77.7%)

DALYs, disability-adjusted life-years; SDI = socio-demographic index.

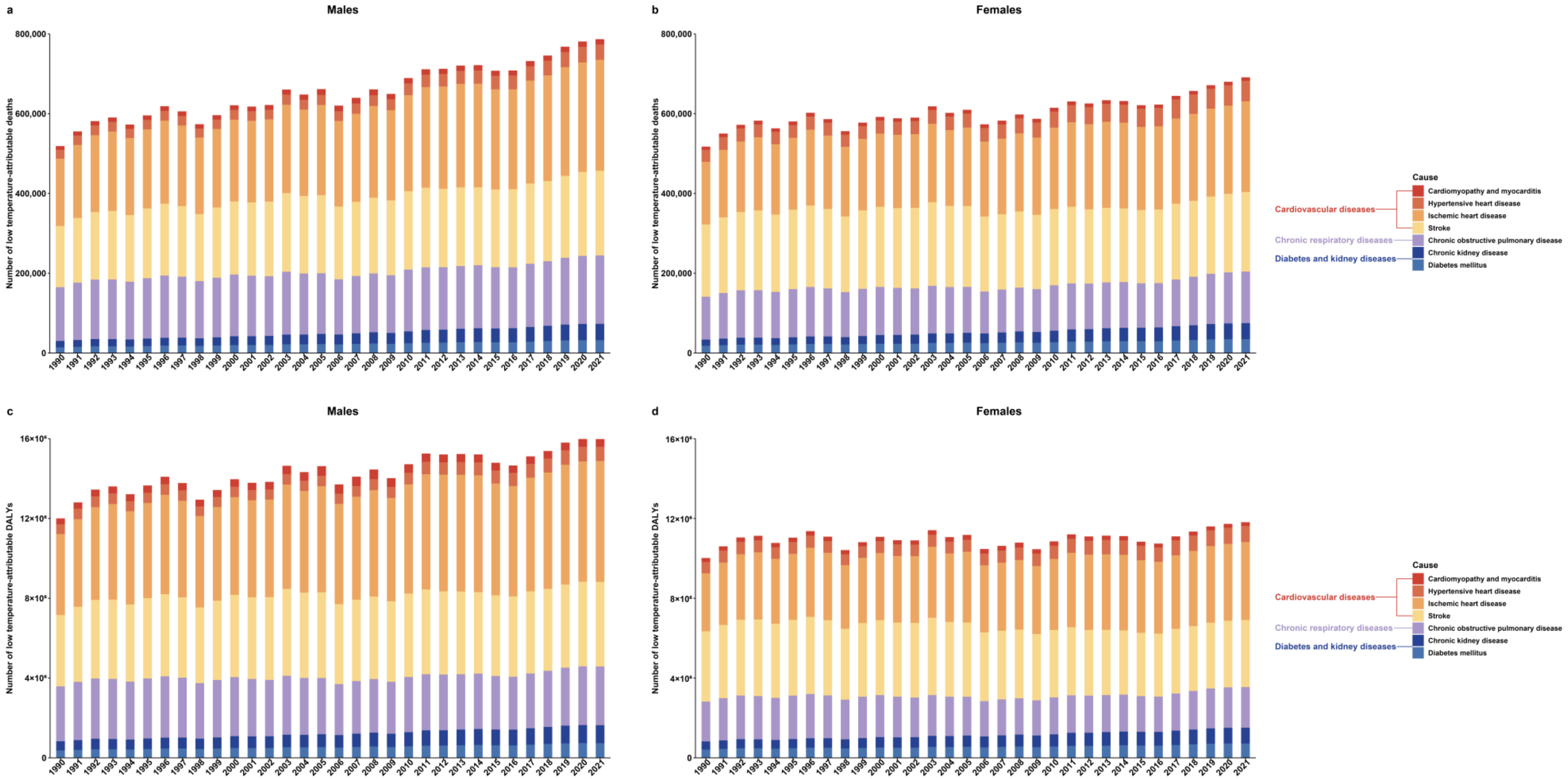

Fig. S1. Numbers of all-age deaths and disability-adjusted life-years (DALYs) in non-communicable diseases attributable to low temperature by sex from 1990 to 2021 globally. (a) Numbers of all-age deaths in males. (b) Numbers of all-age deaths in females. (c) Numbers of all-age DALYs in males. (d) Numbers of all-age DALYs in females.

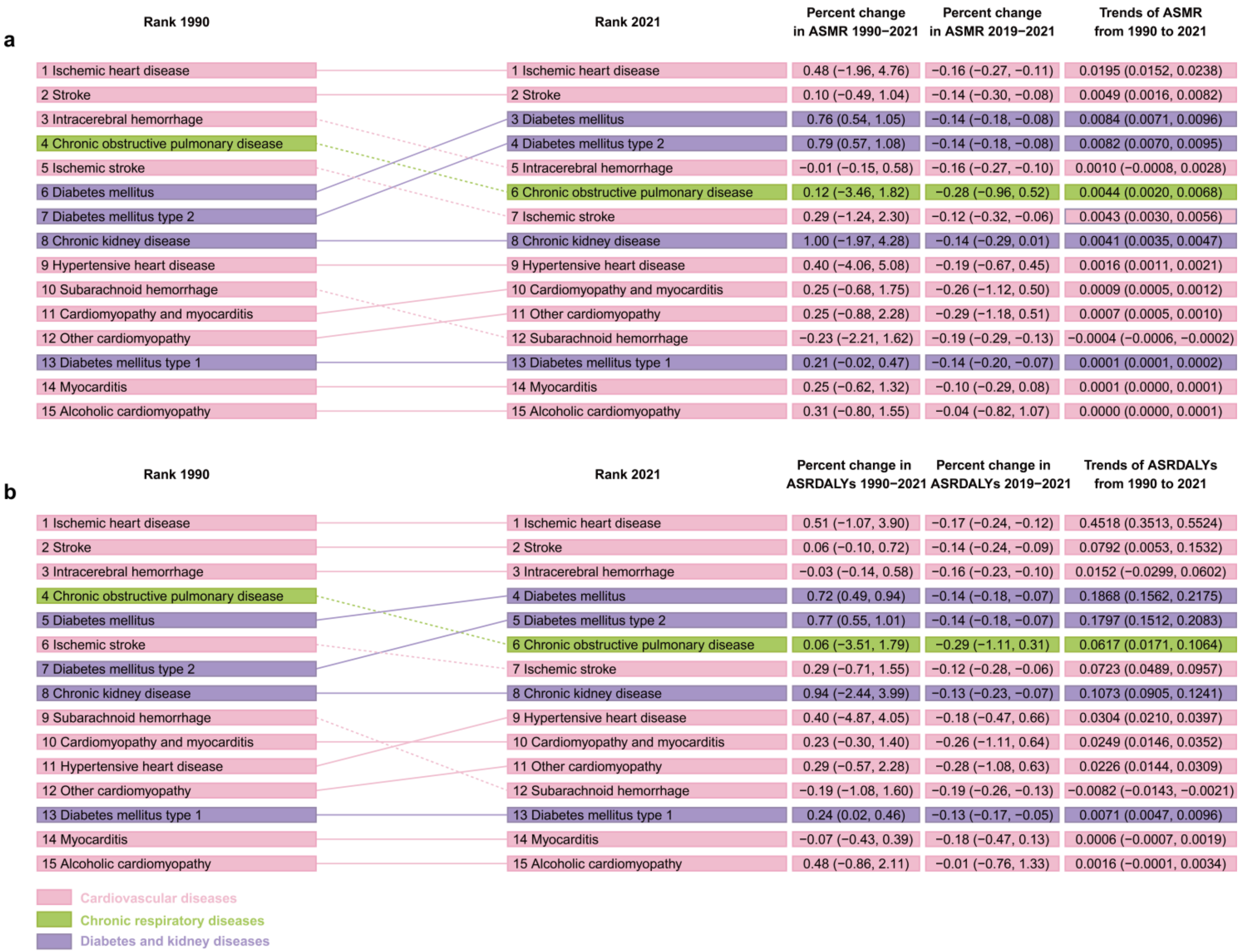

Fig. S2. Leading level 3 and level 4 causes of global non-communicable diseases attributable to high temperature rankings by age-standardized mortality rate (ASMR) and age-standardized rate of disability-adjusted life years (ASRDALYs) per 100,000 population, 1990 and 2021. Causes are connected by lines between time periods. Solid lines represent an increase or lateral shift in ranking, and dashed lines represent decreases in rank. (a) Rankings by ASMR. (b) Rankings by ASRDALYs

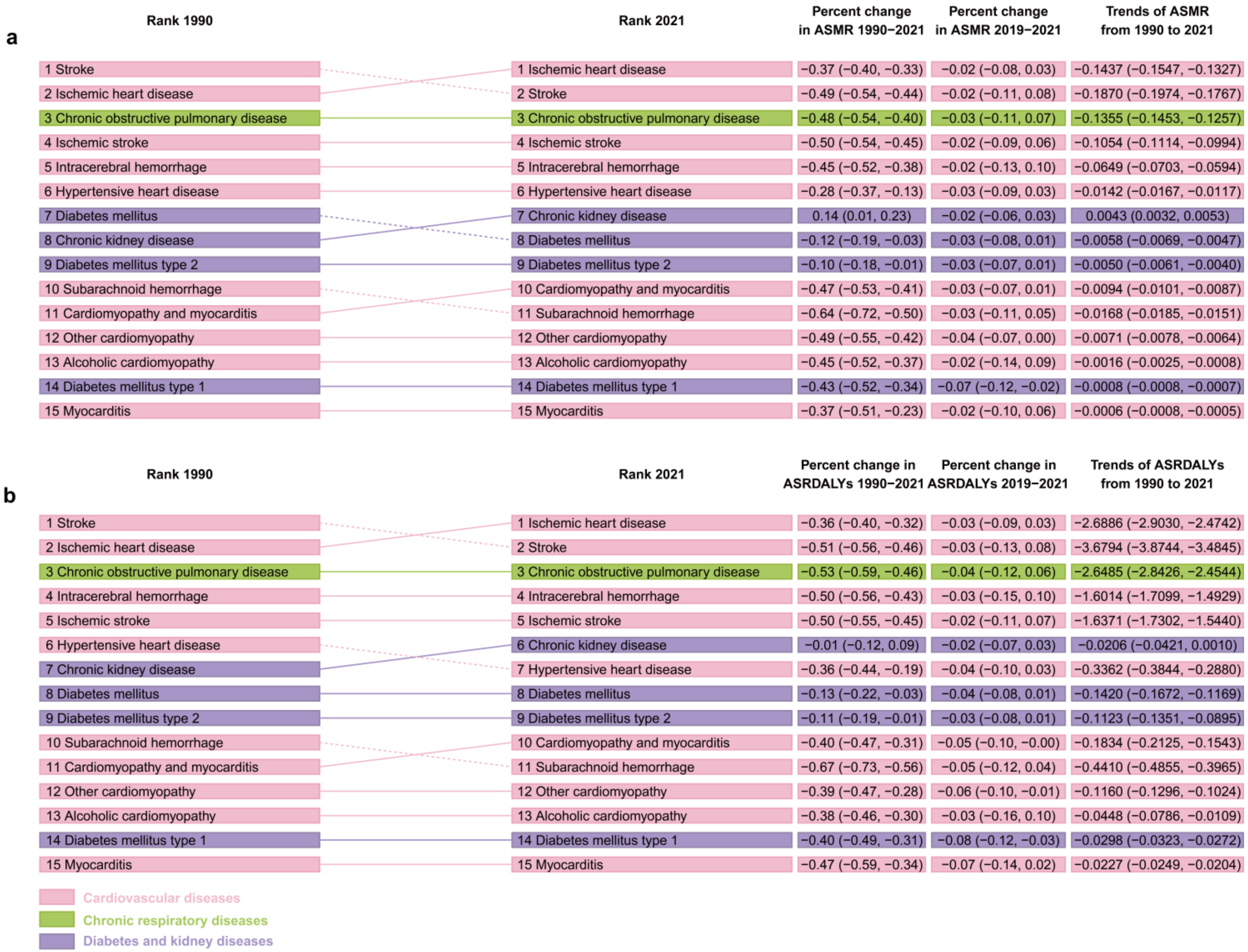

Fig. S3. Leading level 3 and level 4 causes of global non-communicable diseases attributable to low temperature rankings by age-standardized mortality rate (ASMR) and age-standardized rate of disability-adjusted life years (ASRDALYs) per 100,000 population, 1990 and 2021. Causes are connected by lines between time periods. Solid lines represent an increase or lateral shift in ranking, and dashed lines represent decreases in rank. (a) Rankings by ASMR. (b) Rankings by ASRDALYs

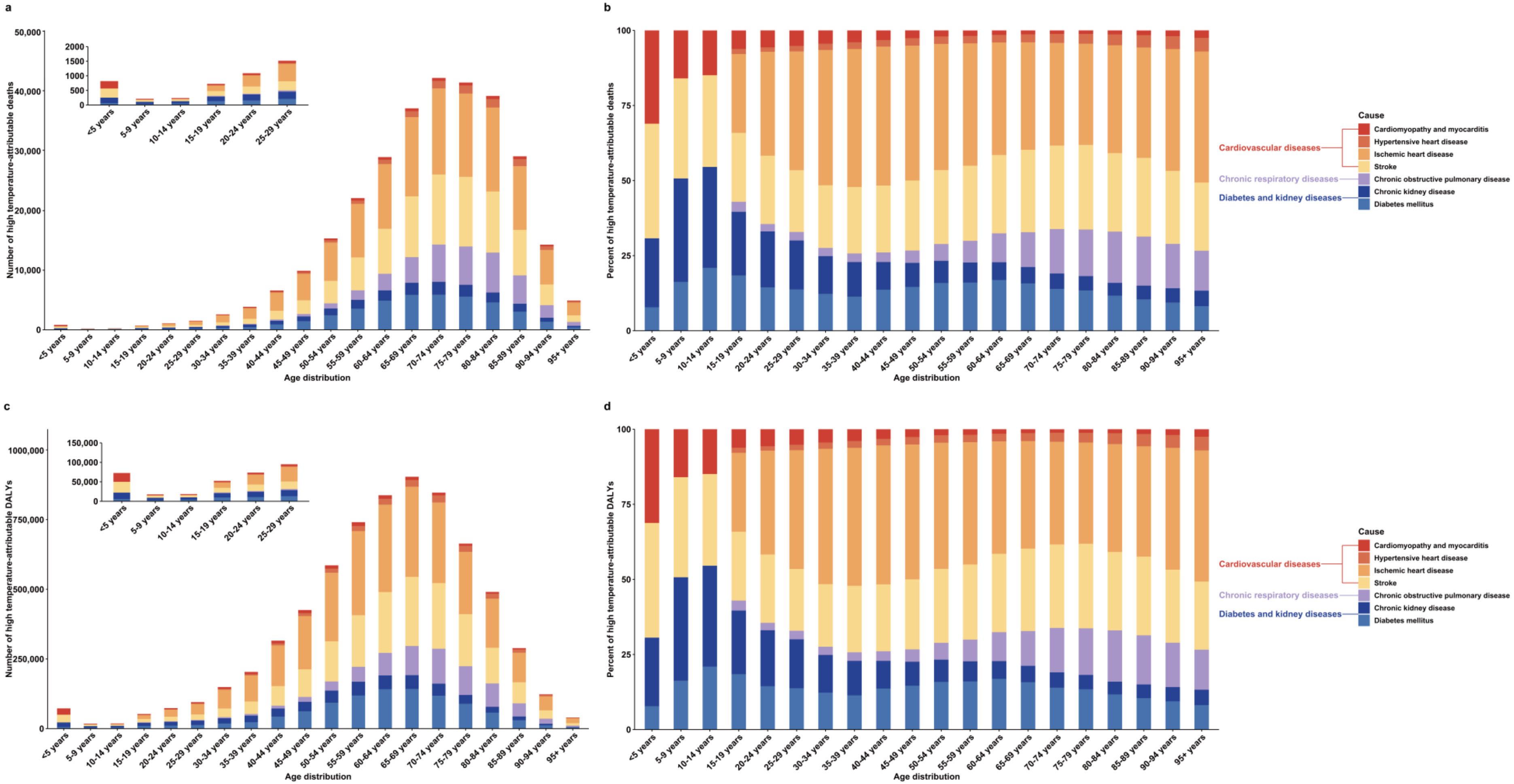

Fig. S4. Numbers and proportion of all-age deaths and disability-adjusted life-years (DALYs) in non-communicable diseases attributable to high temperature in 2021 globally. (a) Number of deaths by level 3 causes and age distribution. (b) Contribution of level 3 causes for deaths by age distribution. (c) Number of DALYs by level 3 causes and age distribution. (d) Contribution of level 3 causes for DALYs by age distribution.

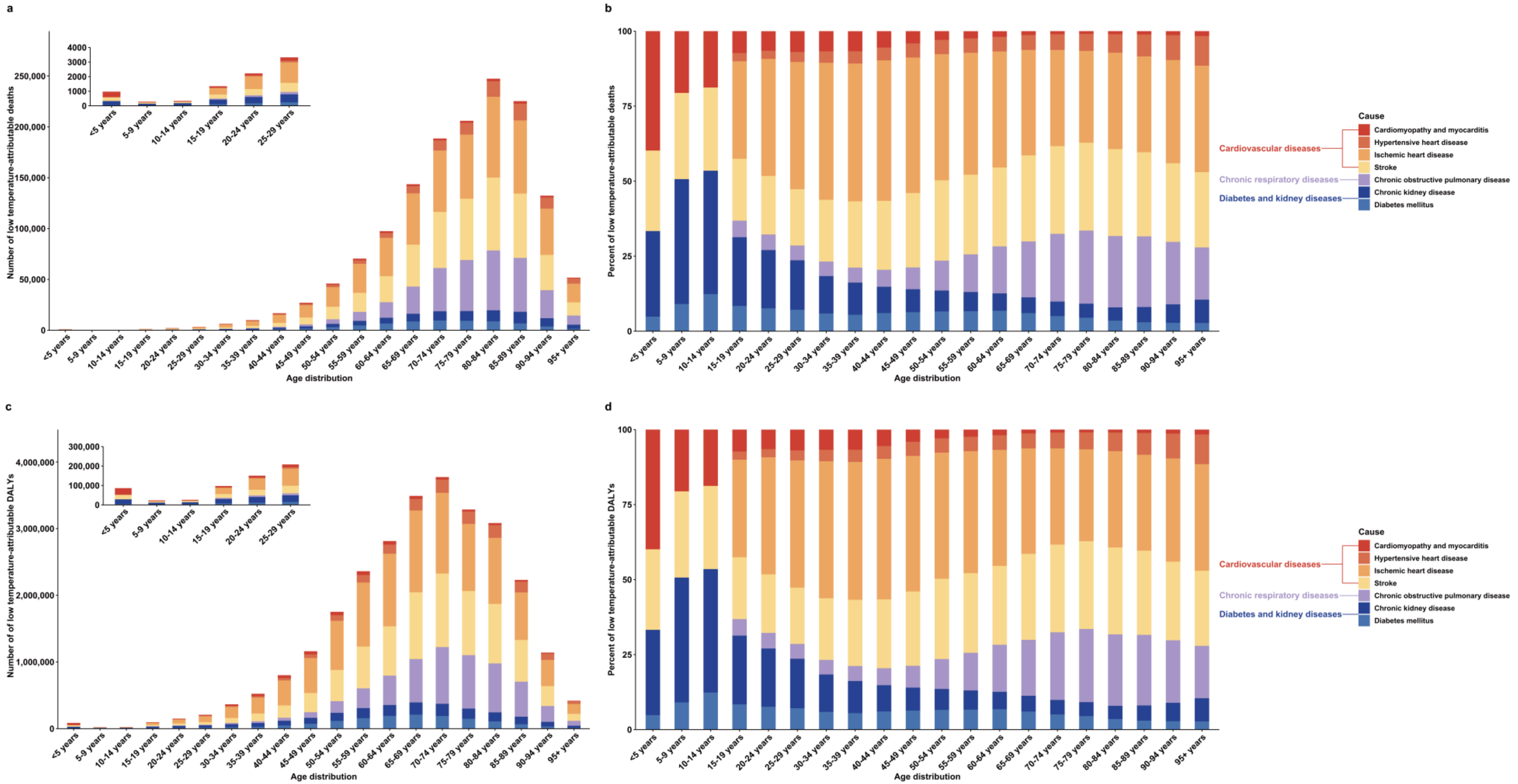

Fig. S5. Numbers and proportion of all-age deaths and disability-adjusted life-years (DALYs) in non-communicable diseases attributable to low temperature in 2021 globally. (a) Number of deaths by level 3 causes and age distribution. (b) Contribution of level 3 causes for deaths by age distribution. (c) Number of DALYs by level 3 causes and age distribution. (d) Contribution of level 3 causes for DALYs by age distribution.

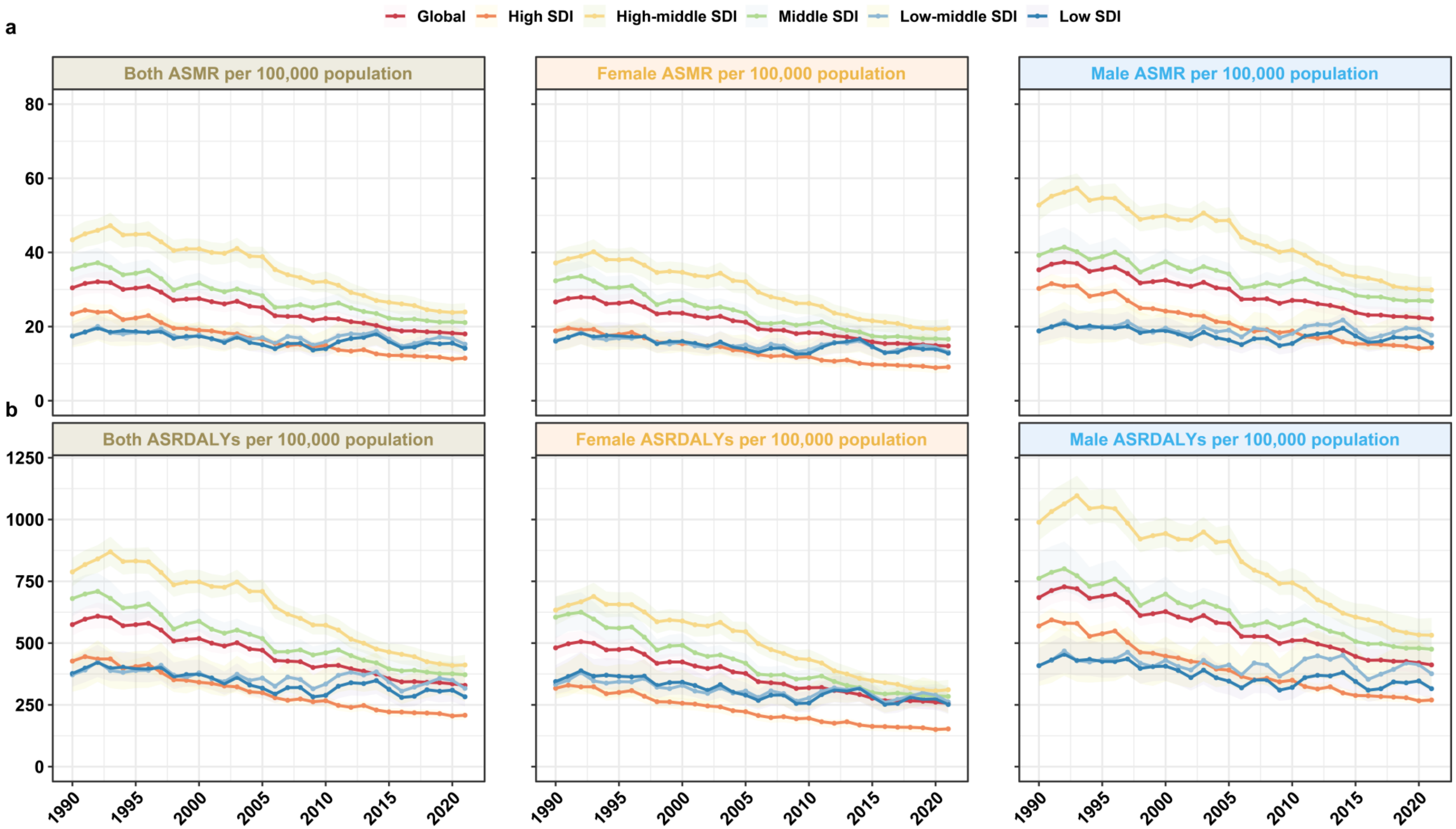

Fig. S6. Temporal trends of non-communicable diseases (NCDs) burden due to low temperature in sex, global, and socio-demographic index level from 1990 to 2021. (a) The age-standardized mortality rate of NCDs attributable to low temperature. (b) The age-standardized rate of disability-adjusted life-years due to NCDs associated with low temperature.

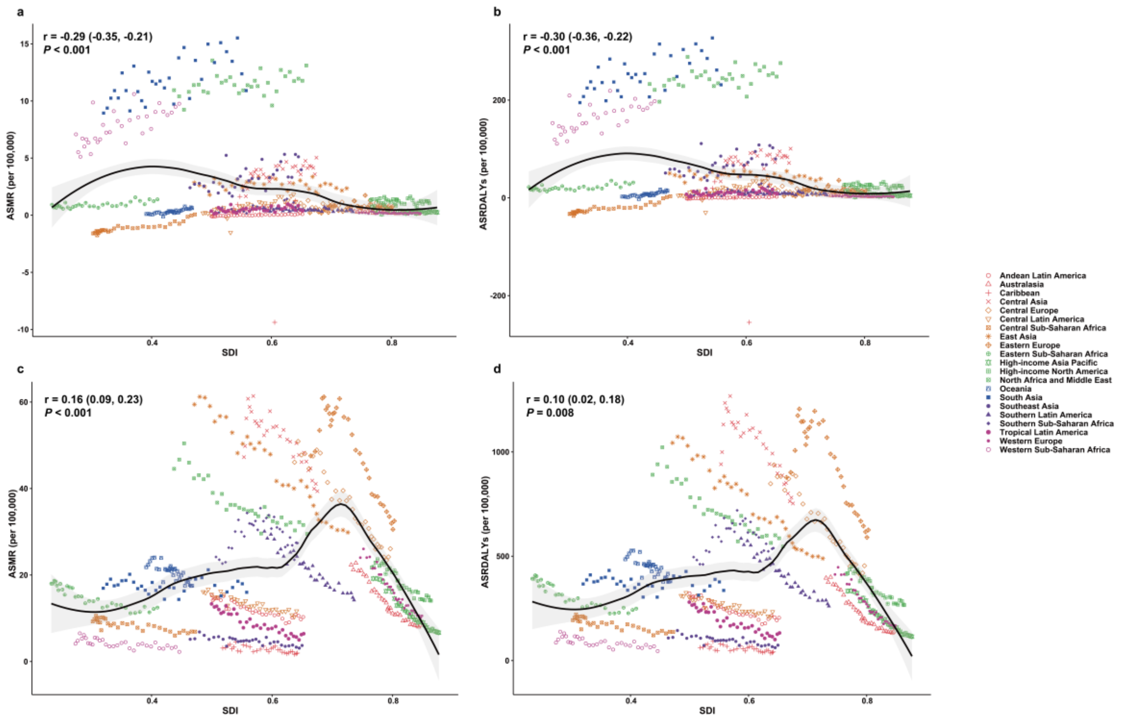

Fig. S7. Age-standardized mortality rates (ASMR) and age-standardized rates of disability-adjusted life-years (ASRDALYs) of non-communicable diseases (NCDs) due to high temperature and low temperature for 21 GBD regions, by socio-demographic index, from 1990 to 2021. (a) ASMR of NCDs due to high temperature. (b) ASRDALYs of NCDs due to high temperature. (c) ASMR of NCDs due to low temperature. (d) ASMR of NCDs due to low temperature.

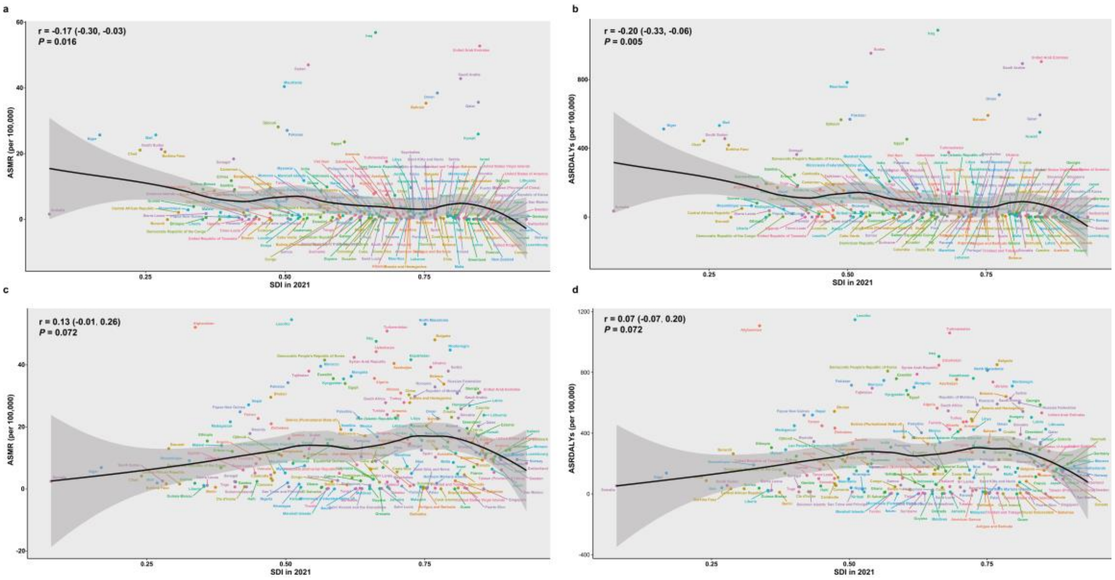

Fig. S8. Age-standardized mortality rates (ASMR) and age-standardized rates of disability-adjusted life-years (ASRDALYs) of non-communicable diseases (NCDs) due to high temperature and low temperature for 204 countries and territories, by socio-demographic index (SDI) in 2021. (a) ASMR of NCDs due to high temperature by SDI. (b) ASRDALYs of NCDs due to high temperature by SDI. (c) ASMR of NCDs due to low temperature by SDI. (d) ASMR of NCDs due to low temperature by SDI.

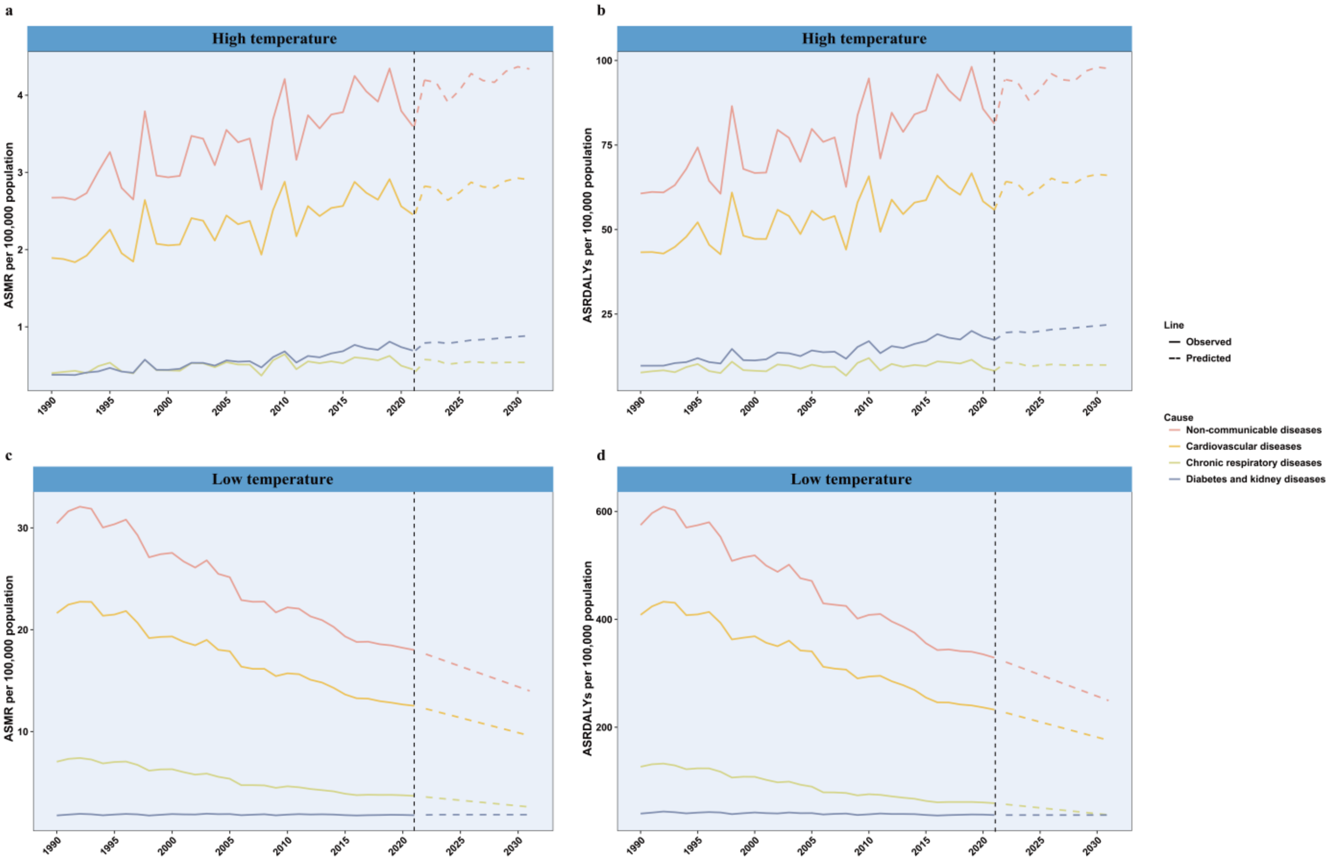

Fig. S9. Forecasted value of global age-standardized mortality rates (ASMR) and age-standardized rates of disability-adjusted life-years (ASRDALYs) of non-communicable diseases (NCDs) due to high temperature and low temperature for the period 2022 to 2031. (a) Forecasted value of ASMR due to high temperature. (b) Forecasted value of ASRDALYs due to high temperature. (c) Forecasted value of ASMR due to low temperature. (d) Forecasted value of ASRDALYs due to low temperature.
